# Design, Synthesis, and Biological Evaluation of 8-Mercapto-3,7-Dihydro-1*H*-Purine-2,6-Diones as Potent Inhibitors of SIRT1, SIRT2, SIRT3, and SIRT5

**DOI:** 10.3390/molecules25122755

**Published:** 2020-06-15

**Authors:** Haozhen Han, Chunpu Li, Man Li, Lisheng Yang, Sen Zhao, Zhifei Wang, Hong Liu, Dongxiang Liu

**Affiliations:** 1 Department of Pharmacology III, Shanghai Institute of Materia Medica, Chinese Academy of Sciences, 555 Zu-Chong-Zhi Road, Shanghai 201203, China; hhaozhen@126.com; 2 College of Pharmacy, University of Chinese Academy of Sciences, No. 19A Yuquan Road, Beijing 100049, China; 3 Department of Medicinal Chemistry, Shanghai Institute of Materia Medica, Chinese Academy of Sciences, 555 Zu-Chong-Zhi Road, Shanghai 201203, China; lcp1681993@163.com (C.L.); 15250968801@163.com (L.Y.); zhaosen@shu.edu.cn (S.Z.); 4 School of Basic Medicine, Shanghai University of Traditional Chinese Medicine, Shanghai 201203, China; liman1451098759@163.com

**Keywords:** sirtuins, inhibitor, structure–activity relationship, deacetylase

## Abstract

Sirtuins (SIRT1-7) are a family of NAD^+^-dependent deacetylases. They regulate many physiological processes and play important roles in inflammation, diabetes, cancers, and neurodegeneration diseases. Sirtuin inhibitors have potential applications in the treatment of neurodegenerative diseases and various cancers. Herein, we identified new sirtuin inhibitors based on the scaffold of 8-mercapto-3,7-dihydro-1*H*-purine-2,6-dione. To elucidate the inhibitory mechanism, the binding modes of the inhibitors in SIRT3 were established by molecular docking, showing that the inhibitors occupy the acetyl lysine binding site and interact with SIRT3, mainly through hydrophobic interactions. The interactions were validated by site-directed mutagenesis of SIRT3 and structure–activity relationship analysis of the inhibitors. Consistently, enzyme kinetic assays and microscale thermophoresis showed that these compounds are competitive inhibitors to the acetyl substrate, and mix-type inhibitors to NAD^+^. Furthermore, we demonstrated that the compounds are potent SIRT1/2/3/5 pan-inhibitors. This study provides novel hits for developing more potent sirtuin inhibitors.

## 1. Introduction

Sirtuins are nicotinamide adenine dinucleotide (NAD^+^)-dependent deacylases that catalyze the deacylation of histones and non-histone proteins with the transformation of NAD^+^ into nicotinamide and 2’-*O*-acyl-adenosine diphosphate (ADP)-ribose [1]. Human sirtuins include seven family members, SIRT1–7, which have different subcellular localizations and functions [2]. SIRT1 [3], SIRT6 [4], and SIRT7 [5], which are mostly present in the nucleus, are associated with gene homeostasis, and regulate post-translational modification of proteins. SIRT2 is a cytoplasmic protein that co-localizes with microtubules and plays important roles in cell differentiation [6]. SIRT3 [7], SIRT4 [8], and SIRT5 [9], the mitochondrial sirtuins, participate in the regulation of aging and energy metabolism. Due to the critical roles of sirtuins in neurodegenerative diseases and different cancers [10], sirtuin inhibitors have potential applications in the treatment of Huntington’s disease and cancers [11]. This has aroused the interest of research groups around the world to develop sirtuin small molecule inhibitors.

**Figure 1:**
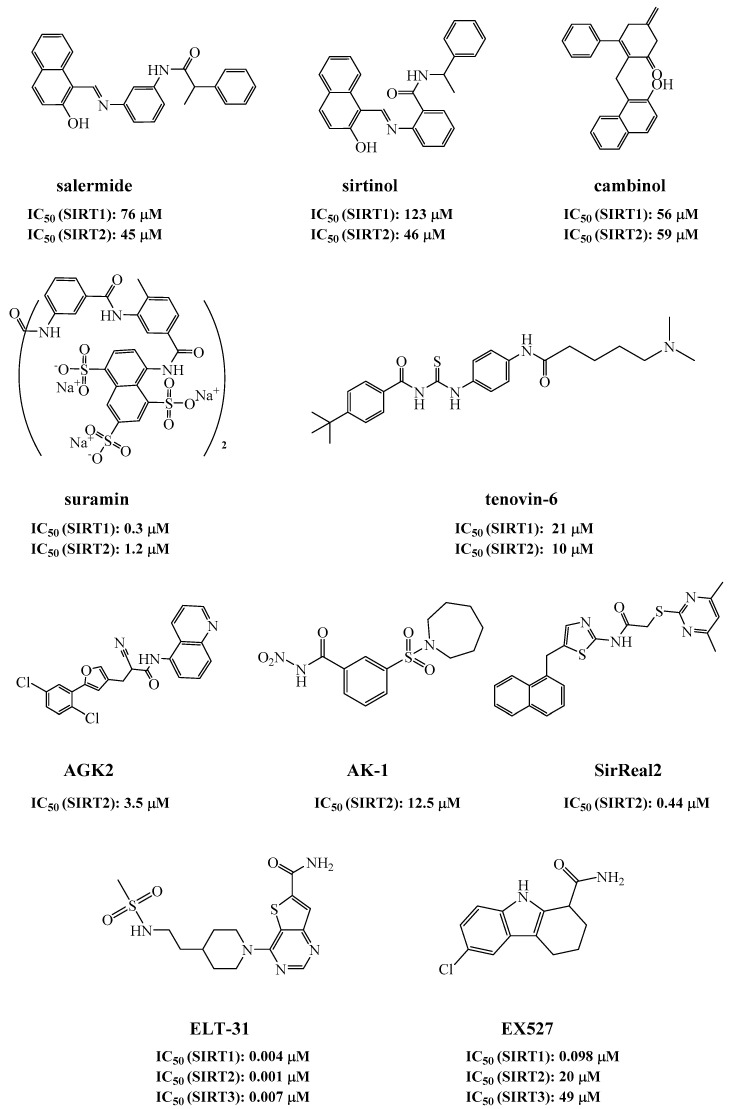
Sirtuin inhibitors.

So far, several sirtuin inhibitors have been reported (Figure 1). Among the inhibitors, salermide [12], sirtinol [13], cambinol [14], suramin [15], and tenovin-6 [16] are SIRT1 and SIRT2 inhibitors and exhibited antineoplastic, antiproliferative, or antiviral activities. AGK2 [17], AK-1 [18,19], and SirReal2 [20] are potent SIRT2 inhibitors and prevent the death of dopaminergic cells, hippocampal neurodegeneration, or induce cell cycle arrest in human colon carcinoma cells. ELT-31 is the most potent nanomolar SIRT1/2/3 inhibitor, which was discovered by screening the DNA encoded compound library [21,22]. Ex527, a SIRT1 inhibitor with IC_50_ of 60–100 nM, has been evaluated in clinical trials for the treatment of Huntington’s disease [23].

Despite the fact that several sirtuin inhibitors have been reported, most of them have limited or moderate inhibitory activities. Development of more potent inhibitors is needed, which can be used as tools to elucidate the biological functions of sirtuins, or as clinical interventions for the related diseases. In this study, novel sirtuin inhibitors with the scaffold of 8-mercapto-3,7-dihydro-1*H*-purine-2,6-dione was discovered through a fluorescent assay. The binding modes of the inhibitors in SIRT3 were established by molecular docking, which were confirmed by site-directed protein mutagenesis, structure–activity relationship (SAR) analysis, enzyme kinetic assays, and microscale thermophoresis (MST). In our study, ELT-31 was used as the control. Compared with ELT-31, the most potent sirtuin inhibitor ever reported [22], one of our inhibitors, **15**, exhibited better activities than ELT-31 for SIRT1 and SIRT5. 

## 2. Results and Discussion

### 2.1. Discovery of Sirtuin Inhibitors

**Figure 2:**
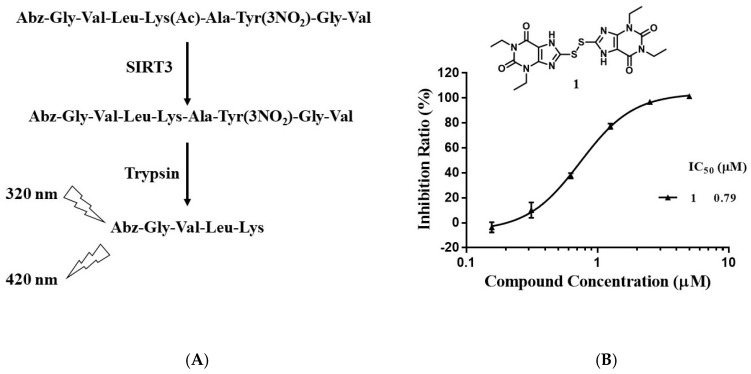
Discovery of SIRT3 inhibitors. (**A**) Principle of the enzyme assay. (**B**) Chemical structure and IC_50_ of compound **1**.

To discover new sirtuin inhibitors, we screened our in-house compound library (about 1100 compounds) by using a fluorescent assay. An internally quenched fluorescent peptide (sequence: Abz-GVLK_Ac_AY_NO2_GV-NH_2_; Abz: 2-aminobenzoyl; K_Ac_, acetyl lysine; Y_NO2_, 3-nitro-L-tyrosine) was used as the substrate [24]. After deacetylation by SIRT3, the substrate was hydrolyzed by trypsin to produce a fluorescent Abz-peptide (sequence: Abz-GVLK), which can be quantified by measuring the fluorescence of Abz at 420 nm with the excitation wavelength of 320 nm (Figure 2A). As shown in Figure 2B, compound **1** inhibited SIRT3 activity with an IC_50_ of 0.79 μM. 

### 2.2. Chemistry

**Scheme 1:**
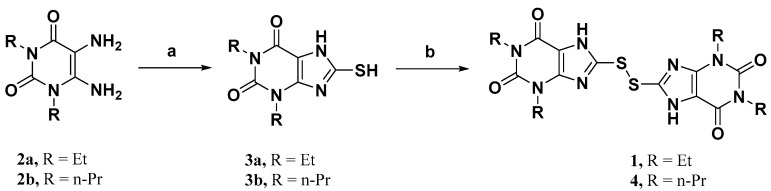
Reagents and conditions: (a) CS_2_, NaHCO_3_, EtOH:H_2_O, 65 °C, overnight; (b) I_2_, KOH, EtOH, r.t. to reflux, overnight.

**Scheme 2:**
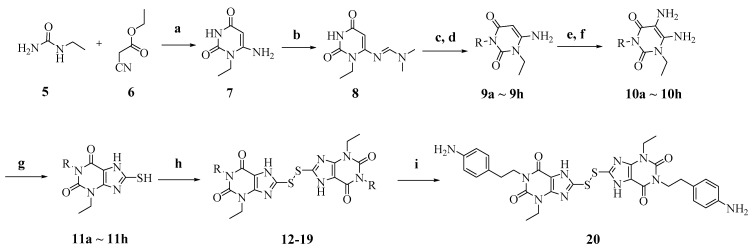
Reagents and conditions: (a) NaOEt, EtOH, reflux; (b) *N*,*N*-Dimethylformamide dimethyl acetal (DMF-DMA), MeOH, reflux; (c) R-Br, K_2_CO_3_, *N*,*N*-dimethylformamide (DMF), reflux; (d) LiOH.H_2_O, MeOH:H_2_O, 55 °C; (e) NaNO_2_, 50% HAc, 70 °C; (f) Na_2_S_2_O_4_, 14% NH_4_OH, 70 °C; (g) CS_2_, NaHCO_3_, EtOH:H_2_O, 65 °C, overnight; (h) I_2_, KOH, EtOH, r.t. to reflux, overnight; (i) For compound **17**, TFA/dichloromethane (DCM) (1:4), rt, overnight.

To study the structure–activity relationship of the inhibitor, we designed and synthesized the derivatives (Table 1: **4**, **12**–**20**) of compound **1**. The synthesis strategy is outlined in Scheme 1 and Scheme 2, which starts from commercially available diaminouracil derivatives **2a** and **2b**. Treatment of **2a** and **2b**, with carbon disulfide as one-carbon equivalent, gave access to a series of 8-thiotheophylline scaffolds **3a** and **3b**. Transformation of the key intermediates **3a** and **3b** to compounds **1** and **4** was achieved under I_2_-mediated oxidation.

Condensation of commercially available 1-ethylurea **5** with ethyl-2-cyanoacetate **6** in the presence of NaOEt afforded 6-amino-1-ethyluracil **7** (Scheme 2). Intermediate **8** was prepared by using dimethyl formamide dimethyl acetal in MeOH. This step was followed by *N*-1-alkylation of **8** with alkyl bromides and K_2_CO_3_. Deprotection of the *N*,*N*-dimethylaminomethylene moiety was efficiently achieved to afford 1,3-disubstituted-6-aminouracils **9a**–**9h**. Reaction of **9a**–**9h** with NaNO_2_ under acidic conditions yielded the desired 5-nitrosouracil derivatives, which were reduced to the 5,6-diaminouracil **10a**–**10h** with sodium dithionite. Transformation of the key intermediates **10a**–**10h** to the target compounds **12**–**19** was achieved by the same method described in scheme **1**. Deprotection of the Boc group of compound **17** under trifluoroacetic acid (TFA) gave the desired product compound **20**. All the target compounds were analyzed by HPLC (see Table S1).

### 2.3. Structure–Activity Relationship

We determined the inhibitory activities of derivatives **4, 12**–**20** for SIRT3. As shown in Table 1, compound **4** was 1.5-fold more potent than compound **1**, indicating that introduction of long-chain alkyls at the R1 position increases the activity. By comparing the activities of **1, 4,** and **12**, we can see that groups with branched chains were not tolerated well at R1, suggesting that there is limited space around the R1 position in SIRT3. Similarly, introducing (tetrahydro-2*H*-pyran-4-yl)methyl or (tetrahydrofuran-2-yl)methyl at the R1 position (compounds **13** and **14**) decreased the inhibitory activity compared to **4**. In contrast, introduction of cycloalkanes at R1 increased the hydrophobicity of the compounds (compounds **13** and **14**), leading to an enhanced activity (2.5-fold) compared to compound **12**. These indicated that hydrophobic substituent at R1 with a proper conformation may strengthen the interaction of the inhibitor with SIRT3. Inspired by this result, we tried to further improve the activity by introducing phenethyl at R1 (compound **15**). Notably, the inhibitory activity for SIRT3 was dramatically enhanced. Compounds **16**–**18**, and **20**, which have a fluoro, Boc-protected amino, methoxy, or amino at the para position of the phenethyl, respectively, exhibited lower activities. This indicates that no group was tolerant at the para position of phenethyl. In addition, replacing the phenethyl with *N*-(3-(trifluoromethyl)phenyl)propionamide (compound **19**) remarkably reduced the inhibitory activity (23-fold) compared with **15**, suggesting that a larger group at the R1 position may generate steric hindrance with SIRT3.

### 2.4. Binding Mode of the Inhibitor in SIRT3

**Figure 3:**
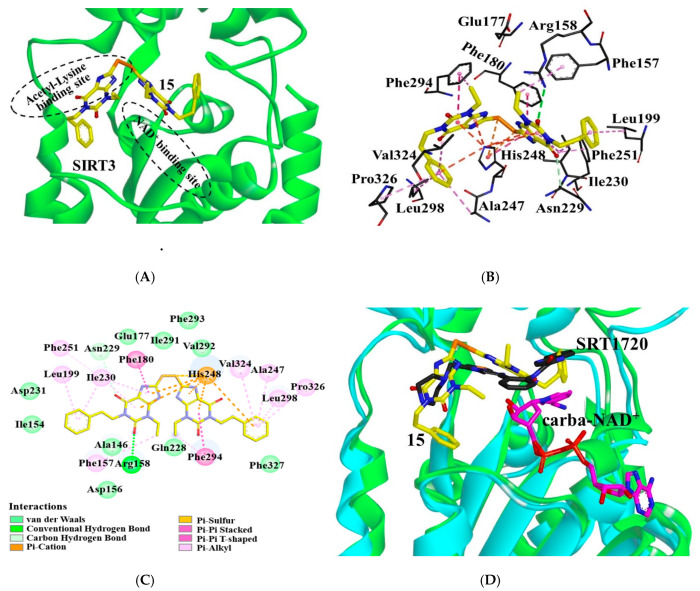
Interactions of compound **15** with SIRT3. (**A**) Binding site of **15** (yellow) in SIRT3. (**B**) **15** (yellow) interacts with the residues composed of the acetyl lysine binding site. Dotted lines in different colors represent various interactions (hydrogen-bond in green, π–π interaction in purple, π–cation, and π–sulfur interactions in orange, and CH–π interaction in pink). (**C**) Interaction details of **15** with SIRT3. (**D**) Binding modes of **15** or SRT1720 in SIRT3: SIRT3 (green) with **15** (yellow), SIRT3 (cyan)/SRT1720 (black)/carba-NAD^+^ (magenta) (pdb ID: 4BN5). Images were generated with PyMol.

Molecular docking is a powerful computational technique that has been widely used for designing bioactive compounds or studying the interactions of the ligand with its receptor. This technique is also applicable to the structure-unknown receptor or very large binding site with few hydrophobic domains such as phosphorylated tau or BACE1 [25,26,27]. To elucidate the interactions of our inhibitors, we constructed the binding mode of **15** in SIRT3 by using AutoDock4.2 (Figure 3). As known, sirtuins have a conserved catalytic core containing a Rossmann fold and a Zn^2+^-binding domain [28]. The binding sites of the acetyl substrate and NAD^+^ are located in the cleft between the two domains. According to the binding mode, the inhibitor occupied the acetyl-lysine binding site (Figure 3A). The carbonyl at the C-2 position of the inhibitor formed a hydrogen-bond with the side-chain guanidine of Arg158 (Figure 3B,C). This interacted with SIRT3 mainly through hydrophobic interactions. Specifically, the purines of the inhibitor were surrounded by hydrophobic residues: Ala146, Phe157, Phe180, Ile230, Phe251, Ile291, Val292, Phe293, and Phe294. The sulfur atom interacted with residues His248 via π–sulfur interaction. The benzene groups at both ends of the compound were inserted into the hydrophobic pockets constituted by Ile230, Leu199, Ile154 or Val324, Leu298, Ala247, Pro326, and Phe327, respectively. In addition, **15** interacted with residues Asp156, Glu177, Asp231, Gln228, and Asn229 through van der Waals interactions.

According to the binding mode, the R1 substituent of **15** is surrounded by hydrophobic residues of Leu199, Ile230, Ala247, Leu298, Val324, and Pro326. Improvement of the hydrophobicity of R1 would increase the inhibitory activity. Consistently, the IC_50_ of compounds **1**, **4**, and **15** were 0.79 μM, 0.54 μM, and 0.37 μM, respectively, indicating that replacement of ethyl at R1 with propyl or phenethyl can enhance the inhibitory activity (interactions of **1**, **4**, **12**–**14**, **16**–**20** with SIRT3 shown in Figure S1). However, introduction of groups with branched chains at R1 such as isobutyl, (tetrahydro-2*H*-pyran-4-yl)methyl or (tetrahydrofuran-2-yl)methyl (compounds **12**–**14**), appears to collide with Ala146, which decreased the activity down to 1.77 μM, 0.72 μM, and 0.69 μM, respectively. In addition, the introduction of fluoro, Boc-protected amino, methoxyl, or amino groups at the para-position to the phenethyl of **15** (compounds **16**–**18**, **20**) collided with Leu199, causing the decline of the inhibitory activity. Compound **19**, due to the introduction of a large substituent, *N*-(3-(trifluoromethyl)phenyl)propionamide at R1, producing steric hindrance with residues Ile154, Leu199, Phe302, Pro326, and Phe327, displayed a sharp decline of the inhibitory activity. The binding mode of **15** may explain the SAR of the derivatives.

### 2.5. Site-Directed Mutagenesis

**Figure 4:**
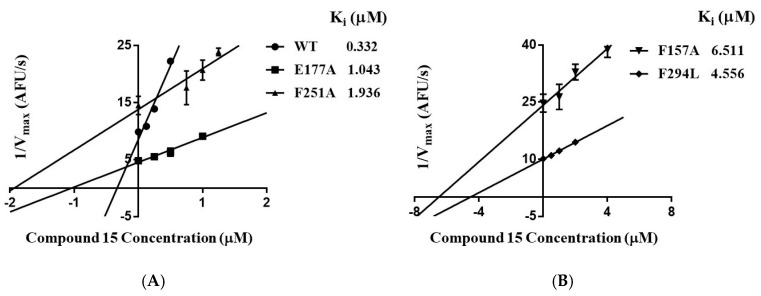
(**A**) K_i_ of compound **15** for wild-type SIRT3 and its mutants (E177A, F251A). (**B**) K_i_ of **15** for SIRT3 mutants (F157A, F294L). K_i_ is the negative of the x-intercept of the plot.

To further verify the binding mode of **15**, the residues of Phe157, Arg158, Glu177, Phe251, and Phe294 were selected for single site-directed mutation. The affinity (i.e., K_i_) of compound **15** for wild-type SIRT3 and its mutants was determined by enzyme kinetic assays. The reciprocals of the maximum reaction velocities were plotted against the concentrations of **15**. The K_i_ is the negative of the x-intercept of the plot. As shown in Figure 4, the K_i_ of the mutants were higher than those of the wild-type SIRT3. These results indicate that these residues play important roles in the interactions of **15**.

### 2.6. Mechanism of Inhibition

**Figure 5:**
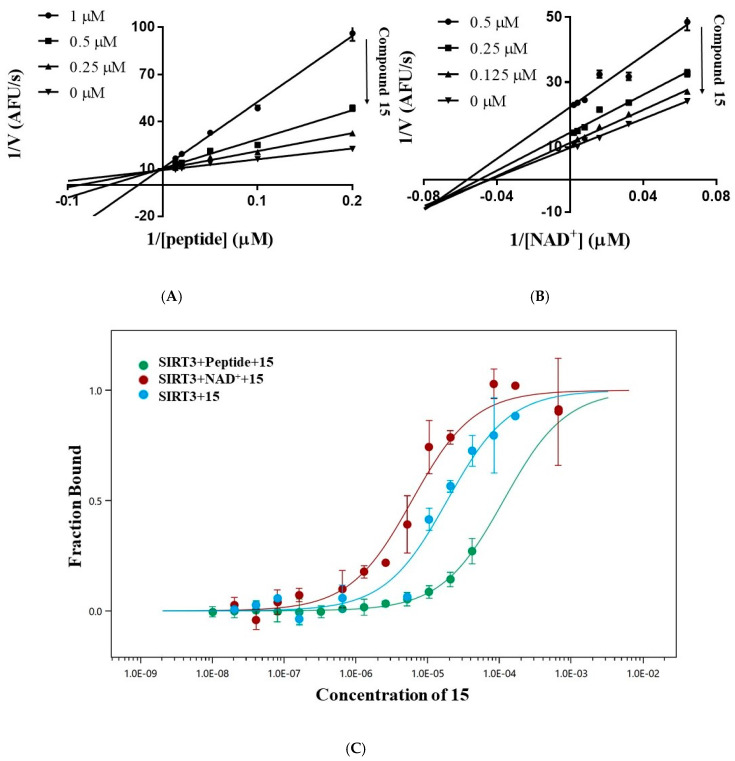
Inhibition pattern of compound **15**. (**A**) Lineweaver–Burk plot of the velocity of deacetylation versus the concentration of the acetyl peptide (5.0–100.0 μM) in absence or presence of 0.25 μM, 0.5 μM or 1.0 μM of **15** shows that **15** is a competitive inhibitor for the acetyl peptide. (**B**) Lineweaver–Burk plot of the velocity of deacetylation versus the concentration of NAD^+^ (15.625–500.0 μM) in the absence or presence of 0.125 μM, 0.25 μM, or 0.5 μM of **15** shows that **15** is a mixed-type inhibitor for NAD^+^. (**C**) Microscale thermophoresis to determine the dissociation constant K_d_ of **15** with SIRT3 in the absence or presence of 5 mM NAD^+^ or 50 μM acetyl peptide.

To identify the mechanism of the inhibition, the inhibition pattern of **15** was determined by enzyme kinetic analysis. The double-reciprocal plot of the initial reaction velocity (V) versus the concentration of the acetyl peptide or NAD^+^ shows that **15** is a competitive inhibitor for the acetyl peptide and a mixed-type inhibitor for NAD^+^ (Figure 5A,B). Increasing the concentration of **15** reduced V_max_ and K_m_ for NAD^+^, which suggested that NAD^+^ can promote the binding of **15** to SIRT3. To confirm this result, we measured the binding affinity of **15** with SIRT3 by microscale thermophoresis. As shown in Figure 5C, compound **15** exhibited a moderate affinity with K_d_ of 19.9 ± 5.3 μM for SIRT3. Adding 50 μM of the acetyl peptide decreased the affinity of **15** to SIRT3 with K_d_ > 110 μM, while the addition of 5 mM of NAD^+^ increased the affinity of **15** to SIRT3 with K_d_ of 6.0 ± 1.2 μM. Both enzyme kinetic analysis and MST experiments demonstrated that compound **15** competes with the acetyl peptide for binding to SIRT3, whereas NAD^+^ enhances the interaction of **15** in SIRT3.

To figure out the mechanism of the inhibition, we compared the binding modes of **15** and another SIRT3 inhibitor SRT1720. As shown by the superposition of SIRT3/**15** structure with that of the SIRT3/SRT1720/carba-NAD^+^ complex (pdb ID: 4BN5) [29], several similarities could be observed between the binding modes of two inhibitors (Figure 3D). Both inhibitors occupied the acetyl-lysine binding site. Additionally, **15** was found in the neighborhood of carba-NAD^+^ and may interact with the co-substrate analogue. Thus, binding of NAD^+^ can improve the affinity of **15** to SIRT3.

### 2.7. Compounds Are Potent Inhibitors of SIRT1/2/3/5.

In addition to the inhibitory activity of the compounds for SIRT3, we also measured their inhibitory activities for SIRT1, SIRT2, SIRT5, and SIRT6 (Table 1). In general, these compounds displayed notable activities for SIRT1, SIRT2, and SIRT5, but not for SIRT6. Compared with other sirtuins (SIRT1–5) that have a conserved compact acetyl substrate binding pocket, SIRT6 possesses a larger and wider hydrophobic channel [30], which may contribute to the weak affinities and activities of the inhibitors for SIRT6.

ELT-31, the most potent sirtuin inhibitor so far, was reported to inhibit SIRT1-3 with an IC_50_ of 4.3, 1.1, and 7.2 nM, which were determined by measuring the production of 2’-*O*-acetyl-ADP-ribose by mass spectrometry [22]. In our assay, ELT-31 inhibited SIRT1-3 with an IC_50_ of 0.27, 0.12, and 0.35 μM (Table 1). In contrast, the IC_50_ of our inhibitor **15** for SIRT1-3 and SIRT5 were 0.17, 1.35, 0.37, and 0.45 μM, respectively, showing that **15** was more potent than ELT-31 for SIRT1 and SIRT5, similar to ELT-31 for SIRT3.

## 3. Conclusions

Sirtuins are important deacetylases that regulate metabolism, longevity, DNA repair, apoptosis, and are involved in diseases such as inflammation, cancer, diabetes, and neurodegeneration. Sirtuin inhibitors have potential applications in the treatment of neurodegenerative diseases and various cancers. In this study, we identified novel sirtuin inhibitors based on the scaffold of 8-mercapto-3,7-dihydro-1*H*-purine-2,6-dione by a fluorescent assay. To elucidate the inhibitory mechanism, we established the binding mode of the inhibitor **15** in SIRT3 by molecular docking. The inhibitor occupied the acetyl-lysine binding site in SIRT3. It interacted with SIRT3 mainly through hydrophobic interactions and formed a hydrogen-bond with Arg158. The binding mode was verified by site-directed mutagenesis of SIRT3 and the SAR analysis of the inhibitors. We also determined the inhibition pattern of **15** by enzyme kinetic analysis, showing that **15** is a competitive inhibitor to the acetyl peptide and a mixed-type inhibitor to NAD^+^. Consistently, MST experiments proved that the acetyl peptide competes **15** for binding to SIRT3 and NAD^+^ enhances the affinity of **15** with SIRT3. Furthermore, we demonstrated that the compounds are potent SIRT1/2/3/5 pan-inhibitors. We also found that **15** is nontoxic at concentrations up to 20 μM and stable in the assays (see Figures S2 and S3). This study provides novel hits and structural information for developing potent sirtuin inhibitors. 

## 4. Experimental Section

### 4.1. Chemicals and General Methods 

Analytical thin layer chromatography (TLC) was HSGF 254 (0.15–0.2 mm thickness). Preparative thin layer chromatography (PTLC) was HSGF 254 (0.4–0.5 mm thickness). The reagents (chemicals) were purchased from commercial sources (J&K Scientific Co., Ltd., Beijing, China; TCI Development Co., Ltd., Shanghai, China; Adamas Reagent, Co., Ltd., Shanghai, China.), and used without further purification. All analytical products were characterized by their NMR and MS spectra. ^1^H and ^13^C-NMR spectra were recorded on a 400 MHz, 500 MHz, or 600 MHz instrument (Billerica, MA, USA). Chemical shifts were reported in parts per million (ppm, δ) downfield from tetramethylsilane. Proton coupling patterns were described as singlet (s), doublet (d), triplet (t), quartet (q), heptet (hept), multiplet (m), doublet of doublets (dd), and broad (br). Low-resolution mass spectra (LRMS) data were measured on Agilent 1260 Infinity II (Palo Alto, CA, USA) with electrospray ionization (ESI). High-resolution mass spectra (HRMS) were measured on Micromass Ultra Q-TOF spectrometer (Palo Alto, CA, USA).

### 4.2. Synthesis of Compounds

*1,3-Diethyl-8-mercapto-3,7-dihydro-1H-purine-2,6-dione (**3a**)* [31]. To a solution of **2a** (0.5 g, 2.52 mmol) in EtOH/H_2_O (*v*/*v* = 1:2, 12 mL) was added sodium bicarbonate (1.48 g, 17.66 mmol) and CS_2_ (2 mL, 32.79 mmol). The mixture was stirred overnight under reflux. The mixture was evaporated in vacuum, and the aqueous solution was acidified to pH 5–6 by using acetic acid. The precipitate formed was collected by filtration, washed with water, and dried under vacuum to afford compounds **3a** as a yellow solid (410 mg, 68%). ^1^H-NMR (400 MHz, dimethyl sulfoxide (DMSO)-*d*_6_) δ 13.44 (br, 1H), 13.00 (br, 1H), 4.04–3.71 (m, 4H), 1.15 (t, *J* = 7.0 Hz, 3H), 1.08 (t, *J* = 7.0 Hz, 3H). LRMS (ESI, *m*/*z*): 239.0 (M − H)^−^.

*8-Mercapto-1,3-dipropyl-3,7-dihydro-1H-purine-2,6-dione (**3b**)* [32]. Compound **3b** was prepared in the same manner as described for compound **3a**. ^1^H-NMR (400 MHz, DMSO-d_6_) δ 13.47 (s, 1H), 13.02 (s, 1H), 3.87–3.68 (m, 4H), 1.64–1.46 (m, 4H), 0.85 (dt, *J* = 14.8, 7.4 Hz, 6H). LRMS (ESI, *m*/*z*): 267.0 (M − H)^−^.

*8,8′-Disulfanediylbis(1,3-diethyl-3,7-dihydro-1H-purine-2,6-dione) (**1**).* To a solution of **3a** (80 mg, 0.33 mmol) in EtOH was added KOH (18 mg, 0.32 mmol), and the mixture was stirred for 0.5 h at room temperature. Further addition of I_2_ (88 mg, 0.35 mmol) at room temperature, and reflux overnight gave a crude product as a pale yellow powder that was filtered off, washed with H_2_O, and dried under vacuum. The crude product was purified by silica gel column (DCM/MeOH, 25/1) to afford **1** (64 mg, 80%). Mp = 252–254 °C. ^1^H-NMR (400 MHz, DMSO-*d*_6_) δ 14.28 (br, 2H), 4.03–3.88 (m, 8H), 1.19 (t, *J* = 7.0 Hz, 6H), 1.12 (t, *J* = 7.0 Hz, 6H). ^13^C-NMR (151 MHz, DMSO-*d*_6_) δ 153.49, 149.86, 147.46, 144.31, 109.95, 39.92, 39.78, 39.64, 39.50, 39.36, 39.22, 39.08, 38.22, 35.85, 13.08. MS (EI, *m*/*z*): 478 [M]^+^; HRMS (EI) cacld for C_18_H_22_O_4_N_8_S_2_ ([M]^+^): 478.1200; found: 478.1201.

*8,8’-Disulfanediylbis(1,3-dipropyl-3,7-dihydro-1H-purine-2,6-dione) (**4**).* Compound **4** was prepared in the same manner as described for compound **1** (78 mg, 84%). Mp = 241–243 °C. ^1^H-NMR (600 MHz, DMSO-*d*_6_) δ 14.25 (br, 2H), 3.92–3.87 (m, 4H), 3.86–3.82 (m, 4H), 1.66–1.59 (m, 4H), 1.59–1.52 (m, 4H), 0.86 (t, *J* = 7.4 Hz, 6H), 0.82 (t, *J* = 7.4 Hz, 6H). ^13^C-NMR (151 MHz, DMSO-*d*_6_) δ 153.66, 150.31, 147.76, 144.27, 109.72, 44.52, 42.22, 39.92, 39.78, 39.64, 39.50, 39.36, 39.22, 39.08, 20.75, 20.73, 11.13, 10.85. MS (EI, *m*/*z*): 534 [M]^+^; HRMS (EI) cacld for C_22_H_30_O_4_N_8_S_2_ ([M]^+^): 534.1826; found: 534.1819.

*6-Amino-1-ethylpyrimidine-2,4(1H,3H)-dione (****7****)* [33]. To a solution of NaOEt (15.4 g, 226 mmol) in dry EtOH was added **5** (10 g, 113 mmol) and **6** (12.8 g, 113 mmol), and the mixture was stirred under reflux for 24 h. The solvent was evaporated under vacuum, and the residue was dissolved in water (50 mL). The aqueous solution was acidified to pH 7 by using concentrated HCl. The solid formed was collected by filtration, washed with water, and dried under vacuum to afford 8 g of **7** (45% yield). ^1^H-NMR (600 MHz, DMSO-*d*_6_) δ 10.33 (s, 1H), 6.81 (s, 2H), 4.53 (s, 1H), 3.77 (q, *J* = 7.0 Hz, 2H), 1.08 (t, *J* = 7.1 Hz, 3H). MS (ESI, *m*/*z*): 156.0 (M + H)^+^.

*(E)-N’-(3-Ethyl-2,6-dioxo-1,2,3,6-tetrahydropyrimidin-4-yl)-N,N-dimethyl formimidamide (****8****)* [33]. To a solution of **7** (1 g, 6.45 mmol) in MeOH was added DMF-DMA (922 mg, 7.73 mmol), and the mixture was stirred at 50 °C for 10 h. The solvent was evaporated under vacuum, and the residue was recrystallized from EtOH to yield 1.1 g of compound **8**. ^1^H-NMR (600 MHz, DMSO-*d*_6_) δ 10.60 (s, 1H), 8.06 (s, 1H), 4.96 (d, *J* = 2.0 Hz, 1H), 3.90 (q, *J* = 6.9 Hz, 2H), 3.11 (s, 3H), 2.98 (s, 3H), 1.08 (t, *J* = 7.0 Hz, 3H). LRMS (ESI, *m*/*z*): 211.0 (M + H)^+^.

*6-Amino-1-ethyl-3-phenethylpyrimidine-2,4(1H,3H)-dione (****9a****).* To a solution of **8** (0.3 g, 1.43 mmol) in dry DMF was added (2-bromoethyl)benzene (396 mg, 2.14 mmol) and K_2_CO_3_ (296 mg, 2.14 mmol), and the mixture was stirred at 100 °C for 24 h. The reaction mixture was diluted with ethyl acetate (EA), and the organic layer was washed with water for three times, and dried over anhydrous Na_2_SO_4_, and concentrated under reduced pressure to afford the crude product for the next step without further purification.

Crude product in MeOH:H_2_O (*v*/*v* = 9:1) was added LiOH^.^H_2_O (180 mg, 4.28 mmol), and then heated to 55 °C for 12 h. The solvent was acidified to pH 7 by using concentrated HCl and evaporated under vacuum. The residue was purified by silica gel column (DCM/MeOH, 20/1) to afford **9a** (210 mg, 57% for 2 steps). ^1^H-NMR (400 MHz, DMSO-*d*_6_) δ 7.31–7.26 (m, 2H), 7.22–7.15 (m, 3H), 6.96 (s, 2H), 4.67 (s, 1H), 3.98–3.76 (m, 4H), 2.78–2.67 (m, 2H), 1.07 (t, *J* = 7.0 Hz, 3H). ^13^C-NMR (151 MHz, DMSO-*d*_6_) δ 161.07, 154.23, 151.02, 138.98, 128.59, 128.37, 126.18, 75.03, 41.09, 37.01, 33.65, 13.14. HRMS (ESI) cacld for C_14_H_16_N_3_O_2_ ([M − H]^−^): 258.1248; found: 258.1248.

*6-Amino-1-ethyl-3-isobutylpyrimidine-2,4(1H,3H)-dione (****9b****)* [33]. Compound **9b** was prepared in the same manner as described for compound **9a**. ^1^H-NMR (400 MHz, CDCl_3_) δ 5.25 (s, 2H), 4.99 (s, 1H), 3.93 (q, *J* = 7.1 Hz, 2H), 3.71 (d, *J* = 7.4 Hz, 2H), 2.17–2.05 (m, 1H), 1.28 (t, *J* = 7.1 Hz, 3H), 0.87 (d, *J* = 6.7 Hz, 6H). ^13^C-NMR (151 MHz, CDCl_3_) δ 163.48, 153.55, 151.76, 78.40, 47.96, 37.91, 27.26, 20.25, 13.44. LRMS (ESI, *m*/*z*): 212.0 (M + H)^+^.

*6-Amino-1-ethyl-3-((tetrahydro-2H-pyran-4-yl)methyl)pyrimidine-2,4(1H, 3H)-dione (****9c****).* Compound **9c** was prepared in the same manner as described for compound **9a**. ^1^H-NMR (500 MHz, DMSO-*d*_6_) δ 6.79 (s, 2H), 4.65 (s, 1H), 3.86–3.77 (m, 4H), 3.62 (d, *J* = 7.2 Hz, 2H), 3.24–3.17 (m, 2H), 1.93–1.82 (m, 1H), 1.43–1.36 (m, 2H), 1.24–1.13 (m, 2H), 1.09 (t, *J* = 7.0 Hz, 3H). ^13^C-NMR (126 MHz, DMSO-*d*_6_) δ 161.41, 154.09, 151.34, 74.98, 66.65, 44.80, 37.00, 33.61, 30.38, 13.11. HRMS (ESI) cacld for C_12_H_18_N_3_O_3_ ([M − H]^−^): 252.1354; found: 252.1355.

*6-Amino-1-ethyl-3-((tetrahydrofuran-2-yl)methyl)pyrimidine-2,4(1H,3H)-dione (****9d***). Compound **9d** was prepared in the same manner as described for compound **9a**. ^1^H-NMR (400 MHz, CDCl_3_) δ 6.54–6.16 (m, 2H), 4.86 (s, 1H), 4.42–4.32 (m, 1H), 4.22–4.00 (m, 2H), 3.92 (q, *J* = 7.2 Hz, 1H), 3.81–3.63 (m, 3H), 2.12–1.56 (m, 4H), 1.21 (t, *J* = 7.0 Hz, 3H). ^13^C-NMR (101 MHz, CDCl_3_) δ 163.65, 154.78, 151.93, 76.62, 76.55, 67.58, 44.43, 37.68, 29.17, 25.36, 12.73. HRMS (ESI) cacld for C_11_H_16_N_3_O_3_ ([M − H]^−^): 238.1197; found: 238.1191.

*3-(4-Amino-3-ethyl-2,6-dioxo-3,6-dihydropyrimidin-1(2H)-yl)-N-(3-(tri-fluoromethyl)phenyl)propanamide (****9e****).* Compound **9e** was prepared in the same manner as described for compound **9a**. ^1^H-NMR (500 MHz, DMSO-*d*_6_) δ 10.34 (s, 1H), 8.06 (d, *J* = 2.1 Hz, 1H), 7.79–7.67 (m, 1H), 7.52 (t, *J* = 8.0 Hz, 1H), 7.37 (dd, *J* = 7.7, 1.7 Hz, 1H), 6.86 (s, 2H), 4.68 (s, 1H), 4.06–4.00 (m, 2H), 3.82 (q, *J* = 7.0 Hz, 2H), 2.55 (t, *J* = 8.5, 6.4 Hz, 2H), 1.08 (t, *J* = 7.0 Hz, 3H). ^13^C-NMR (126 MHz, DMSO-*d*_6_) δ 169.68, 161.01, 154.21, 151.00, 139.86, 129.81, 129.31 (q, *J* = 31.5 Hz), 124.10 (q, *J* = 272.4 Hz), 122.66, 119.47–119.18 (m), 115.38–115.10 (m), 75.02, 37.00, 36.39, 35.07, 13.03. HRMS (ESI) cacld for C_16_H_18_F_3_N_4_O_3_ ([M + H]^+^): 371.1326; found: 371.1325.

*6-Amino-1-ethyl-3-(4-fluorophenethyl)pyrimidine-2,4(1H,3H)-dione (****9f****).* Compound **9f** was prepared in the same manner as described for compound **9a**. ^1^H-NMR (400 MHz, CDCl_3_) δ 7.26–7.21 (m, 2H), 7.12–6.86 (m, 2H), 4.97 (s, 1H), 4.35 (s, 2H), 4.15–4.02 (m, 2H), 3.91 (q, *J* = 7.2 Hz, 2H), 2.88 (q, *J* = 8.0 Hz, 2H), 1.29 (t, *J* = 7.2 Hz, 3H). ^13^C-NMR (126 MHz, DMSO-*d*_6_) δ 161.03, 160.84 (d, *J* = 241.5 Hz), 154.16, 150.98, 135.09 (d, *J* = 3.2 Hz), 130.36 (d, *J* = 7.9 Hz), 114.98 (d, *J* = 21.1 Hz), 75.03, 40.99, 36.97, 32.73, 13.07. HRMS (ESI) cacld for C_14_H_15_FN_3_O_2_ ([M − H]^−^): 276.1154; found: 276.1161.

*6-Amino-1-ethyl-3-(4-methoxyphenethyl)pyrimidine-2,4(1H,3H)-dione (****9g****).* Compound **9g** was prepared in the same manner as described for compound **9a**. ^1^H-NMR (400 MHz, DMSO-*d*_6_) δ 7.26–6.73 (m, 6H), 4.74–4.59 (m, 1H), 3.99–3.62 (m, 7H), 2.78–2.60 (m, 2H), 1.15–0.99 (m, 3H). ^13^C-NMR (151 MHz, DMSO-*d*_6_) δ 161.05, 157.71, 154.12, 150.98, 130.80, 129.79, 129.52, 113.78, 75.07, 54.95, 41.29, 36.98, 32.73, 13.12. HRMS (ESI) cacld for C_15_H_18_N_3_O_3_ ([M − H]^−^): 288.1354; found: 288.1353.

*tert-Butyl(4-(2-(4-Amino-3-ethyl-2,6-dioxo-3,6-dihydropyrimidin-1(2H)-yl) ethyl)phenyl)carbamate (****9h****).* Compound **9h** was prepared in the same manner as described for compound **9a**. ^1^H-NMR (400 MHz, DMSO-*d*_6_) δ 9.27 (s, 1H), 7.35 (d, *J* = 8.0 Hz, 2H), 7.12–7.00 (m, 2H), 6.84 (s, 2H), 4.65 (s, 1H), 3.96–3.72 (m, 4H), 2.66 (dd, *J* = 9.2, 6.5 Hz, 2H), 1.46 (s, 9H), 1.08 (t, *J* = 6.9 Hz, 3H). ^13^C-NMR (151 MHz, DMSO-*d*_6_) δ 161.01, 154.10, 152.77, 150.97, 137.67, 132.41, 128.69, 118.14, 78.83, 75.04, 41.14, 36.96, 32.91, 28.14, 13.12. HRMS (ESI) cacld for C_19_H_25_N_4_O_4_ ([M − H]^−^): 373.1881; found: 373.1879.

*5,6-Diamino-1-ethyl-3-phenethylpyrimidine-2,4(1H,3H)-dione (****10a****).* To a solution of **9a** (150 mg, 0.58 mmol) in 50% acetic acid was added NaNO_2_ (120 mg, 1.74 mmol), and the reaction mixture was stirred at 70 °C for 1.5 h. The mixture was evaporated in vacuum. The precipitate formed was collected by filtration, washed with water, and dried under vacuum to afford the crude product for the next step without further purification.

Crude product in 14% aqueous ammonium hydroxide was added to Na_2_S_2_O_4_ (302 mg, 1.74 mmol), and then heated to 70 °C for 1.5 h. The mixture was cooled to room temperature, and then evaporated under vacuum. The residue was purified by silica gel column (DCM/MeOH, 20/1) to afford **10a** (110 mg, 65% for 2 steps). ^1^H-NMR (400 MHz, CDCl_3_) δ 7.35–7.27 (m, 4H), 7.25–7.17 (m, 1H), 5.14–4.91 (m, 2H), 4.25–4.05 (m, 2H), 3.93 (q, *J* = 7.2 Hz, 2H), 3.00–2.83 (m, 2H), 2.47–2.13 (m, 3H), 1.29 (td, *J* = 7.2, 2.6 Hz, 3H). ^13^C-NMR (101 MHz, CDCl_3_) δ 161.55, 150.07, 148.80, 139.00, 129.12, 128.52, 126.43, 95.59, 42.99, 38.40, 34.29, 13.63. HRMS (ESI) cacld for C_14_H_17_N_4_O_2_ ([M − H]^−^): 273.1357; found: 273.1349.

*5,6-Diamino-1-ethyl-3-isobutylpyrimidine-2,4(1H,3H)-dione (****10b****)* [33]. Compound **10b** was prepared in the same manner as described for compound **10a**. ^1^H-NMR (400 MHz, CDCl_3_) δ 4.98 (s, 2H), 3.94 (q, *J* = 7.2 Hz, 2H), 3.76 (d, *J* = 7.4 Hz, 2H), 2.34 (s, 2H), 2.11 (dp, *J* = 13.9, 7.0 Hz, 1H), 1.30 (t, *J* = 7.2 Hz, 3H), 0.89 (d, *J* = 6.7 Hz, 6H). ^13^C-NMR (101 MHz, CDCl_3_) δ 162.01, 150.45, 148.54, 95.62, 48.41, 38.40, 27.29, 20.26, 13.64. MS (ESI, *m*/*z*): 227.0 (M + H)^+^.

*5,6-Diamino-1-ethyl-3-((tetrahydro-2H-pyran-4-yl)methyl)pyrimidine-2,4 (1H,3H)-dione (****10c***). Compound **10c** was prepared in the same manner as described for compound **10a**. ^1^H-NMR (400 MHz, CDCl_3_) δ 5.07 (s, 2H), 4.01–3.88 (m, 4H), 3.83 (d, *J* = 7.1 Hz, 2H), 3.31 (td, *J* = 11.7, 2.3 Hz, 2H), 2.32 (s, 2H), 2.01 (ddq, *J* = 14.7, 7.2, 3.6 Hz, 1H), 1.56–1.48 (m, 2H), 1.40 (dtd, *J* = 13.3, 11.6, 4.4 Hz, 2H), 1.29 (t, *J* = 7.2 Hz, 3H). ^13^C-NMR (101 MHz, CDCl_3_) δ 161.88, 150.42, 148.77, 95.40, 67.74, 46.64, 38.45, 34.20, 30.79, 13.59. HRMS (ESI) cacld for C_12_H_19_N_4_O_3_ ([M − H]^−^): 267.1463; found: 267.1456.

*5,6-Diamino-1-ethyl-3-((tetrahydrofuran-2-yl)methyl)pyrimidine-2,4(1H, 3H)-dione (****10d****).* Compound **10d** was prepared in the same manner as described for compound **10a**. ^1^H-NMR (400 MHz, CDCl_3_) δ 5.23–5.11 (m, 2H), 4.34 (dq, *J* = 10.8, 5.5 Hz, 1H), 4.18 (dd, *J* = 13.1, 8.9 Hz, 1H), 4.04–3.67 (m, 5H), 2.64 (s, 2H), 2.07–1.78 (m, 3H), 1.66 (ddt, *J* = 10.4, 8.3, 4.3 Hz, 1H), 1.34–1.21 (m, 3H). ^13^C-NMR (101 MHz, CDCl_3_) δ 161.63, 150.42, 148.82, 76.18, 67.88, 44.81, 38.42, 29.27, 25.37, 13.51. HRMS (ESI) cacld for C_11_H_17_N_4_O_3_ ([M − H]^−^): 253.1306; found: 253.1303.

*3-(4,5-Diamino-3-ethyl-2,6-dioxo-3,6-dihydropyrimidin-1(2H)-yl)-N-(3 (trifluoromethyl)phenyl)propanamide (****10e****).* Compound **10e** was prepared in the same manner as described for compound **10a**. ^1^H-NMR (400 MHz, DMSO-*d*_6_) δ 10.29 (s, 1H), 8.06 (d, *J* = 2.5 Hz, 1H), 7.79–7.69 (m, 1H), 7.53 (t, *J* = 8.0 Hz, 1H), 7.38 (d, *J* = 7.8 Hz, 1H), 6.23 (s, 2H), 4.10 (t, *J* = 7.4 Hz, 2H), 3.88 (q, *J* = 7.0 Hz, 2H), 2.93 (s, 2H), 2.57 (t, *J* = 7.5 Hz, 2H), 1.09 (t, *J* = 6.9 Hz, 3H). ^13^C-NMR (101 MHz, DMSO-*d*_6_) δ 170.09, 158.99, 149.58, 144.58, 140.28, 130.27, 129.78 (q, *J* = 31.5 Hz), 124.55 (q, *J* = 273.1 Hz), 123.12, 119.94–119.64 (m), 115.88–115.56 (m), 96.37, 37.85, 37.40, 35.49, 13.61. HRMS (ESI) cacld for C_16_H_19_F_3_N_5_O_3_ ([M + H]^+^): 386.1435; found: 386.144.

*5,6-Diamino-1-ethyl-3-(4-fluorophenethyl)pyrimidine-2,4(1H,3H)-dione (****10f****).* Compound **10f** was prepared in the same manner as described for compound **10a**. ^1^H-NMR (400 MHz, CDCl_3_) δ 7.25–7.19 (m, 2H), 7.01–6.91 (m, 2H), 5.00 (s, 2H), 4.16–4.05 (m, 2H), 3.92 (q, *J* = 7.2 Hz, 2H), 2.91–2.82 (m, 2H), 2.26 (s, 2H), 1.29 (t, *J* = 7.2 Hz, 3H). ^13^C-NMR (101 MHz, CDCl_3_) δ 161.68 (d, *J* = 243.9 Hz), 161.52, 150.06, 148.83, 134.64 (d, *J* = 3.0 Hz), 130.52 (d, *J* = 7.9 Hz), 115.26 (d, *J* = 21.1 Hz), 95.59, 42.90, 38.41, 33.43, 13.61. HRMS (ESI) cacld for C_14_H_16_FN_4_O_2_ ([M − H]^−^): 291.1263; found: 291.1259.

*5,6-Diamino-1-ethyl-3-(4-methoxyphenethyl)pyrimidine-2,4(1H,3H)-dione (****10g****).* Compound **10g** was prepared in the same manner as described for compound **10a**. ^1^H-NMR (400 MHz, DMSO-*d*_6_) δ 7.13–7.05 (m, 2H), 6.93–6.73 (m, 2H), 6.23 (s, 2H), 3.99–3.81 (m, 4H), 3.71 (s, 3H), 3.00 (s, 2H), 2.77–2.64 (m, 2H), 1.09 (t, *J* = 6.9 Hz, 3H). ^13^C-NMR (101 MHz, DMSO-*d*_6_) δ 158.62, 157.75, 149.13, 144.11, 130.79, 129.56, 113.84, 95.95, 54.99, 41.93, 37.42, 32.80, 13.32. HRMS (ESI) cacld for C_15_H_19_N_4_O_3_ ([M − H]^−^): 303.1463; found: 303.1456.

*tert-Butyl(4-(2-(4,5-Diamino-3-ethyl-2,6-dioxo-3,6-dihydropyrimidin-1 (2H)-yl)ethyl)phenyl)carbamate (****10h****).* Compound **10h** was prepared in the same manner as described for compound **10a**. ^1^H-NMR (500 MHz, CDCl_3_) δ 7.27 (d, *J* = 7.0 Hz, 2H), 7.19 (d, *J* = 8.0 Hz, 2H), 6.56 (s, 1H), 4.99 (s, 2H), 4.15−4.03 (m, 2H), 3.92 (q, *J* = 7.3 Hz, 2H), 2.90−2.77 (m, 2H), 2.19 (s, 2H), 1.50 (s, 9H), 1.29 (t, *J* = 7.2 Hz, 3H). ^13^C-NMR (126 MHz, CDCl_3_) δ 161.63, 153.04, 150.10, 148.84, 136.76, 133.73, 129.63, 118.94, 95.68, 80.51, 43.03, 38.43, 33.59, 28.49, 13.62. HRMS (ESI) cacld for C_19_H_28_N_5_O_4_ ([M + H]^+^): 390.2136; found: 390.2131.

*4-Ethyl-8-mercapto-1-phenethyl-3,7-dihydro-1H-purine-2,6-dione (****11a****).* Compound **11a** was prepared in the same manner as described for compound **3a**. ^1^H-NMR (400 MHz, DMSO-*d*_6_) δ 12.66 (s, 1H), 7.43−7.08 (m, 5H), 4.08−3.98 (m, 2H), 3.90 (q, *J* = 7.0 Hz, 2H), 2.91−2.71 (m, 2H), 1.13 (t, *J* = 7.0 Hz, 3H). ^13^C-NMR (101 MHz, DMSO-*d*_6_) δ 150.98, 149.49, 138.70, 128.66, 128.45, 126.32, 41.82, 39.03, 33.46, 13.20. HRMS (ESI) cacld for C_15_H_15_N_4_O_2_S ([M − H]^−^): 315.0921; found: 315.0919.

*3-Ethyl-1-isobutyl-8-mercapto-3,7-dihydro-1H-purine-2,6-dione (****11b****).* Compound **11b** was prepared in the same manner as described for compound **3a**. ^1^H-NMR (400 MHz, DMSO-*d*_6_) δ 13.34 (s, 1H), 3.92 (q, *J* = 7.0 Hz, 2H), 3.66 (d, *J* = 7.4 Hz, 2H), 2.00 (hept, *J* = 6.9 Hz, 1H), 1.15 (t, *J* = 7.0 Hz, 3H), 0.82 (d, *J* = 6.7 Hz, 6H). ^13^C-NMR (126 MHz, DMSO-*d*_6_) δ 151.88, 149.71, 47.31, 39.20, 26.60, 19.91, 13.10. HRMS (ESI) cacld for C_11_H_15_N_4_O_2_S ([M − H]^−^): 267.0921; found: 267.0922.

*3-Ethyl-8-mercapto-1-((tetrahydro-2H-pyran-4-yl)methyl)-3,7-dihydro-1H-purine-2,6-dione (****11c****).* Compound **11c** was prepared in the same manner as described for compound **3a**. ^1^H-NMR (400 MHz, DMSO-*d*_6_) δ 13.40 (s, 1H), 12.40 (s, 1H), 3.91 (q, *J* = 7.0 Hz, 2H), 3.85–3.65 (m, 4H), 3.26–3.11 (m, 2H), 1.97−1.90 (m, 1H), 1.50−1.34 (m, 2H), 1.22 (td, *J* = 12.2, 4.2 Hz, 2H), 1.15 (t, *J* = 7.0 Hz, 3H). ^13^C-NMR (101 MHz, DMSO-*d*_6_) δ 149.97, 66.69, 45.49, 38.82, 33.61, 30.34, 13.22. HRMS (ESI) cacld for C_13_H_17_N_4_O_2_S ([M − H]^−^): 309.1027; found: 309.1028.

*3-Ethyl-8-mercapto-1-((tetrahydrofuran-2-yl)methyl)-3,7-dihydro-1H-purine-2,6-dione (****11d****).* Compound **11d** was prepared in the same manner as described for compound **3a**. ^1^H-NMR (400 MHz, DMSO-*d*_6_) δ 13.44 (s, 1H), 13.03 (s, 1H), 4.10 (dd, *J* = 9.4, 4.0 Hz, 1H), 4.02–3.84 (m, 3H), 3.79–3.66 (m, 2H), 3.66–3.55 (m, 1H), 1.94–1.71 (m, 3H), 1.67–1.52 (m, 1H), 1.15 (t, *J* = 7.0 Hz, 3H). ^13^C-NMR (101 MHz, DMSO-*d*_6_) δ 164.13, 151.43, 149.51, 138.78, 103.77, 75.01, 66.82, 43.89, 39.57, 28.67, 24.79, 13.18. HRMS (ESI) cacld for C_12_H_15_N_4_O_3_S ([M − H]^−^): 295.087; found: 295.0871.

*3-(3-Ethyl-8-mercapto-2,6-dioxo-2,3,6,7-tetrahydro-1H-purin-1-yl)-N-(3-(trifluoromethyl)phenyl)propanamide (****11e****).* Compound **11e** was prepared in the same manner as described for compound **3a**. ^1^H-NMR (600 MHz, DMSO-*d*_6_) δ 13.46 (s, 1H), 12.99 (s, 1H), 10.31 (s, 1H), 8.05 (s, 1H), 7.72 (d, *J* = 8.2 Hz, 1H), 7.52 (t, *J* = 8.0 Hz, 1H), 7.37 (d, *J* = 7.8 Hz, 1H), 4.15 (t, *J* = 7.3 Hz, 2H), 3.90 (q, *J* = 7.1 Hz, 2H), 2.60 (t, *J* = 7.4 Hz, 2H), 1.12 (t, *J* = 7.0 Hz, 3H). ^13^C-NMR (151 MHz, DMSO-*d*_6_) δ 169.51, 149.38, 139.80, 129.87, 129.34 (q, *J* = 31.5 Hz), 124.10 (q, *J* = 272.2 Hz), 122.68, 119.61–119.26 (m), 115.54–114.81 (m), 39.31, 37.32, 34.82, 13.02. HRMS (ESI) cacld for C_17_H_17_F_3_N_5_O_3_S ([M + H]^+^): 428.0999; found: 428.1007.

*3-Ethyl-1-(4-fluorophenethyl)-8-mercapto-3,7-dihydro-1H-purine-2,6-dione (****11f***). Compound **11f** was prepared in the same manner as described for compound **3a**. ^1^H-NMR (400 MHz, DMSO-*d*_6_) δ 13.44 (s, 1H), 13.03 (s, 1H), 4.10 (dd, *J* = 9.4, 4.0 Hz, 1H), 4.02–3.84 (m, 3H), 3.79–3.66 (m, 2H), 3.66–3.55 (m, 1H), 1.94–1.71 (m, 3H), 1.67–1.52 (m, 1H), 1.15 (t, *J* = 7.0 Hz, 3H). ^13^C-NMR (101 MHz, DMSO-*d*_6_) δ 164.13, 151.43, 149.51, 138.78, 103.77, 75.01, 66.82, 43.89, 39.57, 28.67, 24.79, 13.18. HRMS (ESI) cacld for C_12_H_15_N_4_O_3_S ([M − H]^−^): 295.087; found: 295.0871.

*3-Ethyl-8-mercapto-1-(4-methoxyphenethyl)-3,7-dihydro-1H-purine-2,6-dione (****11g****).* Compound **11g** was prepared similarly as described for compound **3a**. ^1^H-NMR (400 MHz, DMSO-*d*_6_) δ 13.37 (s, 1H), 13.02 (s, 1H), 7.13–7.05 (m, 2H), 6.90–6.75 (m, 2H), 4.04–3.82 (m, 4H), 3.70 (s, 3H), 2.73 (t, *J* = 7.7 Hz, 2H), 1.13 (t, *J* = 7.0 Hz, 3H). ^13^C-NMR (101 MHz, DMSO) δ 164.16, 157.82, 151.13, 149.21, 138.88, 130.39, 129.63, 113.85, 103.79, 54.99, 42.17, 39.45, 32.45, 13.16. HRMS (ESI) cacld for C_16_H_17_N_4_O_3_S ([M − H]^−^): 345.1027; found: 345.1022.

*tert-Butyl(4-(2-(3-Ethyl-8-mercapto-2,6-dioxo-2,3,6,7-tetrahydro-1H-purin-1-yl)ethyl)phenyl)carbamate (****11h****).* Compound **11h** was prepared similarly as described for compound **3a**. ^1^H-NMR (500 MHz, DMSO-*d*_6_) δ 12.98 (s, 1H), 9.22 (s, 1H), 7.35 (d, *J* = 8.1 Hz, 2H), 7.05 (d, *J* = 8.4 Hz, 2H), 4.02–3.95 (m, 2H), 3.91 (q, *J* = 7.0 Hz, 2H), 2.77–2.67 (m, 2H), 1.46 (s, 9H), 1.14 (t, *J* = 7.0 Hz, 3H). ^13^C-NMR (126 MHz, DMSO-*d*_6_) δ 164.13, 152.74, 151.09, 149.17, 138.77, 137.77, 131.98, 128.72, 118.18, 103.75, 78.83, 41.98, 39.40, 32.60, 28.11, 13.08. HRMS (ESI) cacld for C_20_H_26_N_5_O_4_S ([M + H]^+^): 432.17; found: 432.1708.

*8,8’-Disulfanediylbis(3-ethyl-1-isobutyl-3,7-dihydro-1H-purine-2,6-dione) (**12**).* Compound **12** was prepared similarly as described for compound **1** (30 mg, 46%). Mp = 240–242 °C. ^1^H-NMR (400 MHz, DMSO-*d*_6_) δ 14.29 (br, 2H), 4.01 (q, *J* = 7.0 Hz, 4H), 3.76 (d, *J* = 7.3 Hz, 4H), 2.14–2.00 (m, 2H), 1.24–1.18 (m, 6H), 0.90–0.86 (m, 12H).^13^C-NMR (151 MHz, CDCl_3_) δ 155.27, 150.68, 148.25, 146.70, 110.06, 77.37, 77.16, 76.95, 48.76, 39.69, 27.46, 20.30, 13.54. MS (EI, *m*/*z*): 534 [M]^+^; HRMS (EI) cacld for C_22_H_30_O_4_N_8_S_2_ ([M]^+^): 534.1826; found: 534.1816.

*8,8’-Disulfanediylbis(3-ethyl-1-((tetrahydro-2H-pyran-4-yl)methyl)-3,7- dihydro-1H-purine-2,6-dione) (**13**).* Compound **13** was prepared similarly as described for compound **1** (36 mg, 50%). Mp = 229–231 °C. ^1^H-NMR (500 MHz, DMSO-*d*_6_) δ 3.98 (q, *J* = 7.0 Hz, 4H), 3.84–3.74 (m, 8H), 3.21 (td, *J* = 11.7, 2.2 Hz, 4H), 1.97–1.88 (m, 2H), 1.51–1.40 (m, 4H), 1.30–1.13 (m, 10H). ^13^C-NMR (151 MHz, CDCl_3_) δ 155.21, 150.67, 148.30, 146.95, 109.98, 77.37, 77.16, 76.95, 67.71, 47.01, 39.76, 34.34, 30.84, 13.58. MS (ESI, *m*/*z*): 617.4 [M−H]^−^; HRMS (ESI) cacld for C_26_H_33_O_6_N_8_S_2_ ([M − H]^−^): 617.197; found: 617.1958.

*8,8’-Disulfanediylbis(3-ethyl-1-((tetrahydrofuran-2-yl)methyl)-3,7-dihydro-1H-purine-2,6-dione) (**14**).* Compound **14** was prepared similarly as described for compound **1** (21 mg, 53%). Mp = 166–168 °C. ^1^H-NMR (400 MHz, DMSO-*d*_6_) δ 4.19–4.10 (m, 2H), 4.07–3.96 (m, 5H), 3.82–3.70 (m, 5H), 3.64–3.53 (m, 4H), 1.93–1.83 (m, 4H), 1.82–1.74 (m, 2H), 1.69–1.57 (m, 2H), 1.24–1.14 (m, 6H). ^13^C-NMR (151 MHz, CDCl_3_) δ 154.87, 150.62, 148.12, 146.57, 109.99, 77.37, 77.16, 76.95, 76.16, 67.95, 45.02, 39.69, 29.37, 25.35, 13.52. MS (EI, *m*/*z*): 590 [M]^+^; HRMS (EI) cacld for C_24_H_30_O_6_N_8_S_2_ ([M]^+^): 590.1724; found: 590.1733.

*8,8’-Disulfanediylbis(3-ethyl-1-phenethyl-3,7-dihydro-1H-purine-2,6-dione) (**15**).* Compound **15** was prepared similarly as described for compound **1** (45 mg, 53%). Mp = 225–227 °C. ^1^H-NMR (600 MHz, DMSO-*d*_6_) δ 14.26 (br, 2H), 7.35–7.09 (m, 10H), 4.20–3.88 (m, 8H), 2.93–2.71 (m, 4H), 1.22–1.12 (m, 6H). ^13^C-NMR (126 MHz, DMSO-*d*_6_) δ 153.55, 149.95, 147.31, 144.48, 138.53, 128.56, 128.36, 126.26, 110.00, 41.92, 38.27, 33.42, 12.97. MS (EI, *m*/*z*): 630 [M]^+^; HRMS (EI) cacld for C_30_H_30_O_4_N_8_S_2_ ([M]^+^): 630.1826; found: 630.1821.

*8,8’-Disulfanediylbis(3-ethyl-1-(4-fluorophenethyl)-3,7-dihydro-1H- purine-2,6-dione) (**16**).* Compound **16** was prepared similarly as described for compound **1** (20 mg, 33%). Mp = 216–218 °C. ^1^H-NMR (400 MHz, DMSO-*d*_6_) δ 14.28 (br, 2H), 7.28–7.19 (m, 4H), 7.15–7.06 (m, 4H), 4.12–4.05 (m, 4H), 3.98 (q, *J* = 6.9 Hz, 4H), 2.84 (t, *J* = 7.6 Hz, 4H), 1.17 (t, *J* = 7.0 Hz, 6H). ^13^C-NMR (151 MHz, DMSO-*d*_6_) δ 160.90 (d, *J* = 241.7 Hz), 153.58, 149.97, 147.45, 144.65, 134.74 (d, *J* = 3.1 Hz), 130.45 (d, *J* = 8.1 Hz), 115.08 (d, *J* = 21.1 Hz), 109.99, 41.92, 38.29, 32.59, 12.99. MS (ESI, *m*/*z*): 665.4 [M-H]^-^; HRMS (ESI) cacld for C_30_H_27_F_2_N_8_O_4_S_2_ ([M − H]^−^): 665.157; found: 665.1561.

*di-tert-Butyl(((Disulfanediylbis(3-ethyl-2,6-dioxo-2,3,6,7-tetrahydro-1H-purine-8,1-diyl))bis(ethane-2,1-diyl))bis(4,1-phenylene))dicarbamate (**17**)*. Compound **17** was prepared similarly as described for compound **1** (29 mg, 29%). Mp = 168–171 °C. ^1^H-NMR (400 MHz, DMSO-d_6_) δ 9.26 (s, 2H), 7.36 (d, J = 8.2 Hz, 4H), 7.09 (d, J = 8.2 Hz, 4H), 4.15–3.92 (m, 8H), 2.76 (t, J = 7.8 Hz, 4H), 1.46 (s, 18H), 1.18 (t, J = 7.0 Hz, 6H). ^13^C-NMR (126 MHz, DMSO-d_6_) δ 154.42, 153.25, 150.55, 147.95, 145.92, 138.27, 132.60, 129.23, 118.69, 111.41, 79.33, 55.38, 42.46, 38.81, 33.31, 28.61, 13.53. MS (ESI, *m*/*z*): 859.5 [M − H]^−^; HRMS (ESI) cacld for C_40_H_47_N_10_O_8_S_2_ ([M − H]^−^): 859.3025; found: 859.3033.

*8,8’-Disulfanediylbis(3-ethyl-1-(4-methoxyphenethyl)-3,7-dihydro-1H- purine-2,6-dione) (**18**).* Compound **18** was prepared similarly as described for compound **1** (55 mg, 66%). Mp = 216–219 °C. ^1^H-NMR (500 MHz, DMSO-*d*_6_) δ 7.13 (d, *J* = 8.6 Hz, 4H), 6.85 (d, *J* = 8.6 Hz, 4H), 4.09–3.93 (m, 8H), 3.71 (s, 6H), 2.81–2.70 (m, 4H), 1.18 (t, *J* = 7.1 Hz, 6H). ^13^C-NMR (126 MHz, DMSO-*d*_6_) δ 157.76, 154.47, 150.19, 147.60, 130.62, 129.58, 113.90, 113.82, 54.95, 42.01, 38.37, 32.72, 13.13. MS (ESI, *m*/*z*): 689.4 [M − H]^−^; HRMS (ESI) cacld for C_32_H_33_N_8_O_6_S_2_ ([M − H]^−^): 689.197; found: 689.1963.

*3,3’-(Disulfanediylbis(3-ethyl-2,6-dioxo-2,3,6,7-tetrahydro-1H-purine-8,1-diyl))bis(N-(3-(trifluoromethyl)phenyl)propanamide) (**19**).* Compound **19** was prepared in the same manner as described for compound **1** (31 mg, 40%). Mp = 174–175 °C. ^1^H-NMR (500 MHz, DMSO-d_6_) δ 10.32 (s, 2H), 8.06 (d, J = 2.0 Hz, 2H), 7.76–7.71 (m, 2H), 7.57–7.51 (m, 2H), 7.38 (d, J = 7.7 Hz, 2H), 4.23 (t, J = 7.3 Hz, 4H), 4.01 (q, J = 7.4 Hz, 4H), 2.65 (t, J = 7.3 Hz, 4H), 1.17 (t, J = 7.1 Hz, 6H). ^13^C-NMR (126 MHz, DMSO-d_6_) δ 169.57, 154.07, 150.15, 148.48, 147.55, 139.82, 129.84, 129.34 (q, J = 31.6 Hz), 124.10 (q, J = 272.2 Hz), 122.71, 119.41, 115.29, 111.16, 38.35, 37.34, 35.02, 12.98. MS (ESI, *m*/*z*): 851.4 [M − H]^−^; HRMS (ESI) cacld for C_34_H_29_F_6_N_10_O_6_S_2_ ([M − H]^−^): 851.1623; found: 851.1622.

*8,8’-Disulfanediylbis(1-(4-aminophenethyl)-3-ethyl-3,7-dihydro-1H-purine-2,6-dione) (****20****).* To a solution of **17** (20 mg, 0.33 mmol) in DCM (4 mL) was added TFA (1 mL), and the mixture was stirred for 12 h at room temperature. After completion, the reaction mixture was diluted with saturated Na_2_CO_3_ solution, extracted with DCM, dried over anhydrous Na_2_SO_4_, and concentrated under reduced pressure. The crude mixture was purified by silica gel column (DCM/MeOH, 25/1) to afford **20** (9 mg, 59%). Mp = 179–182 °C. ^1^H-NMR (400 MHz, DMSO-*d*_6_) δ 7.12 (d, *J* = 7.8 Hz, 4H), 6.92 (d, *J* = 7.7 Hz, 4H), 4.13–3.93 (m, 8H), 2.88–2.64 (m, 4H), 1.17 (t, *J* = 7.0 Hz, 6H). ^13^C-NMR (101 MHz, DMSO-*d*_6_) δ 153.64, 150.01, 147.36, 144.37, 137.83, 132.65, 129.58, 119.05, 110.12, 42.13, 38.36, 32.83, 13.06. MS (ESI, *m*/*z*): 659 [M − H]^−^; HRMS (ESI) cacld for C_30_H_31_N_10_O_4_S_2_ ([M − H]^−^): 659.1977; found: 659.1967.

### 4.3. Protein Expression and Purification 

Human SIRT3 (118-399aa) with a tobacco etch virus (TEV) cleavage site and an *N*-terminal hexahistidine (His_6_) tag was expressed in *E. coli* BL21 cells. Cells were grown in LB medium containing 100 μg/mL ampicillin at 37 °C until the A_600_ reached 0.6–0.8. A total of 100 μM isopropyl 1-β-d-galactopyranoside (IPTG) was then added to the culture and the protein expression was induced at 16 °C overnight. Cells were harvested by centrifugation and the pellet was lysed in lysis buffer (25 mM Hepes, pH 7.5, 200 mM NaCl, 5% (*v*/*v*) glycerol) and loaded with Ni–NTA. The resin was washed with 40 mM imidazole and then eluted with lysis buffer containing 250 mM imidazole. Then, the eluted protein was digested with TEV protease at 4 °C and reloaded onto the Ni–NTA column to remove the His tag. The untagged SIRT3 protein was loaded to Superdex 75 (GE Healthcare) in a buffer containing 20 mM Tris (pH 8.0), 200 mM NaCl. The SIRT3 protein was collected for further use. The mutants were prepared the same as the wild-type SIRT3.

Human SIRT1 (183-505-(GGGS)_2_-641-665) was expressed in *E. coli* BL21 cells with a small ubiquitin-related modifier (sumo)-cleavage site and an N-terminal hexahistidine (His_6_) tag [34]. Cells were grown in LB medium containing 50 μg/mL kanamycin at 37 °C until the A_600_ reached 0.6–0.8. Then, 300 μM IPTG was added to the cell culture and the protein was induced at 16 °C overnight. Cells were harvested by centrifugation and lysed in lysis buffer (20 mM Tris (pH 8.0), 500 mM NaCl, 5% (*v*/*v*) glycerol) containing 1 mM phenylmethanesulfonyl fluoride (PMSF) and loaded with Ni–NTA. The resin was washed with 20 mM imidazole and then the protein was eluted with 250 mM imidazole. The purity of the elution was confirmed by SDS−PAGE analysis, then the protein was dialyzed in a buffer containing 20 mM Tris (pH 8.0), 250 mM NaCl, and 5% (*v*/*v*) glycerol.

Human SIRT2/5/6 proteins were prepared as previously reported. [35,36,37] 

### 4.4. Molecular Modeling 

Docking of inhibitor **15** in SIRT3 was performed by using AutoDock4.2. The structures of **15** were optimized by energy minimization with a total of 200 steps until the energy difference was less than 0.1. The PDBQT format files of **15** and SIRT3 were prepared with AutoDock Tools 1.5.6. The grid box of 40 × 40 × 40 grid size with a spacing of 0.375 Å was centered at the acetyl-lysine binding pocket. The Lamarckian genetic algorithm procedure was employed while the maximum number of evaluations was set to 2,500,000, and the maximum number of generations was set to 27,000. The rate of gene mutation and crossover were set to 0.02 and 0.80, respectively. The other parameters were set as default values. The solutions were clustered into groups with root-mean-square deviation (RMSD) lower than 0.5 Å. AutoDock results were analyzed based on the lowest binding energy of the conformations [38]. The structure with a low RMSD and low energy was selected for further verification according to the structure–activity relationship.

### 4.5. Screening for Sirtuin Inhibitor 

All compounds were dissolved in 10 mM stock solution in DMSO. Reactions were performed in the assay buffer (25 mM Tris, pH 8.0, 137 mM NaCl, 2.7 mM KCl, and 1 mM MgCl_2_) containing 1.0 μM SIRT3, 10 μM Abz-GVLK(Ac)AY(NO_2_)GV-NH_2_, and 500 μM NAD^+^. The reactions were performed at 37 °C for 30 min, and then terminated by adding 10 mM nicotinamide, and developed with 0.01 mg/mL trypsin for 15 min. The fluorescence was measured using a microplate reader (SpectraMax M5, Molecular Devices, San Jose, CA, USA) with excitation at 320 nm and emission at 420 nm. All reactions were done in triplicate.

IC_50_ were determined for other sirtuins using the same assay as for SIRT3. The concentrations of SIRT1, SIRT2, SIRT5, and SIRT6 were set at 0.8 μM, 1.4 μM, 0.75 μM, and 1.3 μM to attain the same enzymatic activity as SIRT3. The concentrations of NAD^+^ were set at ~32.5-fold of K_m_ for NAD^+^ against sirtuins (SIRT1, 1.2 mM; SIRT2, 450 μM; SIRT5, 710 μM; SIRT6, 500 μM), and the concentrations of the peptide substrates were set to ~2.5-fold of K_m_ for peptide substrates against sirtuins (SIRT1, 10 μM acetyl-peptide; SIRT2, 8.5 μM acetyl-peptide; SIRT5, 2 μM succinyl-peptide; SIRT6, 50 μM acetyl-peptide).

### 4.6. Determination of the Inhibition Pattern of ***15***

To identify the mechanism of the inhibition, the inhibition pattern of **15** was determined by enzyme kinetic analysis. The assay was carried out using 0.5 μM SIRT3, 500 μM NAD^+^, and 0.01 mg/mL trypsin with variable peptide concentrations (5.0, 10.0, 20.0, 50.0, 75.0, 100.0 μM peptide sequence: Abz-GVLK(Ac)AY(NO_2_)GV-NH_2_). The concentrations of compound **15** was set at 1.0, 0.5, 0.25, and 0 μM. The double-reciprocal plot of V versus [peptide] (where V is the initial reaction velocity and [peptide] is the concentration of the acetyl peptide) was analyzed. The inhibition type of NAD^+^ by compound **15** was examined in a similar manner. The concentrations of SIRT3 and peptide were set at 0.5 μM and 30 μM, respectively. The concentrations of NAD^+^ were set at 15.625, 31.25, 62.5, 125, 250, and 500 μM, and the concentrations of compound **15** were set at 0.5, 0.25, 0.125, and 0 μM. The double-reciprocal plot of V vs. [NAD^+^] ([NAD^+^] is the concentration of NAD^+^) was analyzed.

### 4.7. Microscale Thermophoresis 

Microscale thermophoresis was performed on a Monolith NT.115 instrument (NanoTemper Technologies) according to the protocol [39]. SIRT3 was labeled by incubating with a 10-fold molar excess of Cy5 (GE Healthcare) in buffer (50 mM Tris pH 7.4, 150 mM NaCl, 10 mM MgCl_2_, 0.05% (*v*/*v*) Tween-20) for 12–16 h at 4 °C. Free dye was removed by dialysis and size-exclusion column (Superdex 75, GE Healthcare). The labeled SIRT3 was then supplemented with Bio-Beads SM-2 Adsorbent (catalog no. 152-3920, Bio-Rad Laboratories Inc., Hercules, CA, USA) to further remove the free dye. Cy5-labeled SIRT3 was mixed well with serial concentrations of compound **15** and thermophoresis was detected in the presence or absence of 50 μM acetyl peptide or 5 mM NAD^+^. Experiment settings: before MST time 3 s, MST-On time 20 s, after MST time 1 s. All measurements were done independently at least twice.

## Figures and Tables

**Table 1:**
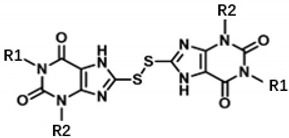
Inhibitory activities of compound **1** and its derivatives for SIRT1/2/3/5/6.

Cmpd	R1	R2			IC_50_ (μM)		
			SIRT3	SIRT1	SIRT2	SIRT5	SIRT6
**1**	–CH_2_CH_3_	–CH_2_CH_3_	0.79 ± 0.06	0.10 ± 0.01	1.17 ± 0.04	0.42 ± 0.01	116.0 ± 6.2
**4**	–CH_2_CH_2_CH_3_	–CH_2_CH_2_CH_3_	0.54 ± 0.05	0.12 ± 0.01	1.19 ± 0.06	0.39 ± 0.03	128.7 ± 16.1
**12**	–CH_2_CH(CH_3_)_2_	–CH_2_CH_3_	1.77 ± 0.05	0.43 ± 0.03	4.86 ± 0.08	1.51 ± 0.10	296.6 ± 11.3
**13**	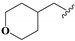	–CH_2_CH_3_	0.72 ± 0.06	0.17 ± 0.01	2.25 ± 0.12	0.50 ± 0.01	155.8 ± 6.5
**14**	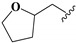	–CH_2_CH_3_	0.69 ± 0.12	0.15 ± 0.02	1.62 ± 0.05	0.54 ± 0.02	126.5 ± 10.2
**15**	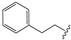	–CH_2_CH_3_	0.37 ± 0.05	0.17 ± 0.01	1.35 ± 0.05	0.45 ± 0.01	103.3 ± 6.3
**16**	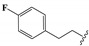	–CH_2_CH_3_	0.46 ± 0.04	0.19 ± 0.01	2.03 ± 0.04	0.84 ± 0.08	110.9 ± 2.6
**17**	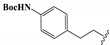	–CH_2_CH_3_	1.15 ± 0.08	0.41 ± 0.04	2.19 ± 0.08	1.00 ± 0.03	128.3 ± 4.1
**18**	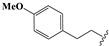	–CH_2_CH_3_	0.61 ± 0.03	0.21 ± 0.02	2.16 ± 0.08	0.61 ± 0.02	102.3 ± 9.1
**19**	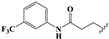	–CH_2_CH_3_	8.52 ± 0.22	1.87 ± 0.04	9.44 ± 0.19	26.12 ± 3.15	452.0 ± 9.8
**20**	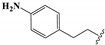	–CH_2_CH_3_	0.97 ± 0.06	0.49 ± 0.01	4.61 ± 0.13	0.92 ± 0.04	209.6 ± 5.9
**ELT-31 ^a^**	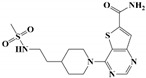		0.35 ± 0.02	0.27 ± 0.02	0.12 ± 0.01	>500	462.0 ± 14.9

^a^ the most potent sirtuin inhibitor ever reported [22].

## Supplementary Materials

The following are available online. Table S1: HPLC analyses of all the target compounds, Figure S1: 2D schematic representation of the interactions of the compounds with SIRT3, Figure S2: MTT assays to evaluate the toxicity of compounds **4** and **15**, Figure S3: The stability of the compounds.

## Abbreviations

NAD^+^	nicotinamide adenine dinucleotide
ADP	adenosine diphosphate
DMSO	dimethyl sulfoxide
DCM	dichloromethane
DMF	*N*,*N*-dimethylformamide
DMA	dimethyl acetamide
DMF-DMA	*N*,*N*-Dimethylformamide dimethyl acetal
EA	ethyl acetate
TFA	trifluoroacetic acid
TEV	tobacco etch virus
PMSF	phenylmethanesulfonyl fluoride
sumo	small ubiquitin-related modifier
RMSD	root-mean-square deviation

## References

[1] Sauve A.A. (2010). Sirtuin chemical mechanisms. Biochim. Biophys. Acta (BBA) Bioenerg..

[2] Houtkooper R.H., Pirinen E., Auwerx J. (2012). Sirtuins as regulators of metabolism and healthspan. Nat. Rev. Mol. Cell Biol..

[3] Rahman S., Islam R. (2011). Mammalian Sirt1: Insights on its biological functions. Cell Commun. Signal..

[4] Xu Z., Zhang L., Zhang W., Meng D., Zhang H., Jiang Y., Xu X., Van Meter M., Seluanov A., Gorbunova V. (2015). SIRT6 rescues the age related decline in base excision repair in a PARP1-dependent manner. Cell Cycle.

[5] Ford E., Voit R., Liszt G., Magin C., Grummt I., Guarente L. (2006). Mammalian Sir2 homolog SIRT7 is an activator of RNA polymerase I transcription. Genome Res..

[6] North B.J., Marshall B.L., Borra M.T., Denu J.M., Verdin E. (2003). The human Sir2 Ortholog, SIRT2, Is an NAD+-dependent tubulin deacetylase. Mol. Cell.

[7] Giralt A., Villarroya F. (2012). SIRT3, a pivotal actor in mitochondrial functions: Metabolism, cell death and aging. Biochem. J..

[8] Haigis M.C., Mostoslavsky R., Haigis K.M., Fahie K., Christodoulou D.C., Murphy A.J., Valenzuela D.M., Yancopoulos G.D., Karow M., Blander G. (2006). SIRT4 inhibits glutamate dehydrogenase and opposes the effects of calorie restriction in pancreatic β cells. Cell.

[9] Gertz M., Steegborn C. (2010). Function and regulation of the mitochondrial Sirtuin isoform Sirt5 in Mammalia. Biochim. Biophys. Acta (BBA)-Proteins Proteom..

[10] Wu X., Cao N., Fenech M., Wang X. (2016). Role of sirtuins in maintenance of genomic stability: Relevance to cancer and healthy aging. DNA Cell Biol..

[11] Zhou Z., Ma T., Zhu Q., Xu Y., Zha X. (2018). Recent advances in inhibitors of sirtuin1/2: An update and perspective. Futur. Med. Chem..

[12] Lara E., Mai A., Calvanese V., Altucci L., López-Nieva P., Martínez-Chantar M.L., Varela-Rey M., Rotili D., Nebbioso A., Ropero S. (2008). Salermide, a Sirtuin inhibitor with a strong cancer-specific proapoptotic effect. Oncogene.

[13] Lawson M., Uciechowska U., Schemies J., Rumpf T., Jung M., Sippl W. (2010). Inhibitors to understand molecular mechanisms of NAD+-dependent deacetylases (sirtuins). Biochim. Biophys. Acta (BBA)-Bioenerg..

[14] Heltweg B. (2006). Antitumor activity of a small-molecule inhibitor of human silent information regulator 2 enzymes. Cancer Res..

[15] E Voogd T., Vansterkenburg E.L., Wilting J., Janssen L.H. (1993). Recent research on the biological activity of suramin. Pharmacol. Rev..

[16] Lain S., Hollick J.J., Campbell J., Staples O.D., Higgins M., Aoubala M., McCarthy A., Appleyard V., Murray K.E., Baker L. (2008). Discovery, in vivo activity, and mechanism of action of a small-molecule p53 activator. Cancer Cell.

[17] Outeiro T.F., Kontopoulos E., Altmann S.M., Kufareva I., Strathearn K.E., Amore A.M., Volk C.B., Maxwell M.M., Rochet J.C., McLean P.J. (2007). Sirtuin 2 Inhibitors Rescue Alpha-Synuclein-Mediated Toxicity in Models of Parkinson’s Disease. Science.

[18] Spires-Jones T.L., Fox L.M., Rozkalne A., Pitstick R., Carlson G.A., Kazantsev A.G. (2012). Inhibition of Sirtuin 2 with Sulfobenzoic Acid Derivative AK1 is Non-Toxic and Potentially Neuroprotective in a Mouse Model of Frontotemporal Dementia. Front. Pharmacol..

[19] Cheon M.G., Kim W., Choi M., Kim J.-E. (2015). AK-1, a specific SIRT2 inhibitor, induces cell cycle arrest by downregulating Snail in HCT116 human colon carcinoma cells. Cancer Lett..

[20] Mellini P., Itoh Y., Tsumoto H., Li Y., Suzuki M., Tokuda N., Kakizawa T., Miura Y., Takeuchi J., Lahtela-Kakkonen M. (2017). Potent mechanism-based sirtuin-2-selective inhibition by an in situ-generated occupant of the substrate-binding site. Chem. Sci..

[21] Schiedel M., Robaa D., Rumpf T., Sippl W., Jung M. (2017). The current state of NAD+-Dependent histone deacetylases (sirtuins) as novel therapeutic targets. Med. Res. Rev..

[22] Disch J.S., Evindar G., Chiu C.H., Blum C.A., Dai H., Jin L., Schuman E., Lind K.E., Belyanskaya S.L., Deng J. (2013). Discovery of Thieno[3,2-d]pyrimidine-6-carboxamides as Potent Inhibitors of SIRT1, SIRT2, and SIRT3. J. Med. Chem..

[23] Sussmuth S.D., Haider S., Landwehrmeyer G.B., Farmer R., Frost C., Tripepi G., Andersen C.A., Di Bacco M., Lamanna C., Diodato E. (2015). An exploratory double-blind, randomized clinical trial with selisistat, a SirT1 inhibitor, in patients with Huntington’s disease. Br. J. Clin. Pharmacol..

[24] Roessler C., Tüting C., Meleshin M., Steegborn C., Schutkowski M. (2015). A Novel continuous assay for the deacylase sirtuin 5 and other deacetylases. J. Med. Chem..

[25] Pradeepkiran J.A., Reddy P.H., Reddy P.H., Adi P.J. (2019). Pharmacophore-based models for therapeutic drugs against phosphorylated tau in Alzheimer’s disease. Drug Discov. Today.

[26] Adi P.J., Reddy P.H. (2019). Structure based design and molecular docking studies for phosphorylated tau inhibitors in Alzheimer’s Disease. Cells.

[27] Adi P.J., Reddy A.P., Yin X., Manczak M., Reddy P.H. (2019). Protective effects of BACE1 inhibitory ligand molecules against amyloid beta-induced synaptic and mitochondrial toxicities in Alzheimer’s disease. Hum. Mol. Genet..

[28] Sanders B.D., Jackson B., Marmorstein R. (2009). Structural basis for sirtuin function: What we know and what we don’t. Biochim. et Biophys. Acta (BBA)-Bioenerg..

[29] Nguyen G.T.T., Schaefer S., Gertz M., Weyand M., Steegborn C. (2013). Structures of human sirtuin 3 complexes with ADP-ribose and with carba-NAD+and SRT1720: Binding details and inhibition mechanism. Acta Crystallogr. Sect. D Biol. Crystallogr..

[30] Parenti M.D., Bruzzone S., Nencioni A., Del Rio A. (2015). Selectivity hot-spots of sirtuin catalytic cores. Mol. BioSyst..

[31] Sultani H.N., Ghazal R.A., Hayallah A.M., Abdulrahman L.K., Abu-Hammour K., Abuhammad S., Taha M.O., Al-Eitan L. (2016). Inhibitory effects of new mercapto xanthine derivatives in human mcf7 and k562 cancer cell lines. J. Heterocycl. Chem..

[32] Famulok M., Hayallah A.M. (2007). Synthesis of New 1,3,8-Trisubstituted Purine-2,6-diones and 1,3,6-Trisubstituted Thiazolo[2,3-f]purine-2,4-diones. HETEROCYCLES.

[33] ElZein E., Kalla R.V., Li X., Perry T., Gimbel A., Zeng D., Lustig D., Leung K., Zablocki J. (2008). Discovery of a novel A2BAdenosine receptor antagonist as a clinical candidate for chronic inflammatory airway diseases. J. Med. Chem..

[34] Dai H., Case A.W., Riera T., Considine T., Lee J.E., Hamuro Y., Zhao H., Jiang Y., Sweitzer S.M., Pietrak B. (2015). Crystallographic structure of a small molecule SIRT1 activator-enzyme complex. Nat. Commun..

[35] Wu J., Zhang D., Chen L., Li J., Wang J., Ning C., Yu N., Zhao F., Chen D., Chen X. (2013). Discovery and mechanism study of SIRT1 activators that promote the deacetylation of fluorophore-labeled substrate. J. Med. Chem..

[36] Gertz M., Nguyen G.T.T., Fischer F., Suenkel B., Schlicker C., Fränzel B., Tomaschewski J., Aladini F., Becker C., Wolters D. (2012). A molecular mechanism for direct Sirtuin activation by resveratrol. PLoS ONE.

[37] Pan P.W., Feldman J.L., Devries M.K., Dong A., Edwards A.M., Denu J.M. (2011). Structure and biochemical functions of SIRT6*. J. Biol. Chem..

[38] E Lohning A., Levonis S.M., Williams-Noonan B., Schweiker S.S. (2017). A practical guide to molecular docking and homology modelling for medicinal chemists. Curr. Top. Med. Chem..

[39] Jerabek-Willemsen M., Wienken C.J., Braun D., Baaske P., Duhr S. (2011). Molecular interaction studies using microscale thermophoresis. ASSAY Drug Dev. Technol..

