# Supplementary material for: Design, Synthesis, and Biological Evaluation of 8-Mercapto-3,7-Dihydro-1H-Purine-2,6-Diones as Potent Inhibitors of SIRT1, SIRT2, SIRT3, and SIRT5
Source: Molecules. 2020 Jun 15;25(12):2755. doi: 10.3390/molecules25122755 (PMC7356367; doi:10.3390/molecules25122755)
Supplement: Supplementary file 1 [file molecules-25-02755-s001.pdf]

Supporting Information

Design, Synthesis and Biological Evaluation of 8-Mercapto-3,7-Dihydro-1*H*-Purine-2,6-Diones as Potent Inhibitors of SIRT1, SIRT2, SIRT3 and SIRT5

Content of Supporting Information

1. Copies of NMR and MS Spectra …...…………………………………………… S3

2. Table S1 HPLC analyses of all the target compounds...S54

3. Figure S1 2D schematic representation of the interactions of the compounds with SIRT3...S56

4. Figure S2 MTT assays to evaluate the toxicity of compound **4** and **15** ……..…S61

5. Figure S3 The stability of the compounds ...…………………………...…. S62

Copies of NMR and MS Spectra

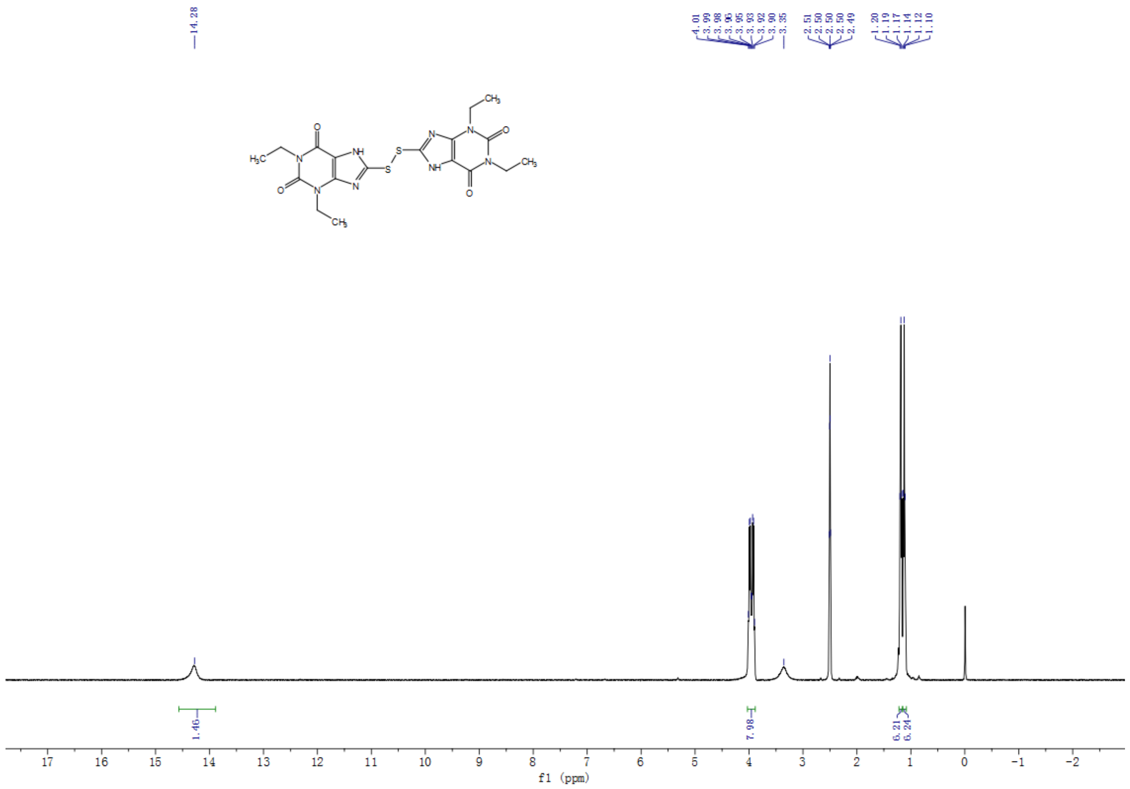

^{1}H NMR spectrum of compound **1**

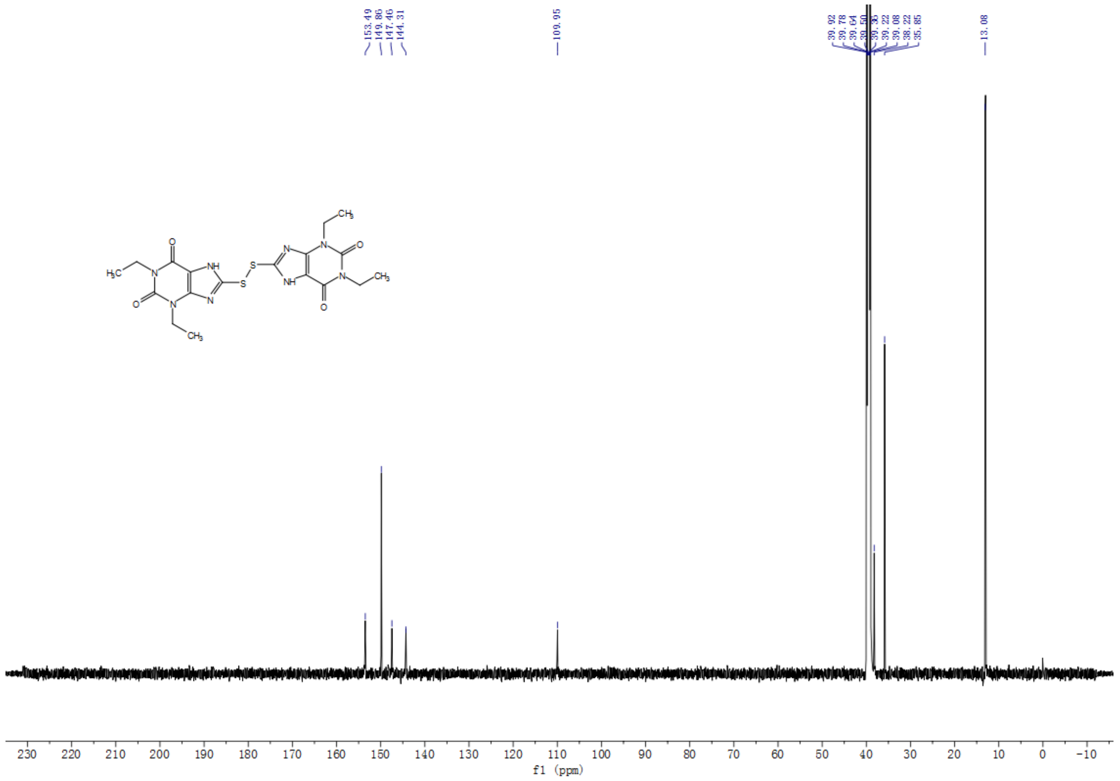

^{13}C NMR spectrum of compound **1**

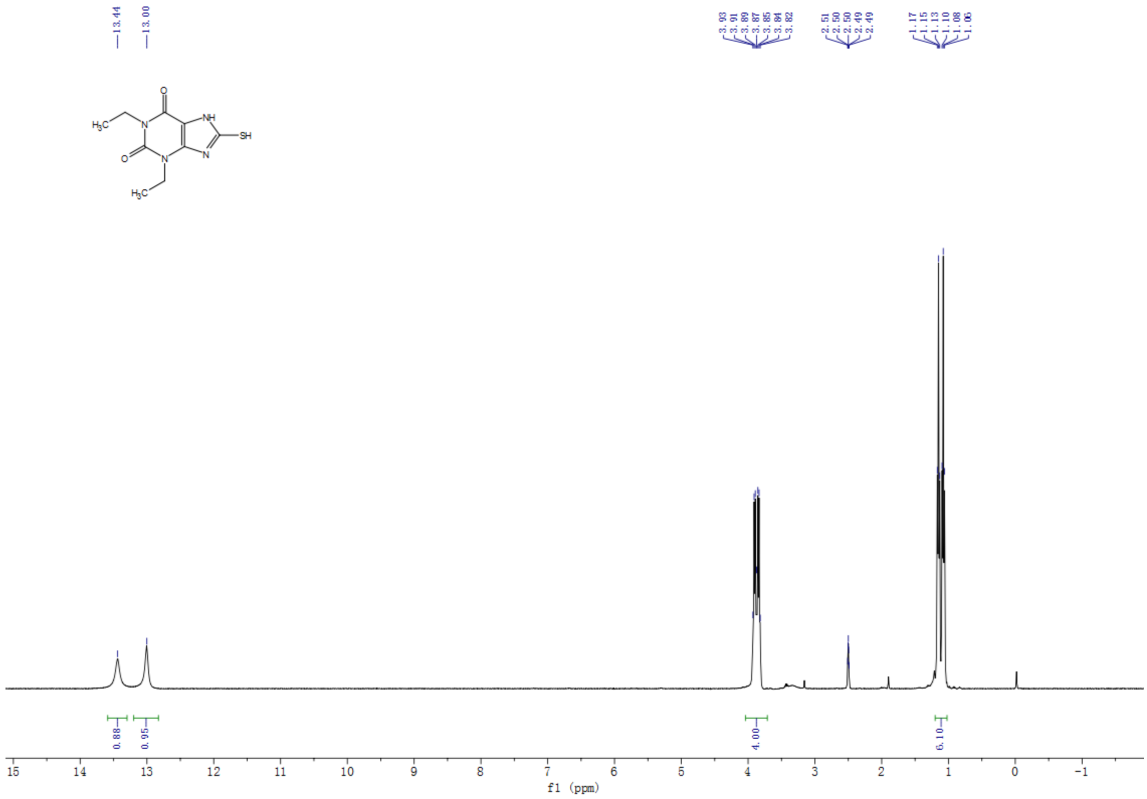

^{1}H NMR spectrum of compound **3a**

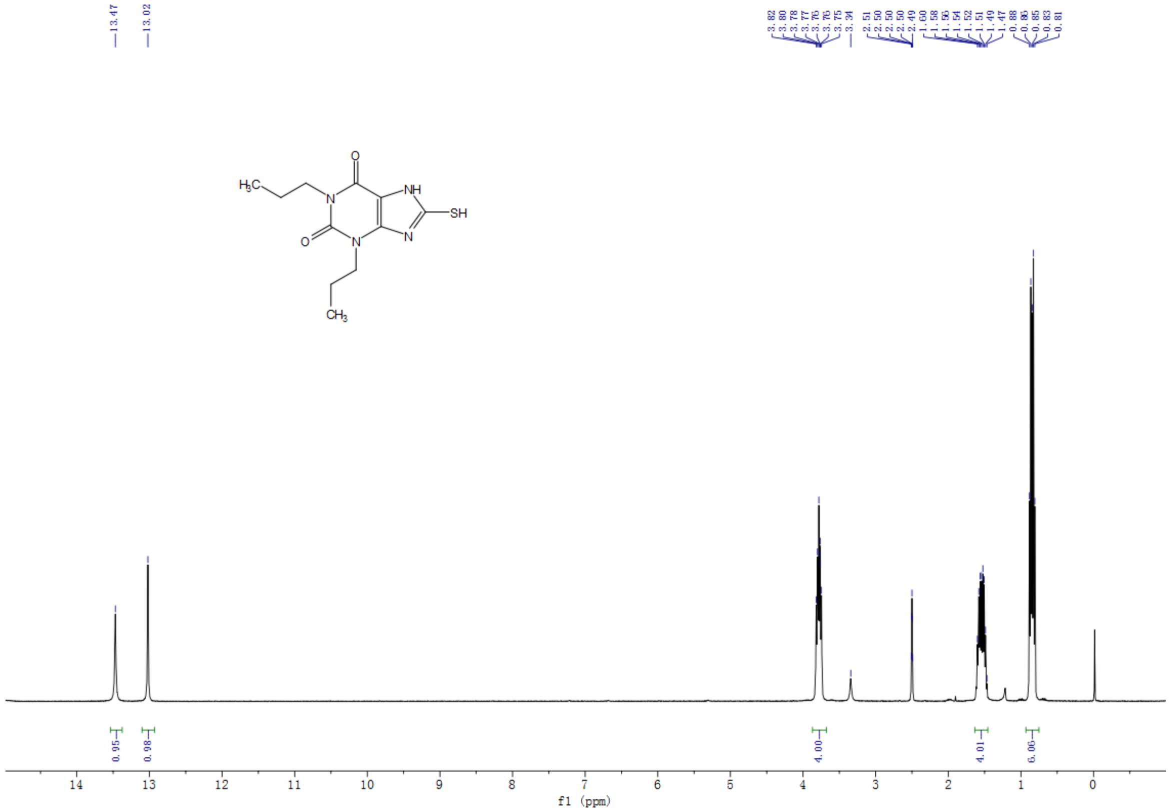

^{1}H NMR spectrum of compound **3b**

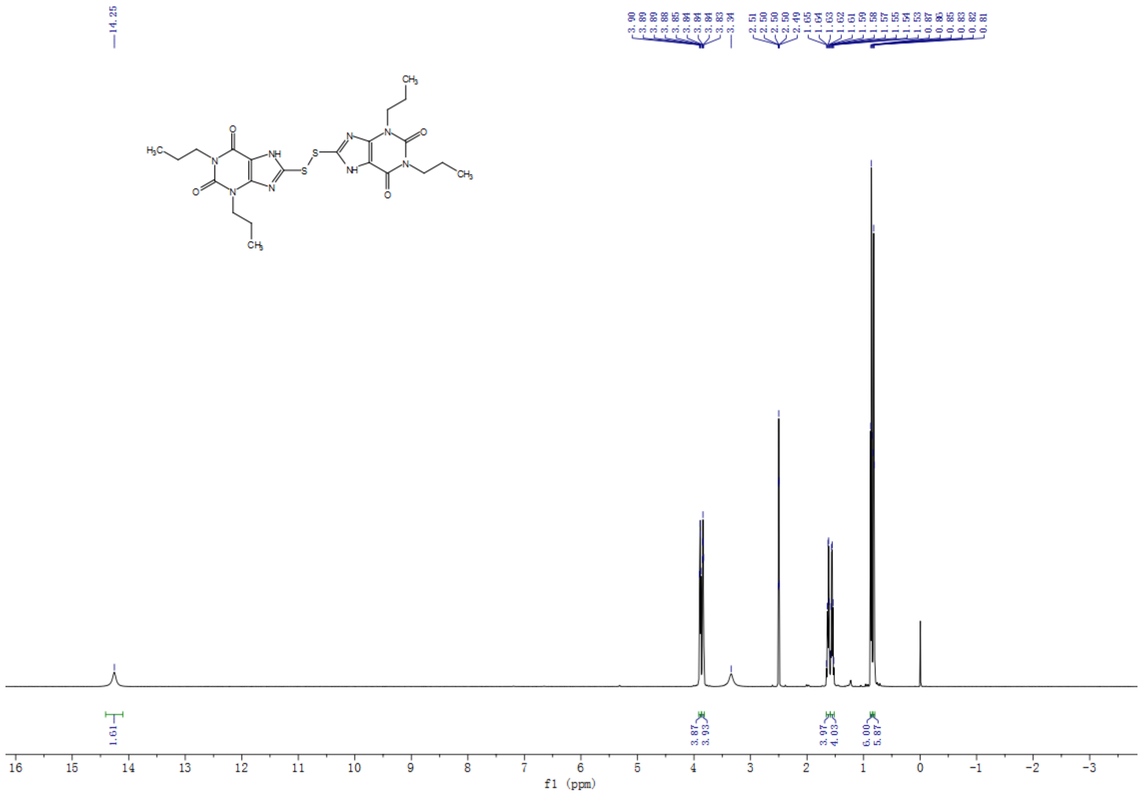

^{1}H NMR spectrum of compound **4**

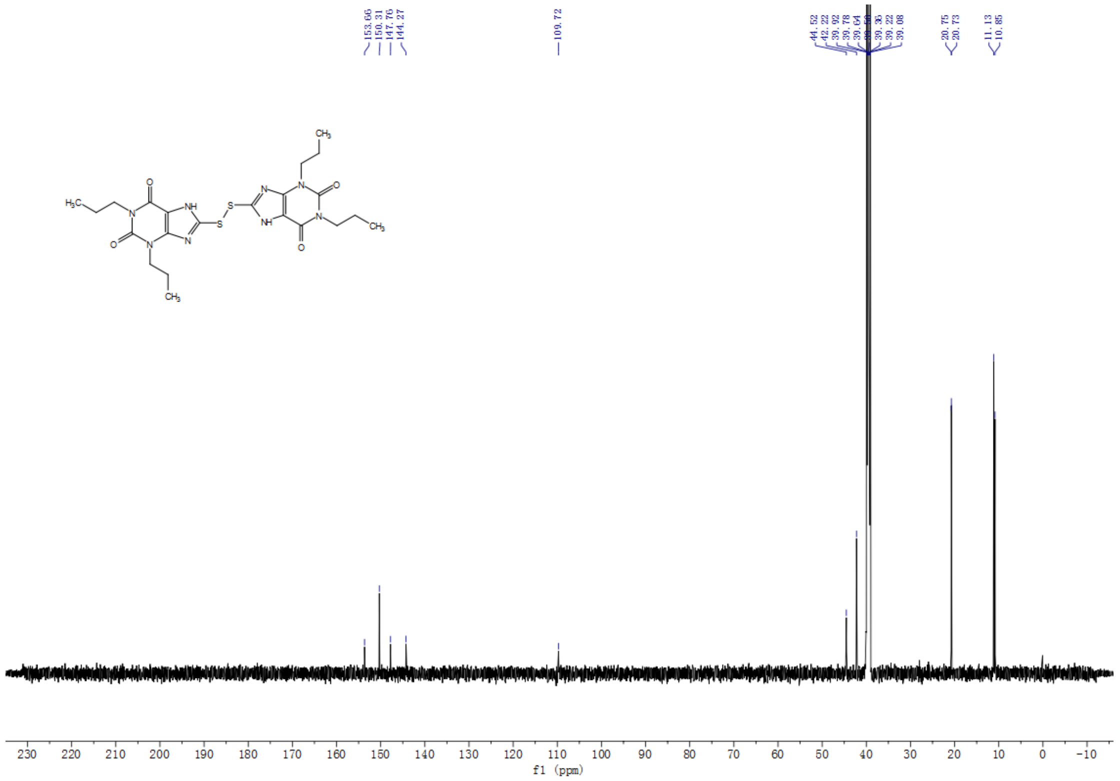

^{13}C NMR spectrum of compound **4**

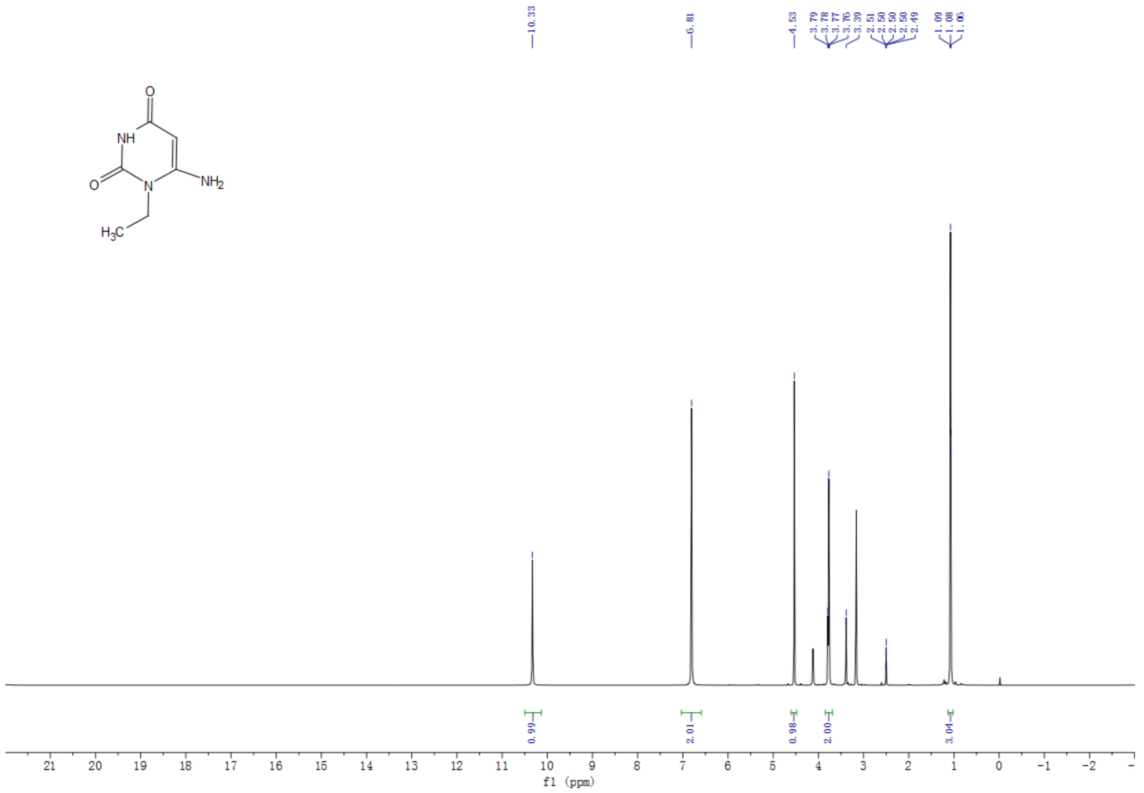

^{1}H NMR spectrum of compound **7**

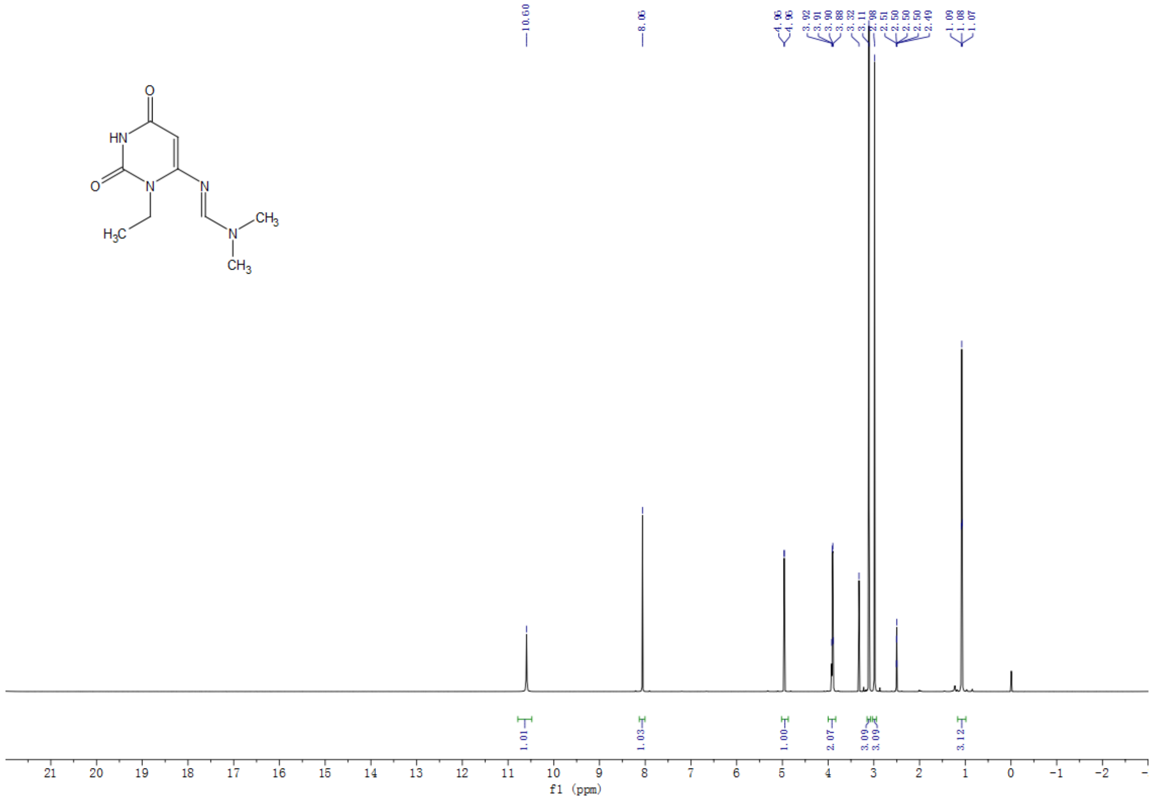

^{1}H NMR spectrum of compound **8**

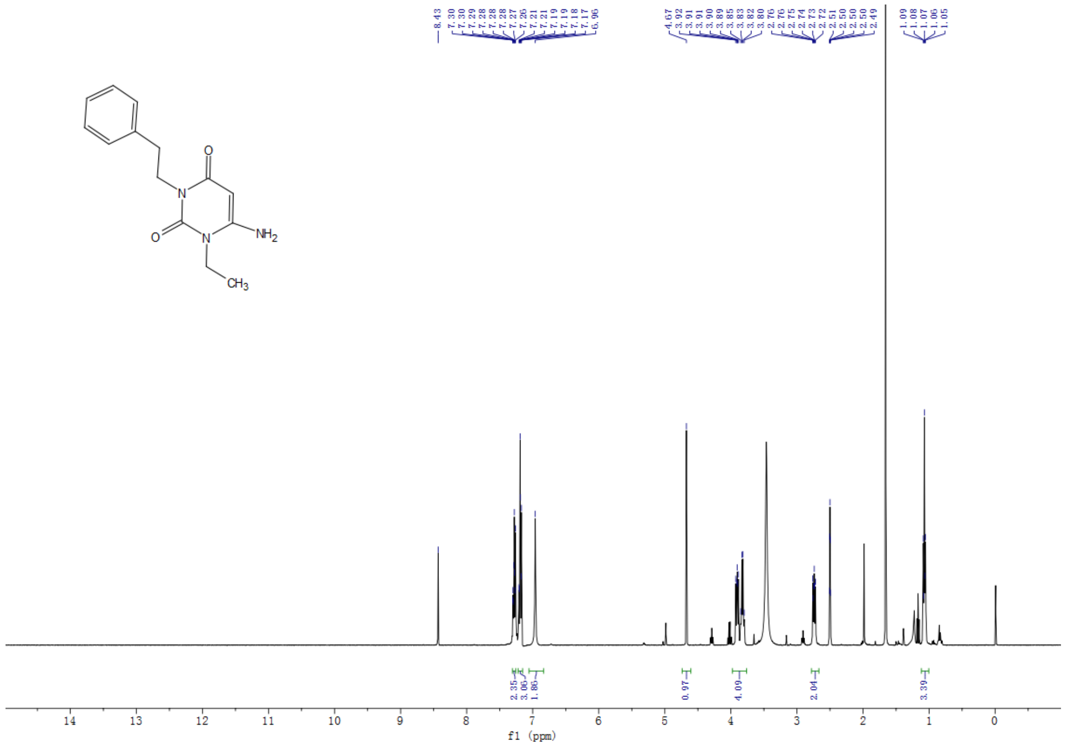

^{1}H NMR spectrum of compound **9a**

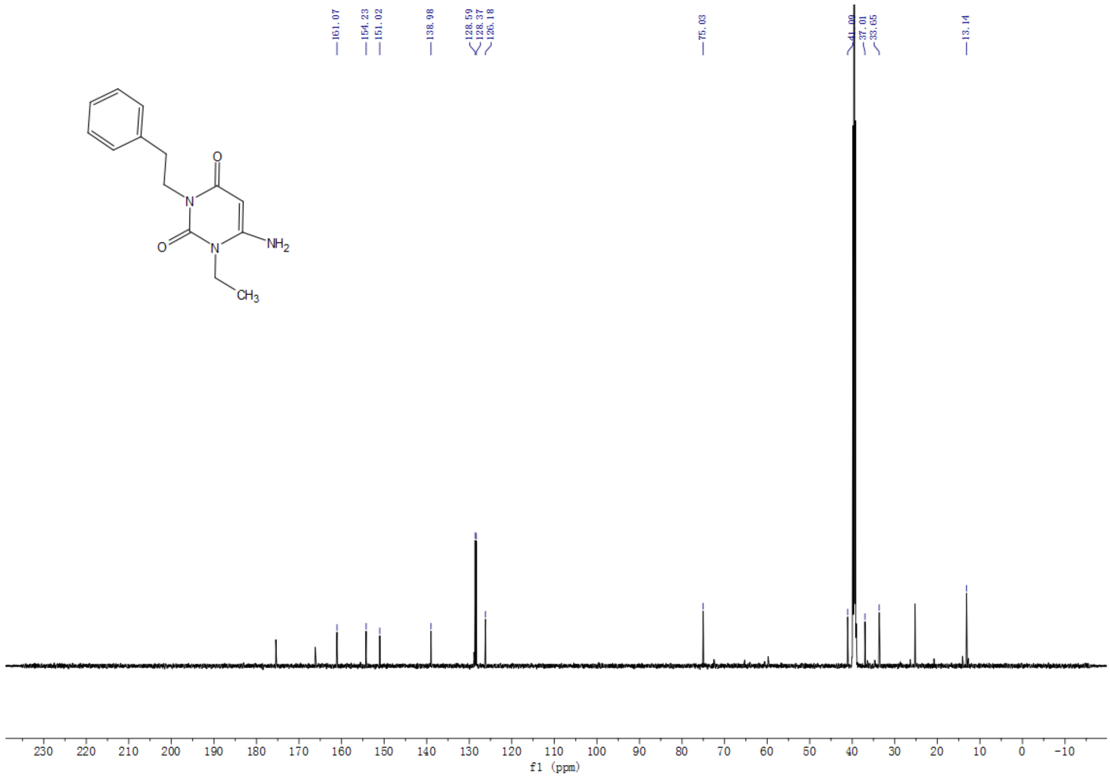

^{13}C NMR spectrum of compound **9a**

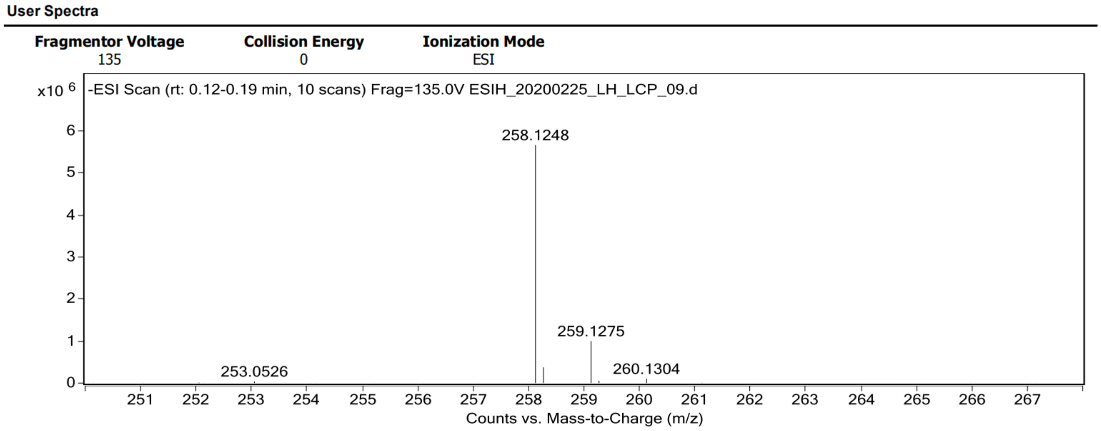

HRMS spectrum of compound **9a**

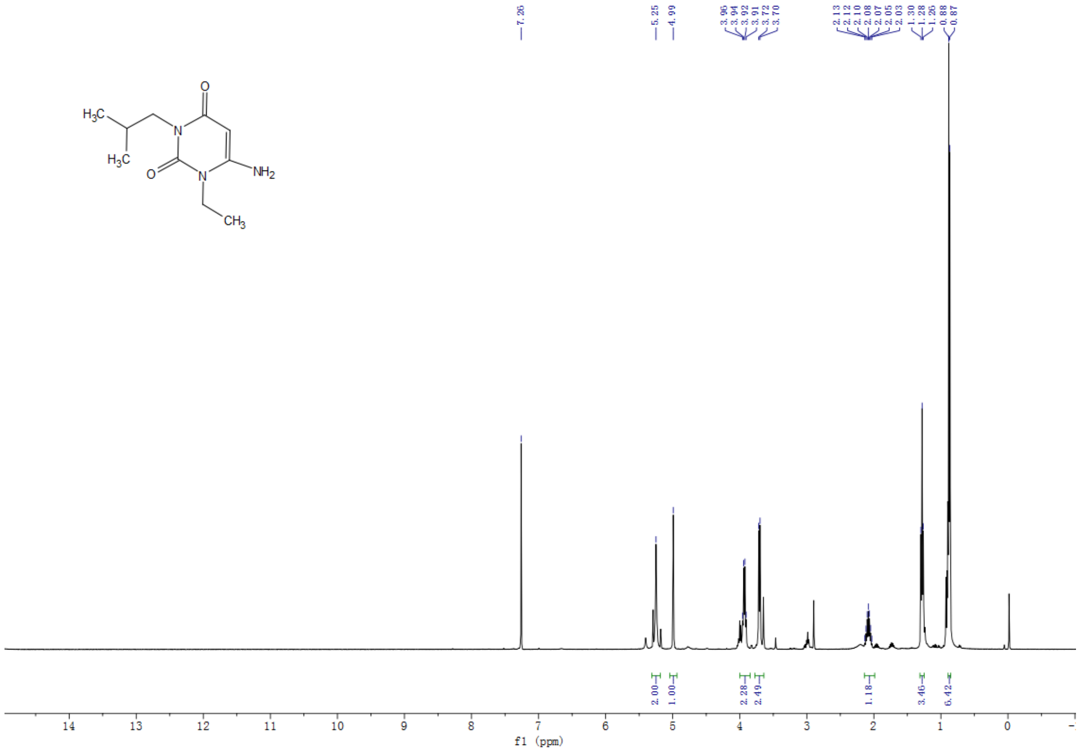

^{1}H NMR spectrum of compound **9b**

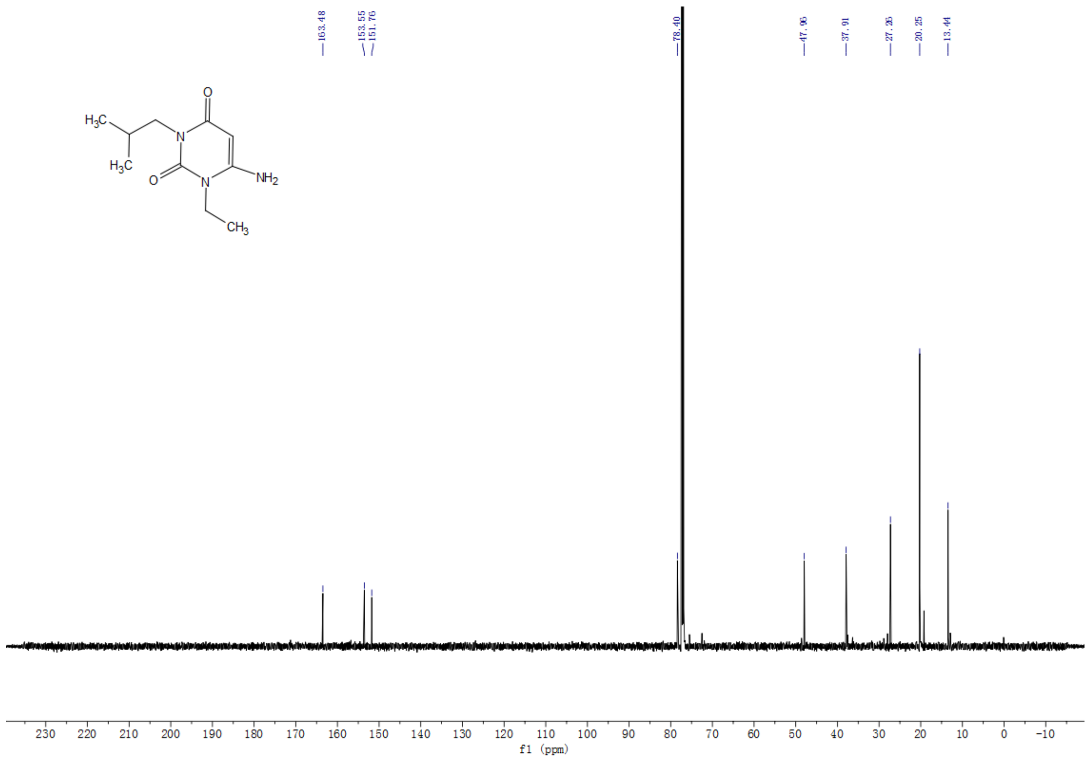

^{13}C NMR spectrum of compound **9b**

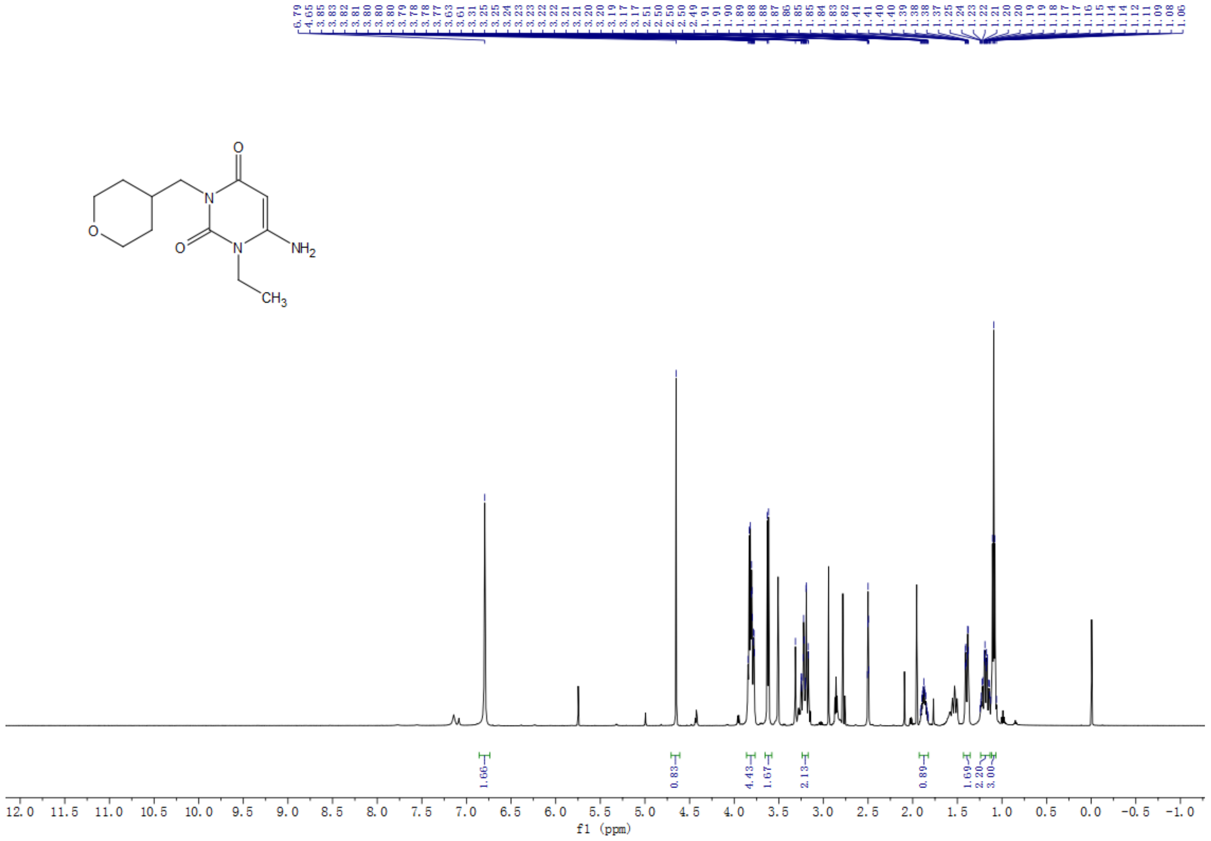

^{1}H NMR spectrum of compound **9c**

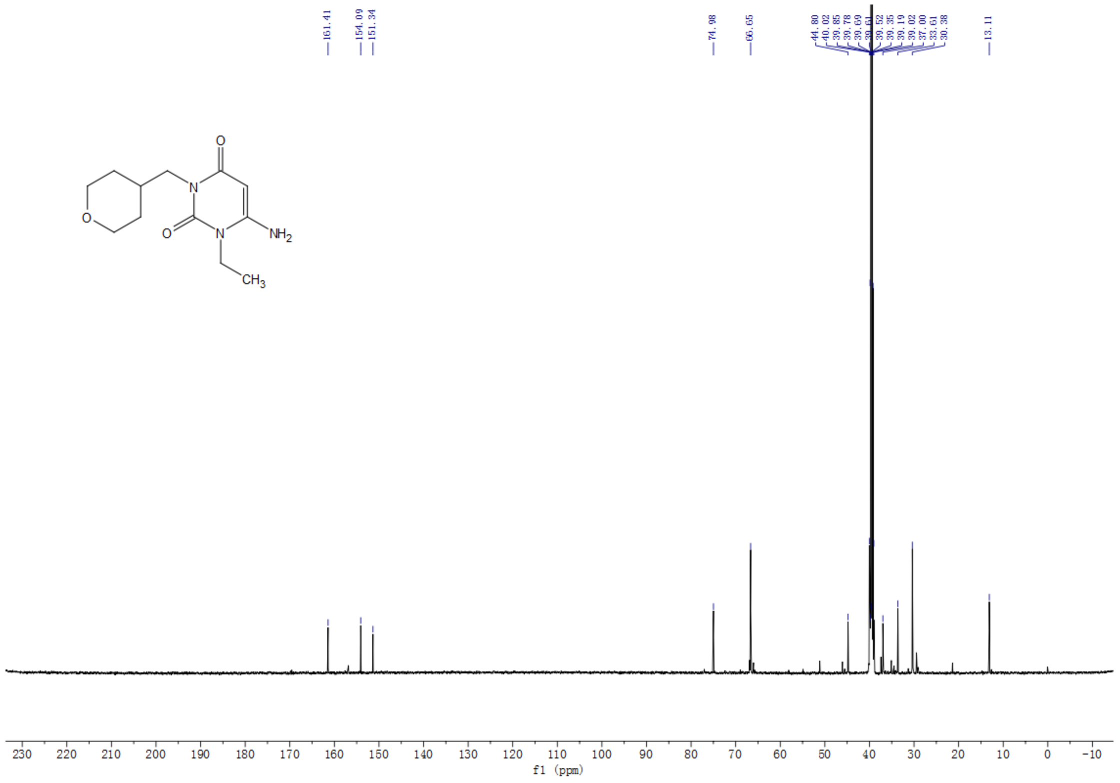

^{13}C NMR spectrum of compound **9c**

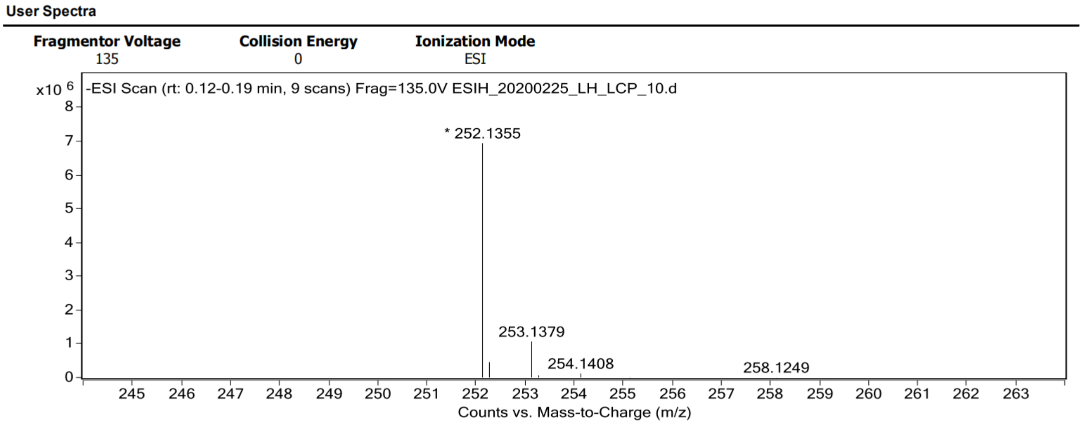

HRMS spectrum of compound **9c**

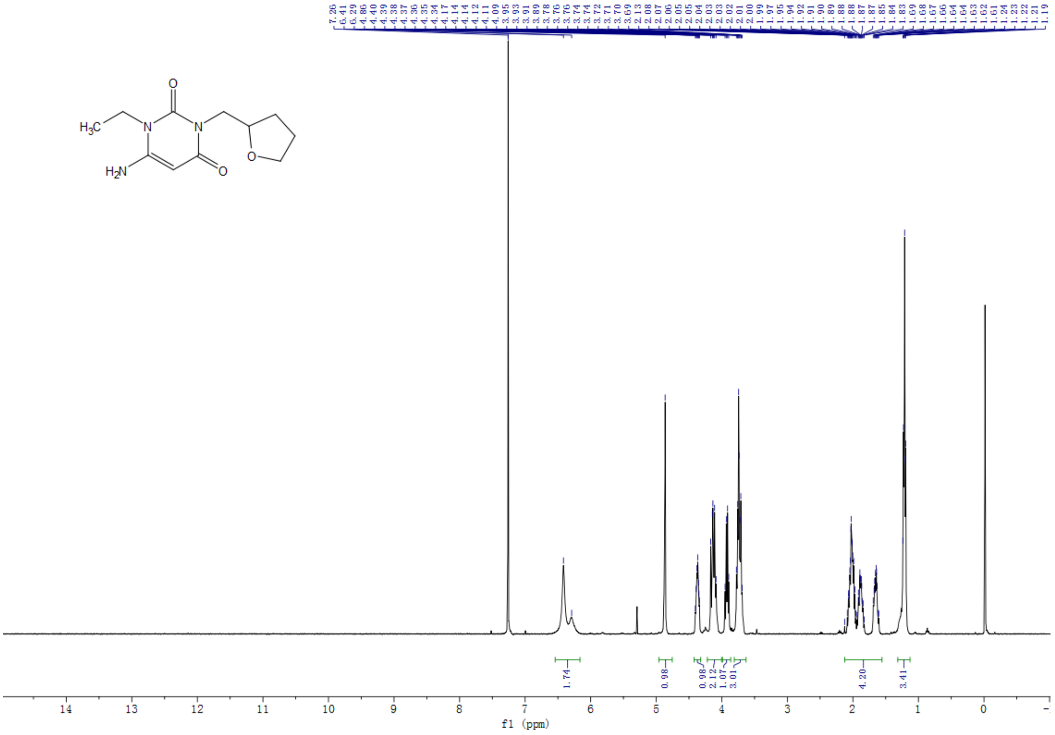

^{1}H NMR spectrum of compound **9d**

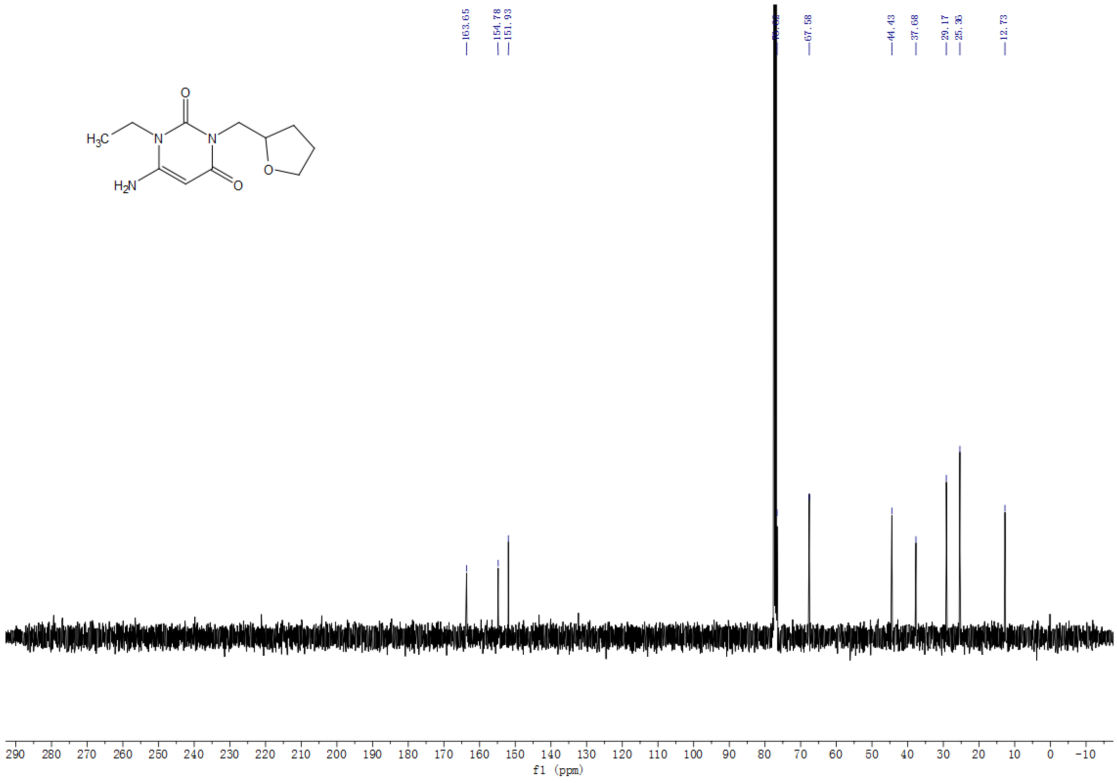

^{13}C NMR spectrum of compound **9d**

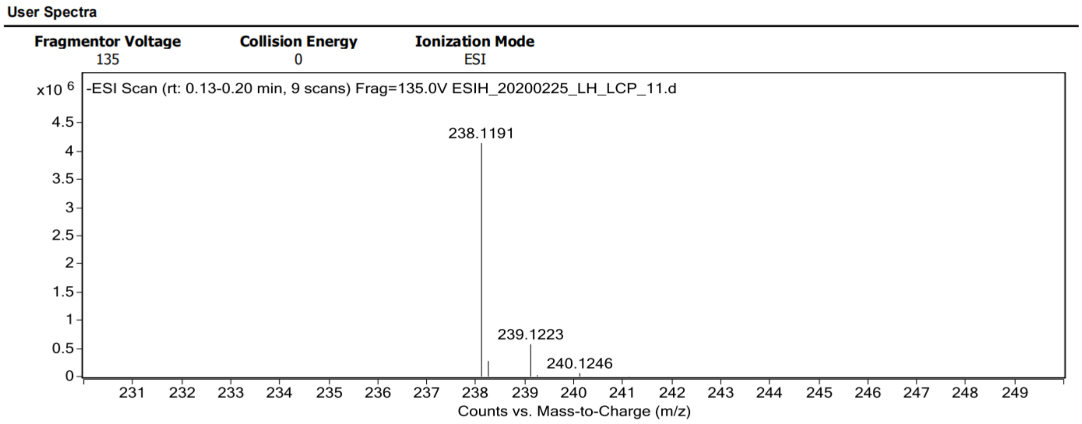

HRMS spectrum of compound **9d**

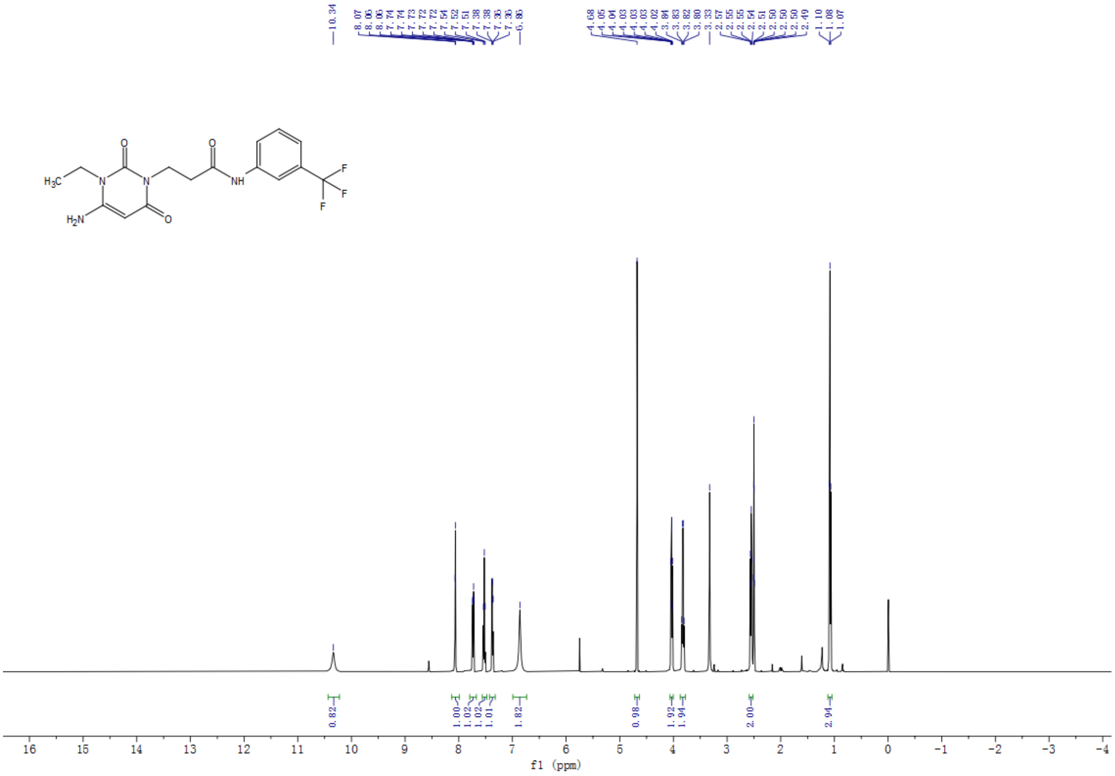

^{1}H NMR spectrum of compound **9e**

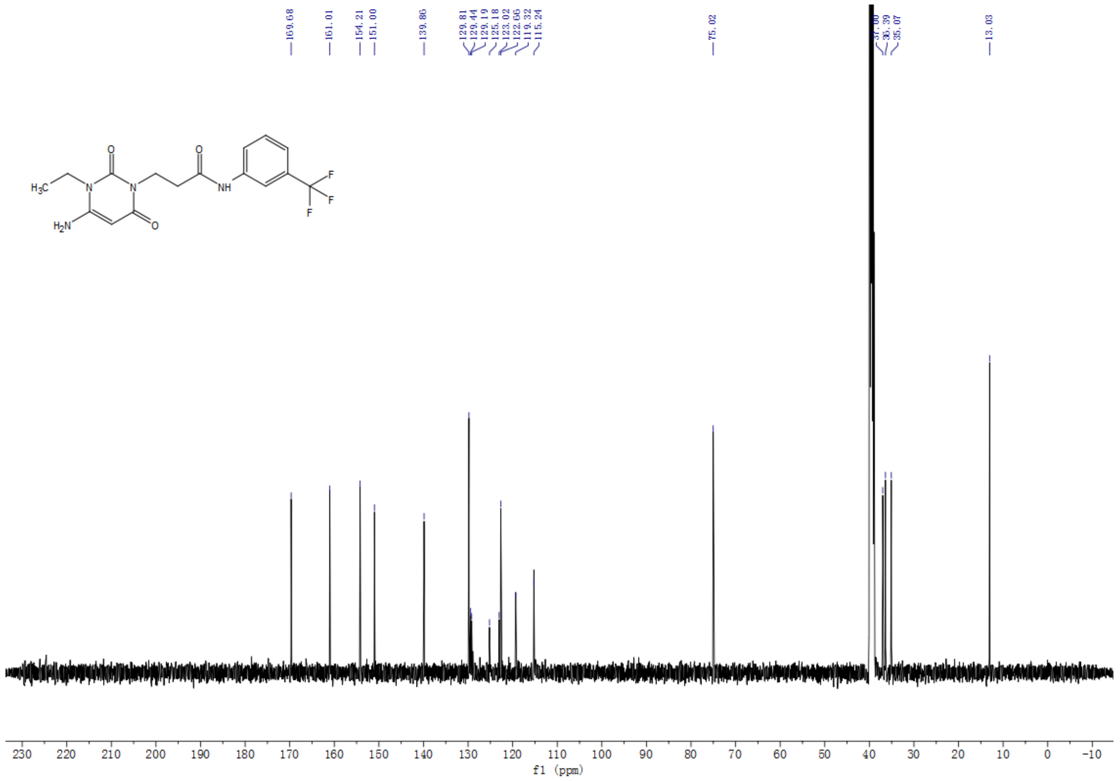

^{13}C NMR spectrum of compound **9e**

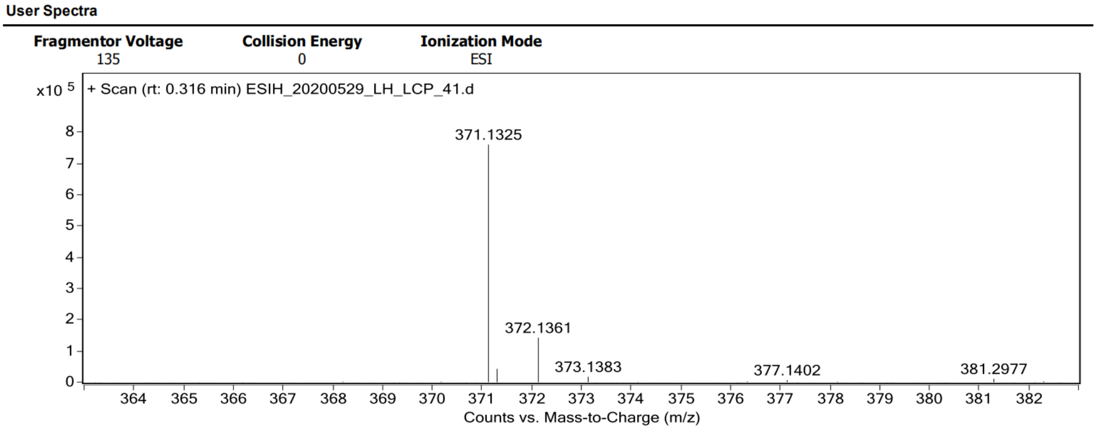

HRMS spectrum of compound **9e**

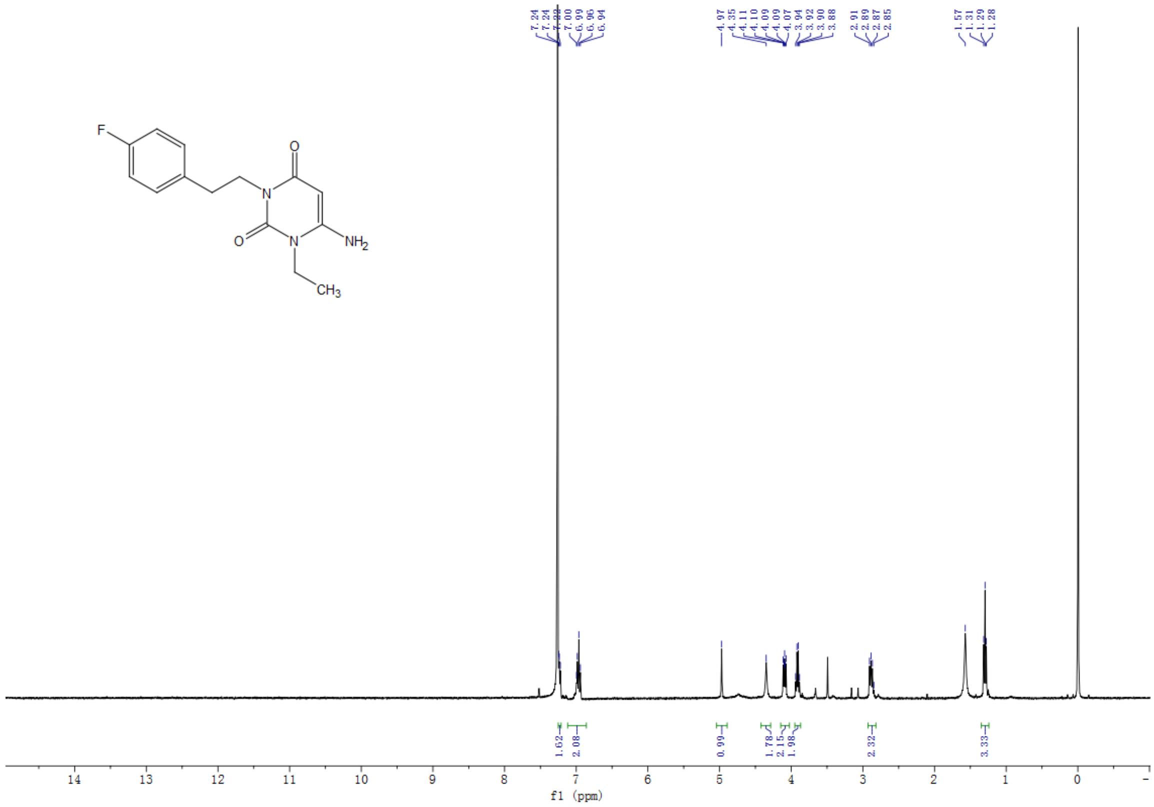

^{1}H NMR spectrum of compound **9f**

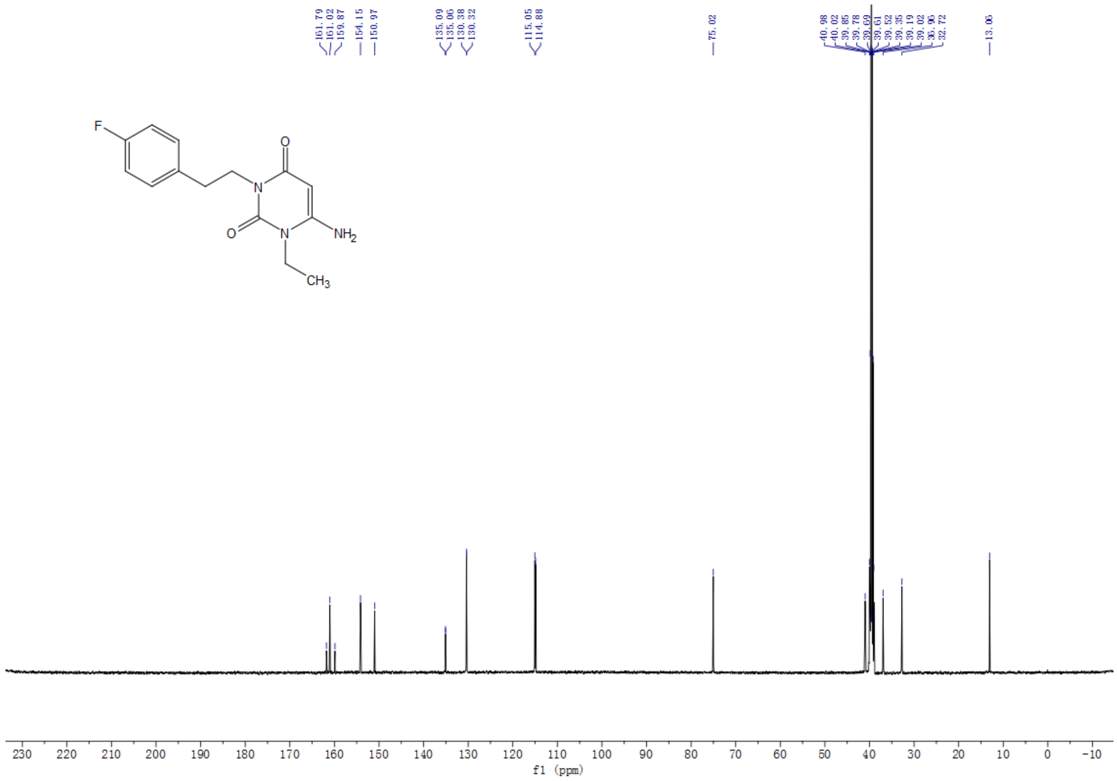

^{13}C NMR spectrum of compound **9f**

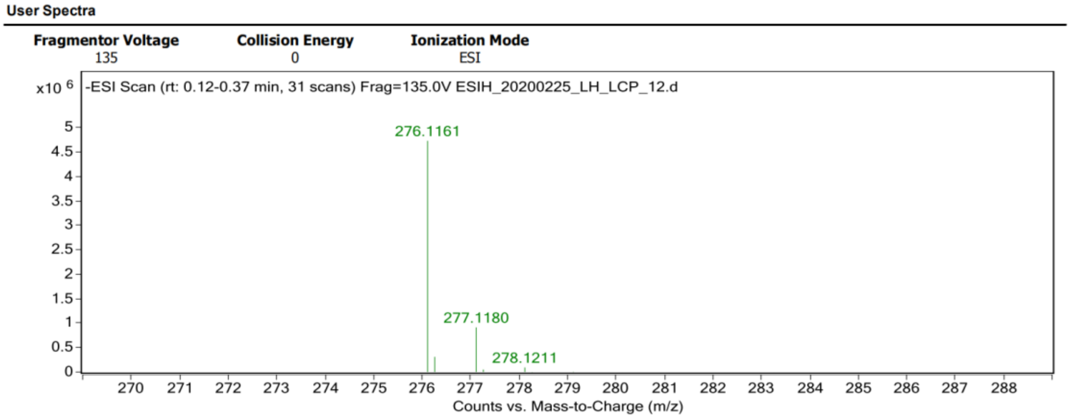

HRMS spectrum of compound **9f**

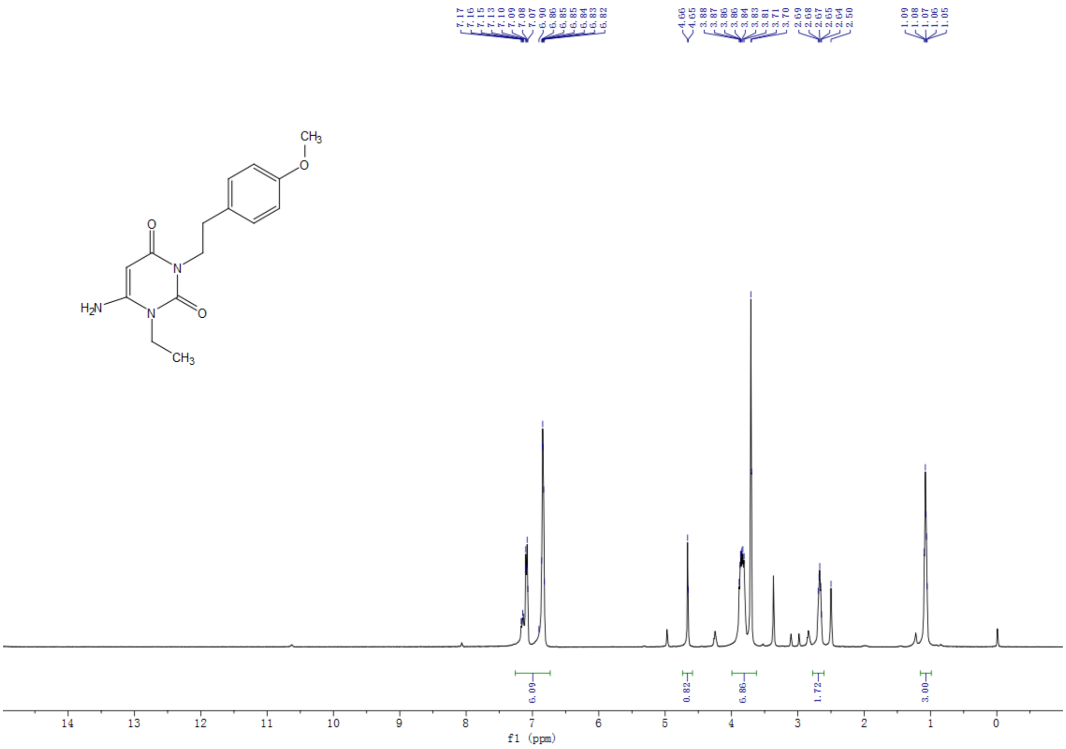

^{1}H NMR spectrum of compound **9g**

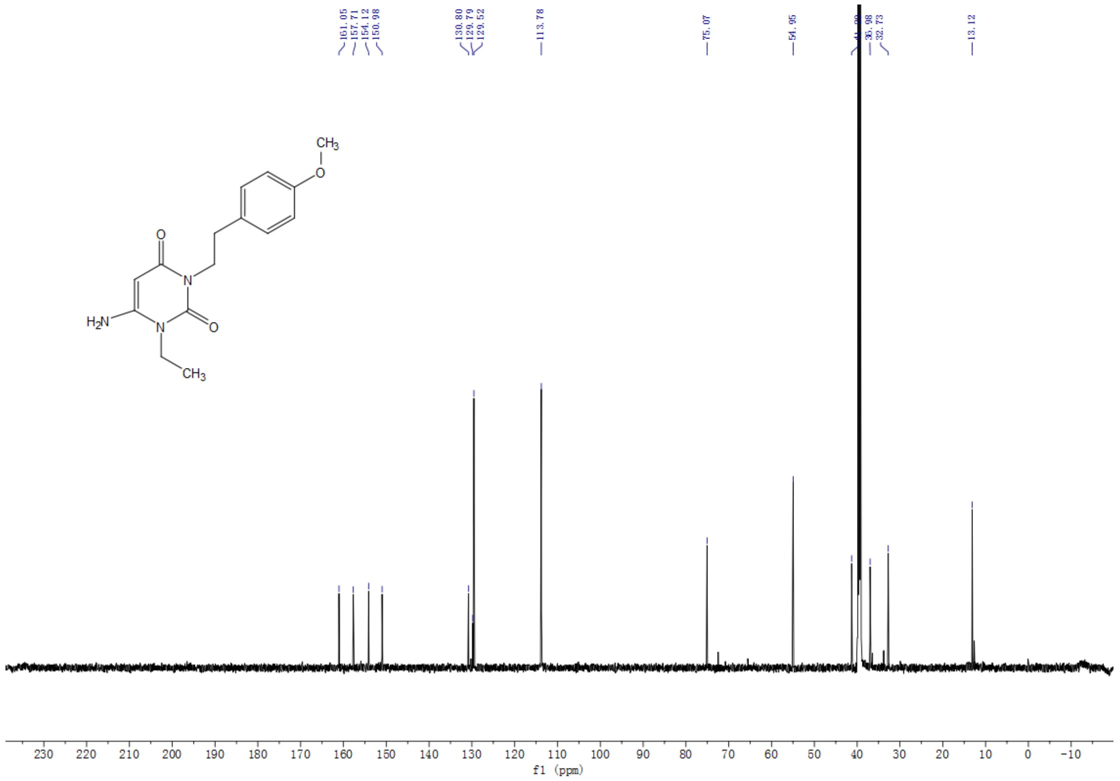

^{13}C NMR spectrum of compound **9g**

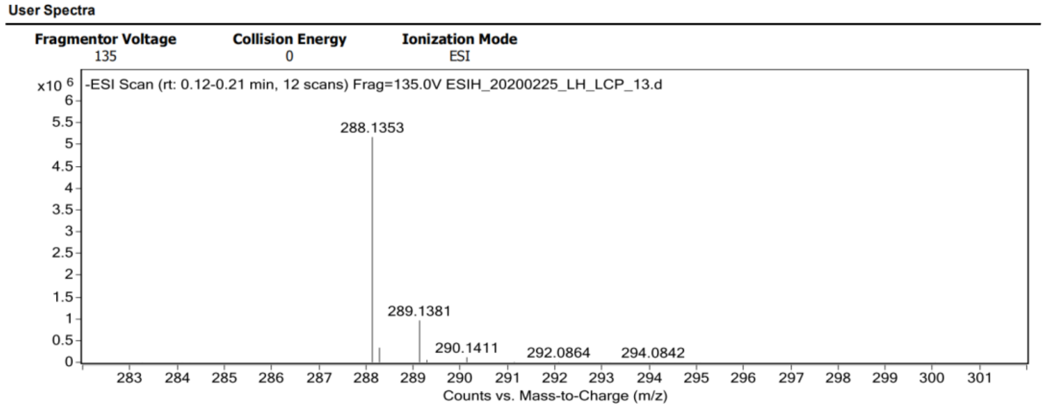

HRMS spectrum of compound **9g**

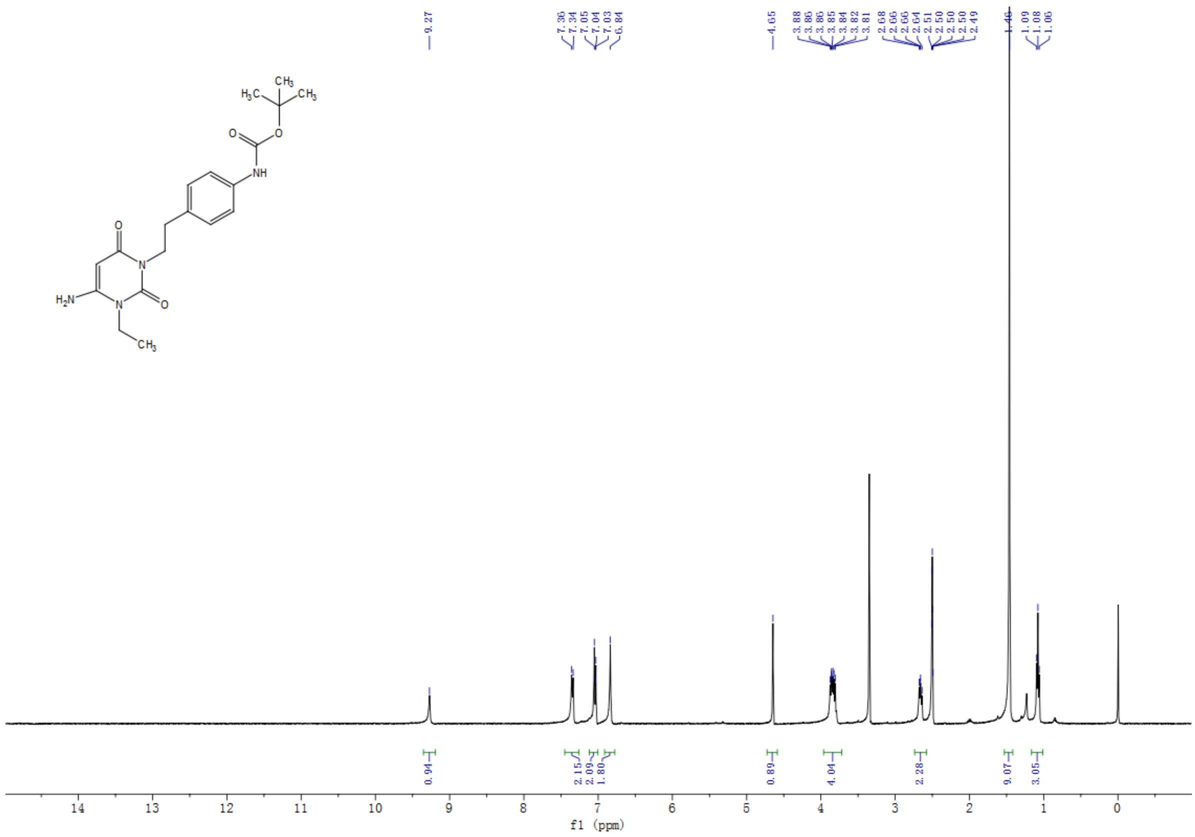

^{1}H NMR spectrum of compound **9h**

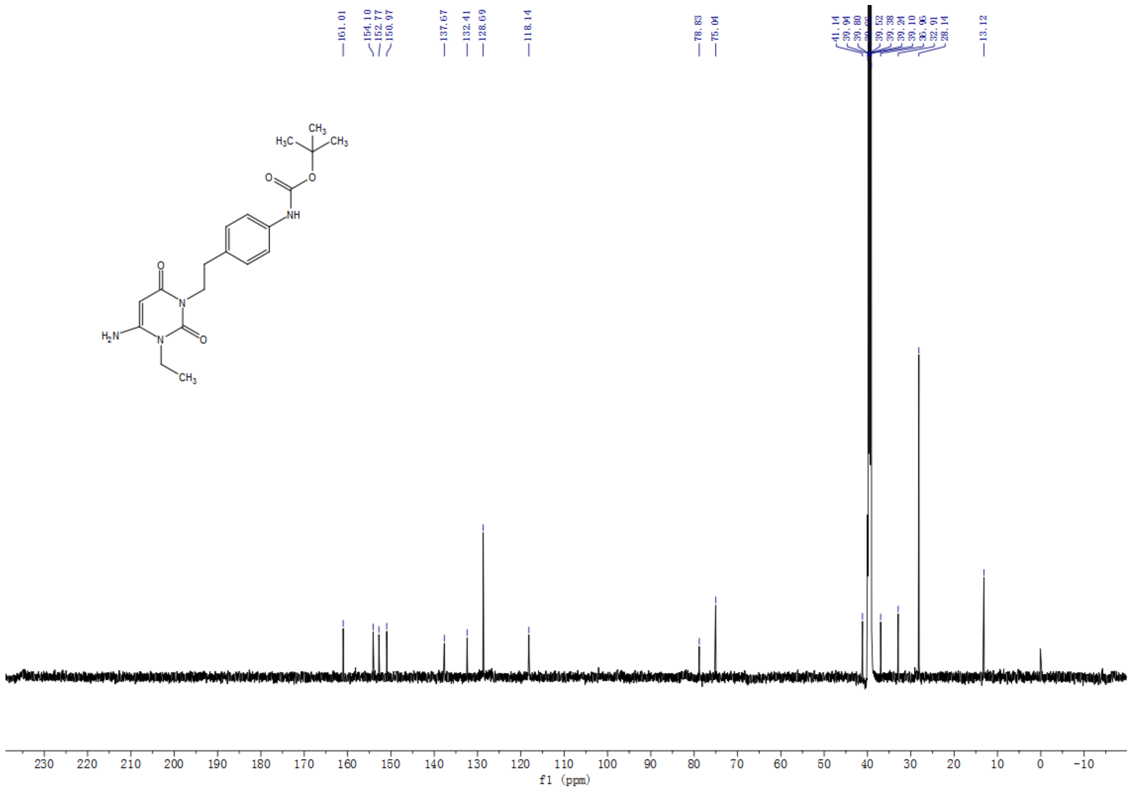

^{13}C NMR spectrum of compound **9h**

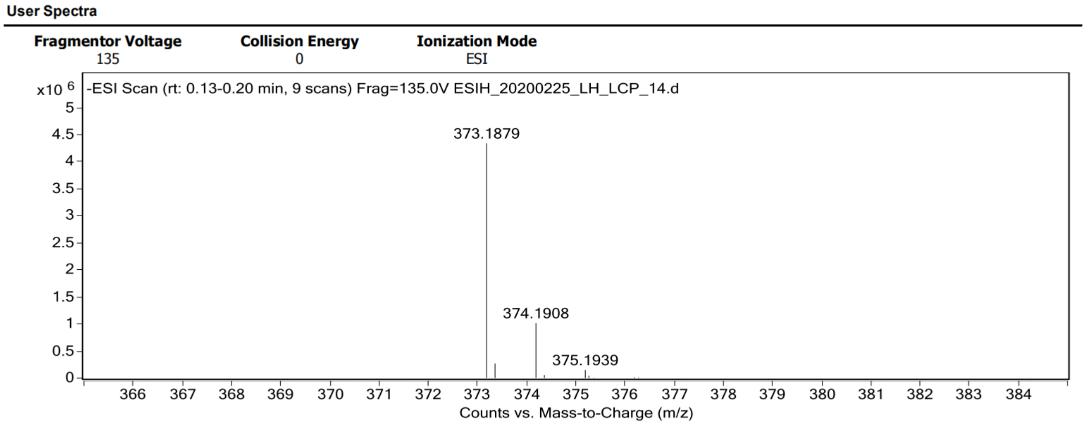

HRMS spectrum of compound **9h**

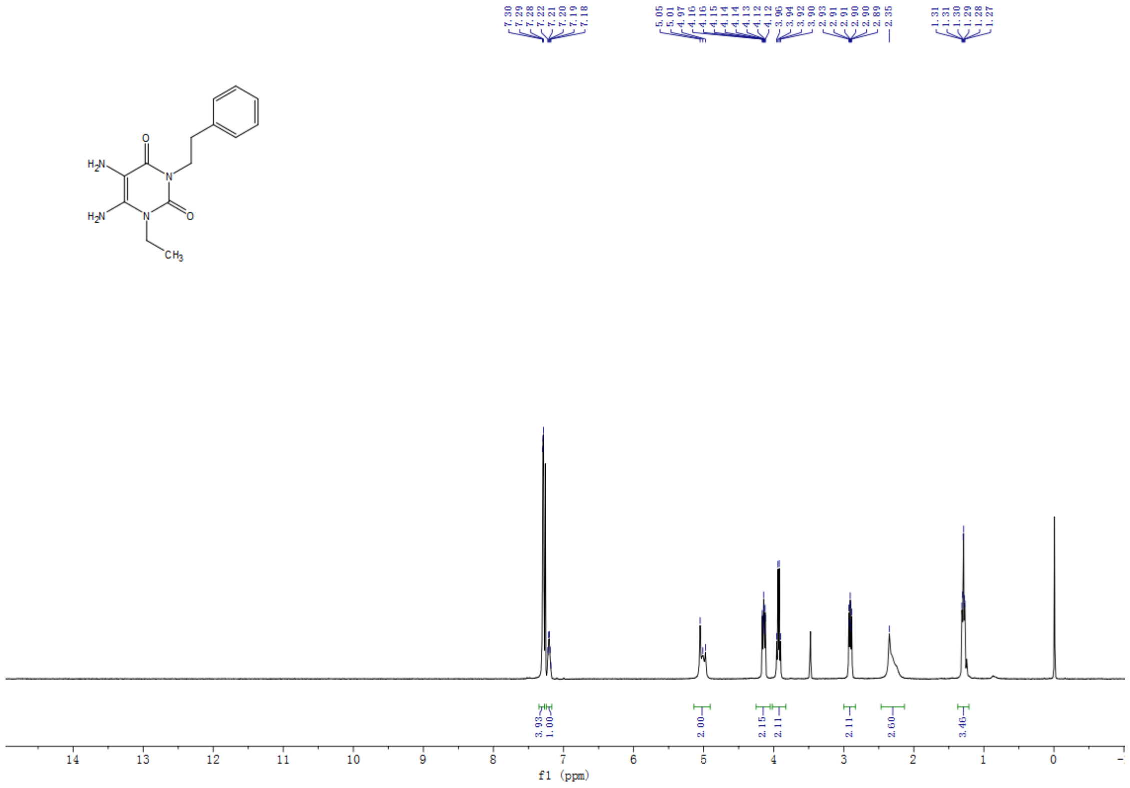

^{1}H NMR spectrum of compound **10a**

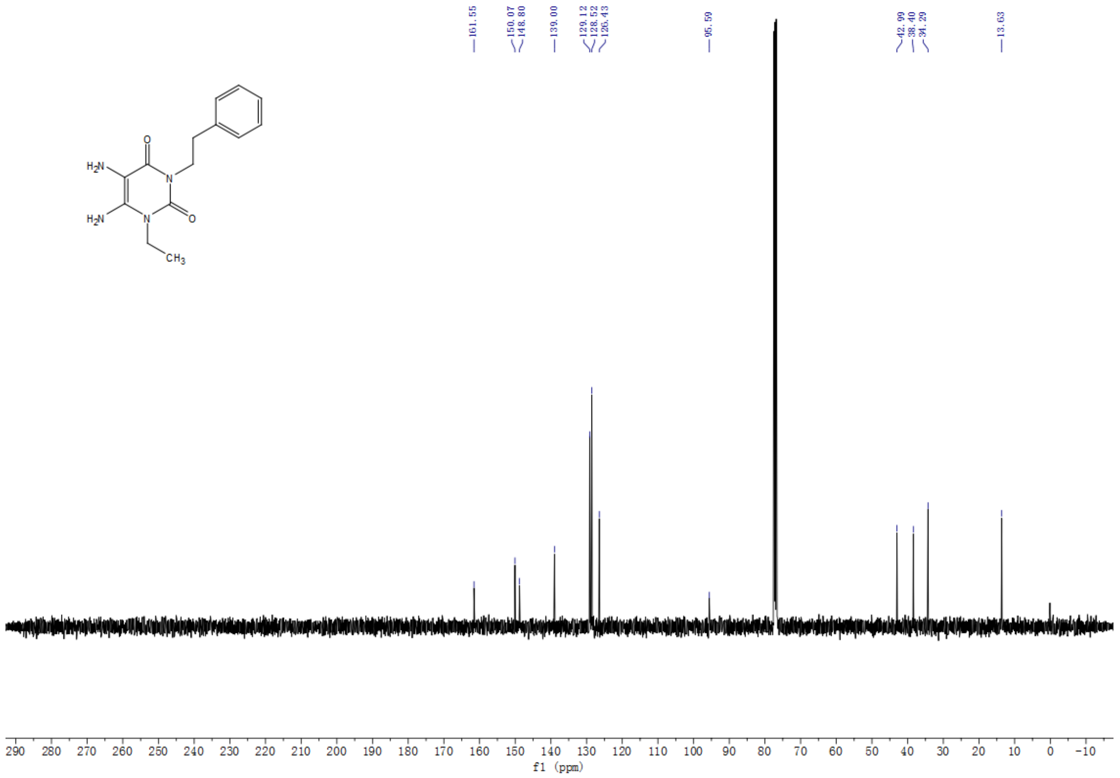

^{13}C NMR spectrum of compound **10a**

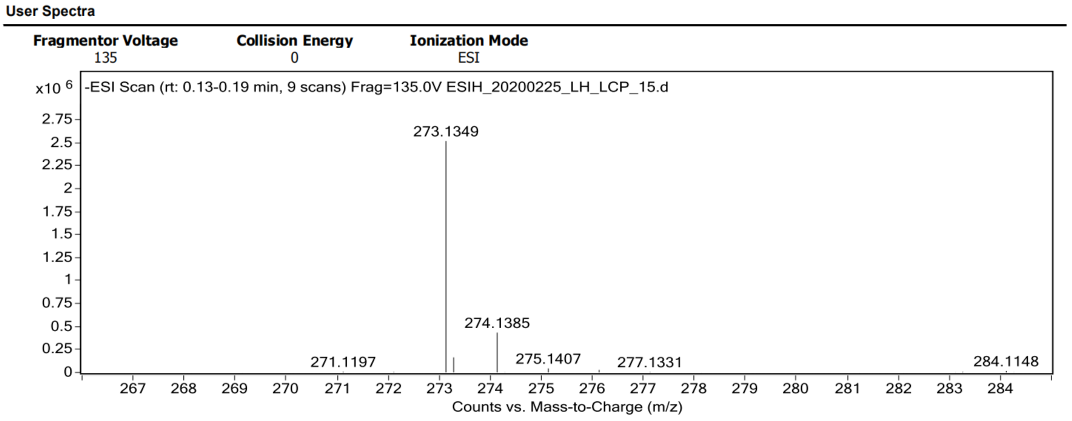

HRMS spectrum of compound **10a**

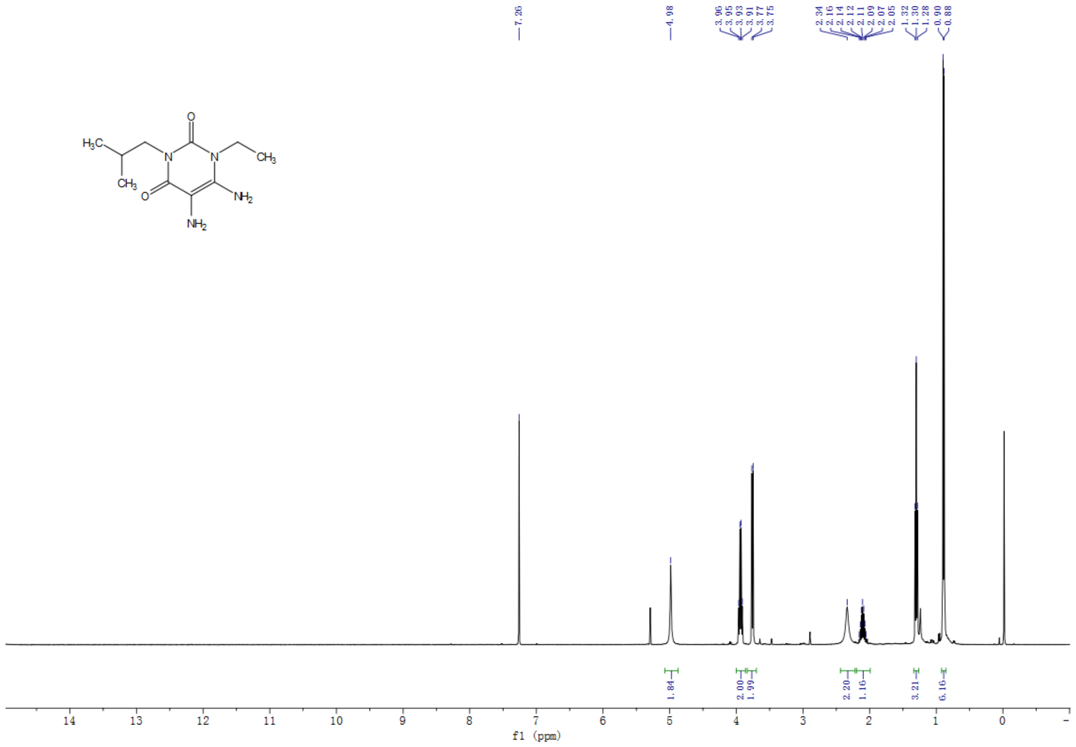

^{1}H NMR spectrum of compound **10b**

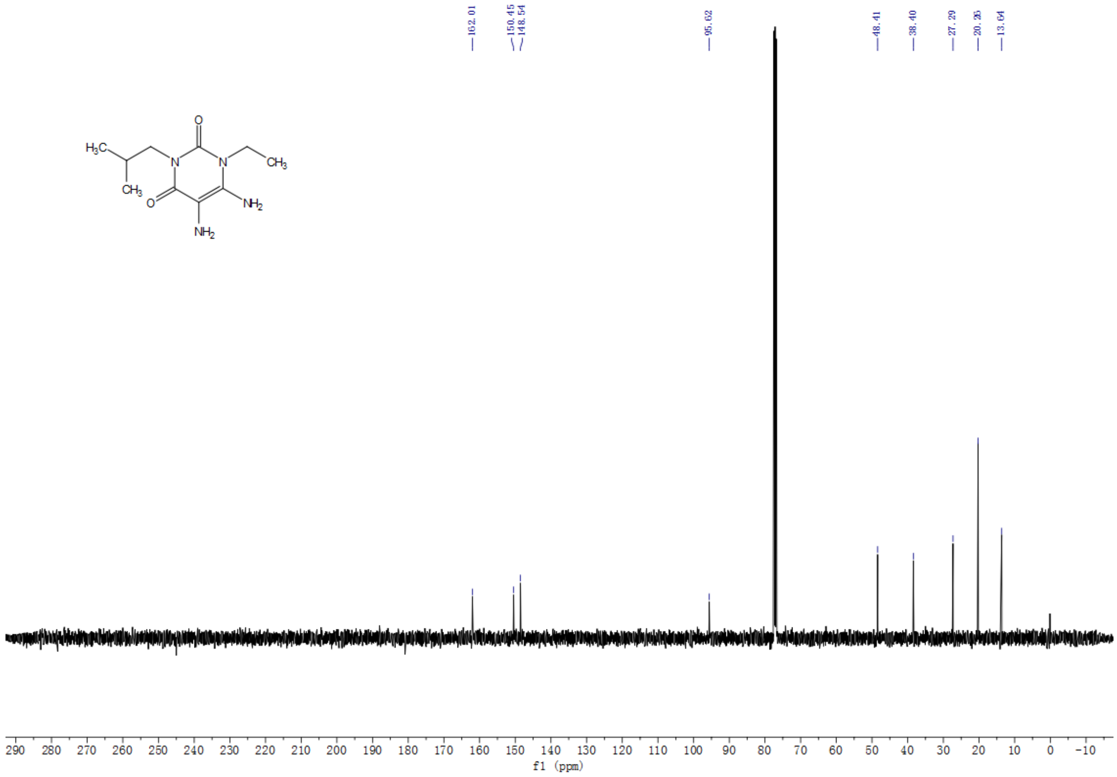

^{13}C NMR spectrum of compound **10b**

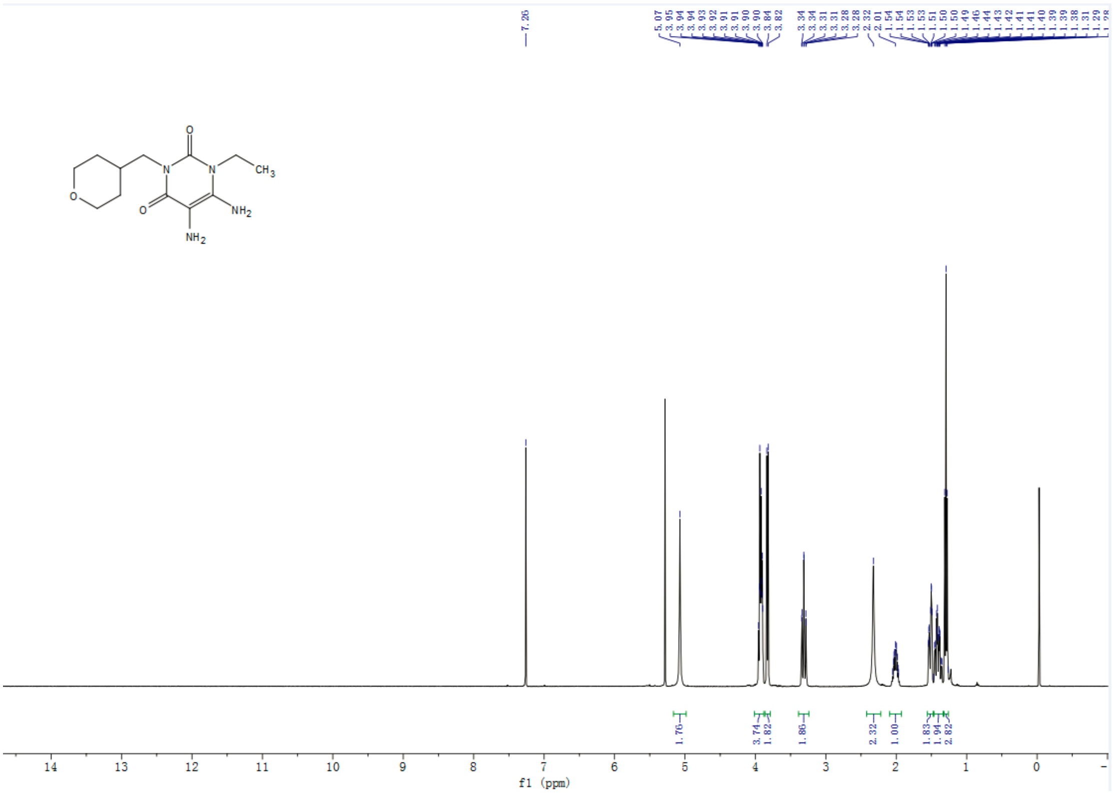

^{1}H NMR spectrum of compound **10c**

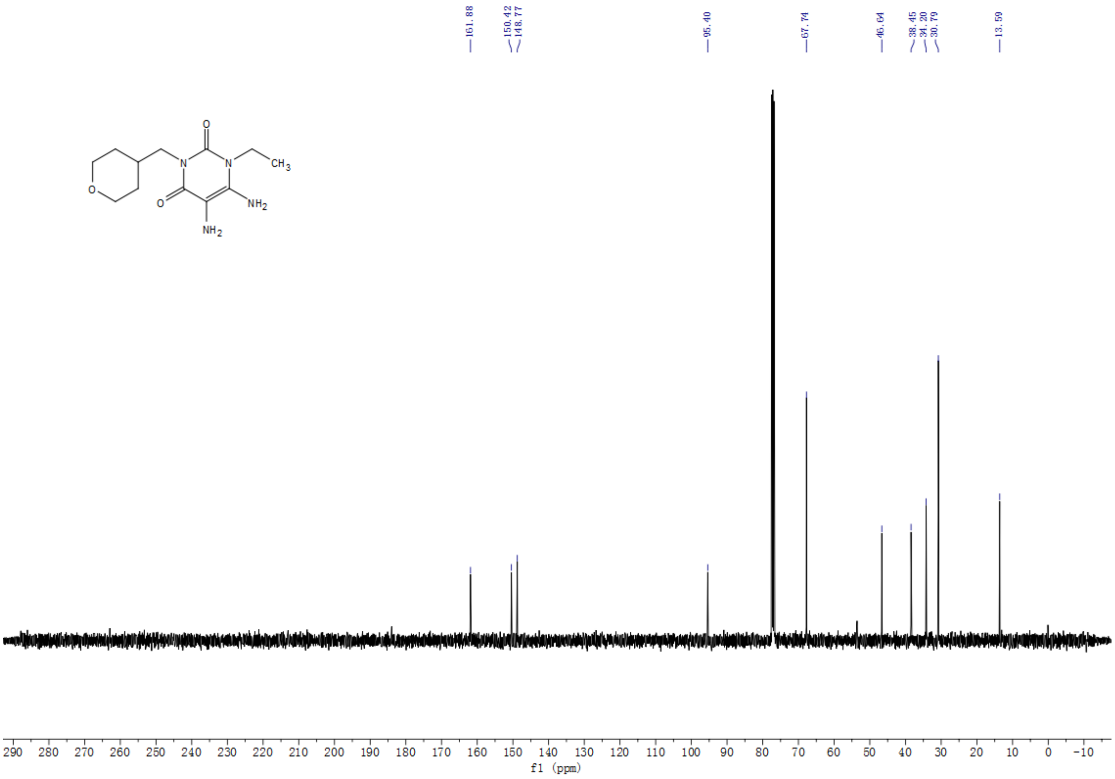

^{13}C NMR spectrum of compound **10c**

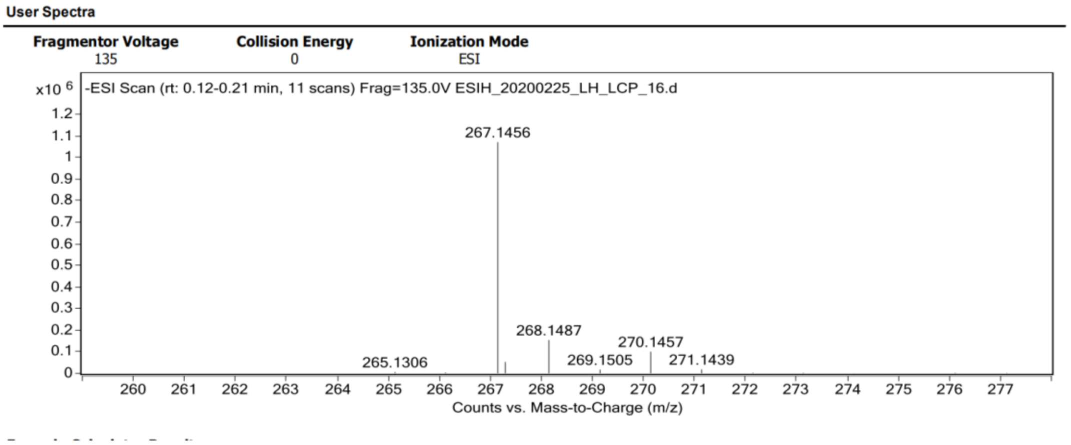

HRMS spectrum of compound **10c**

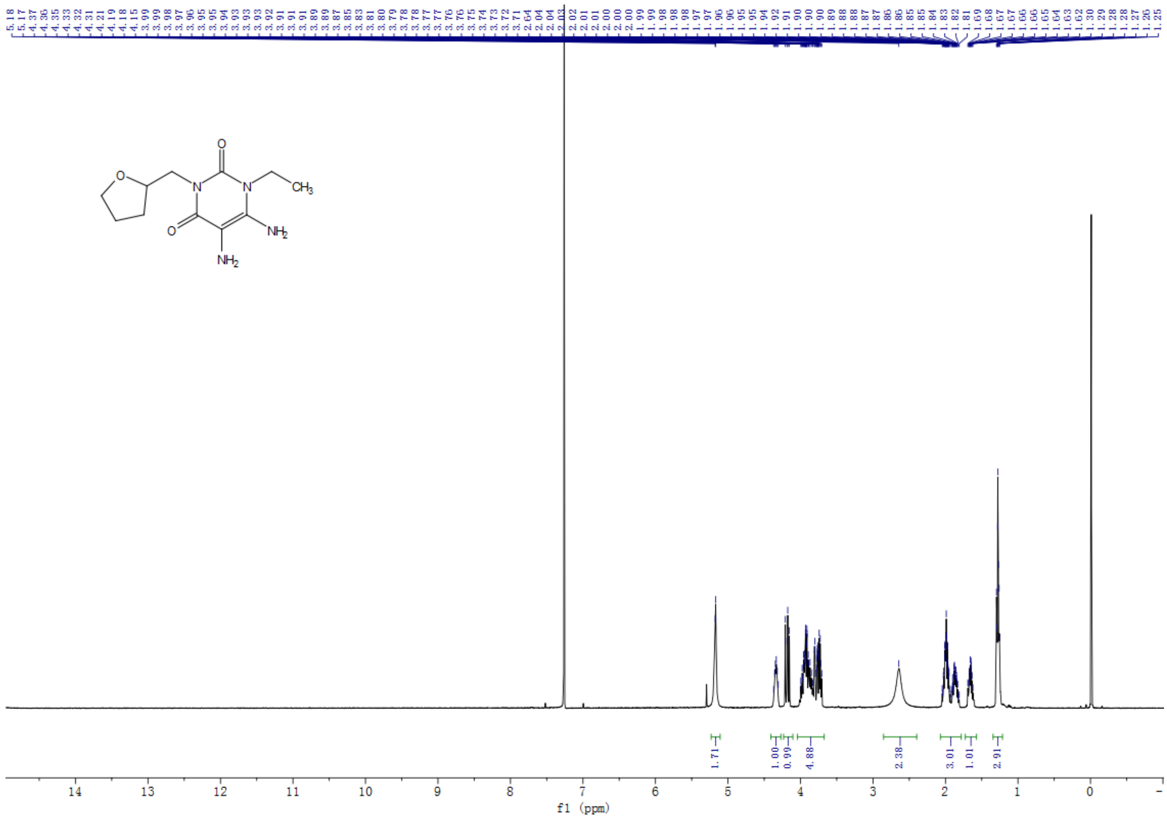

^{1}H NMR spectrum of compound **10d**

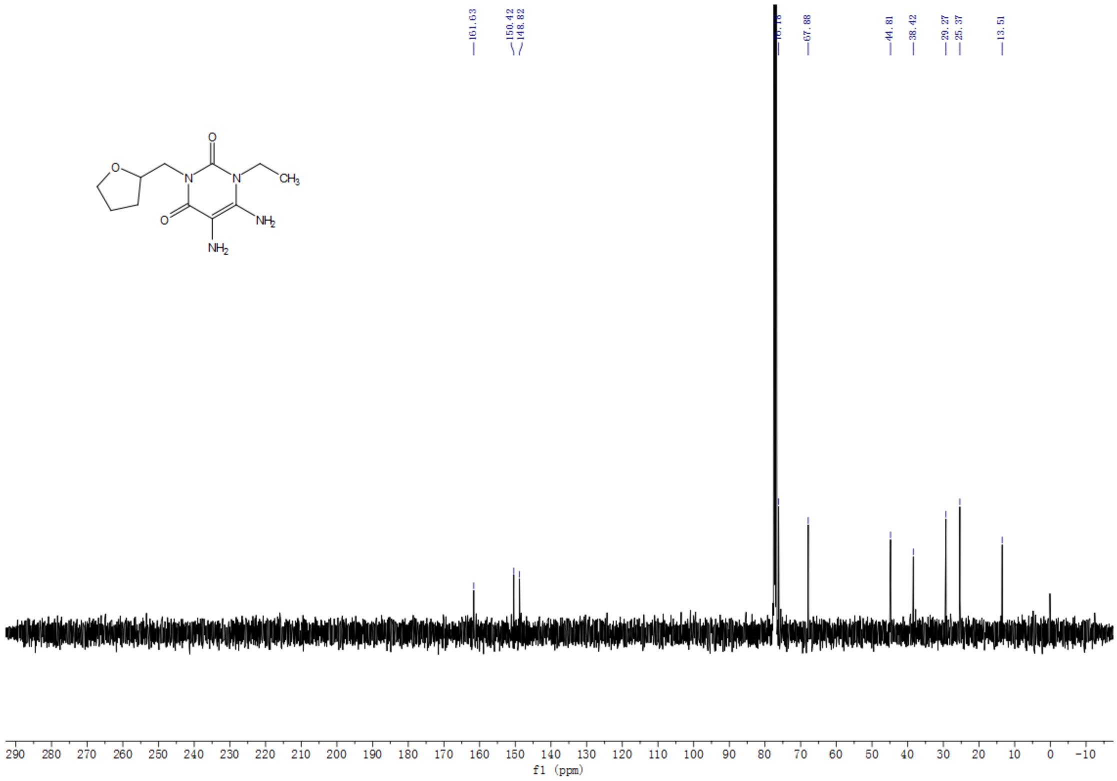

^{13}C NMR spectrum of compound **10d**

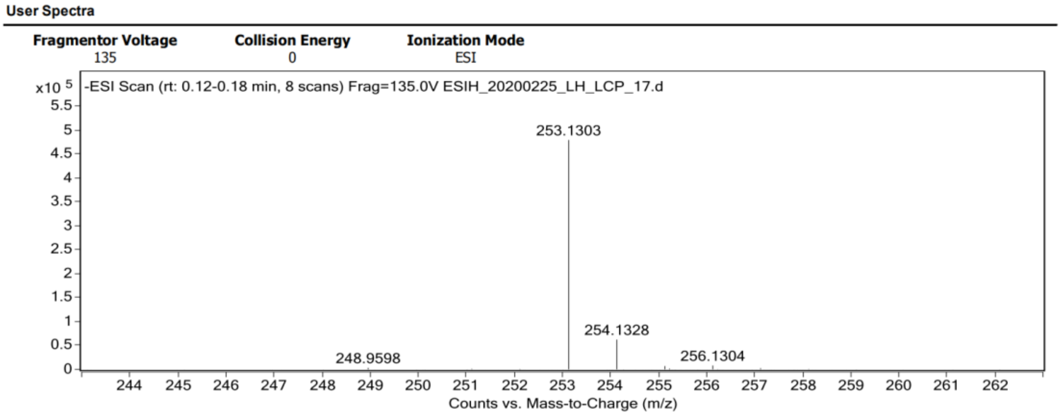

HRMS spectrum of compound **10d**

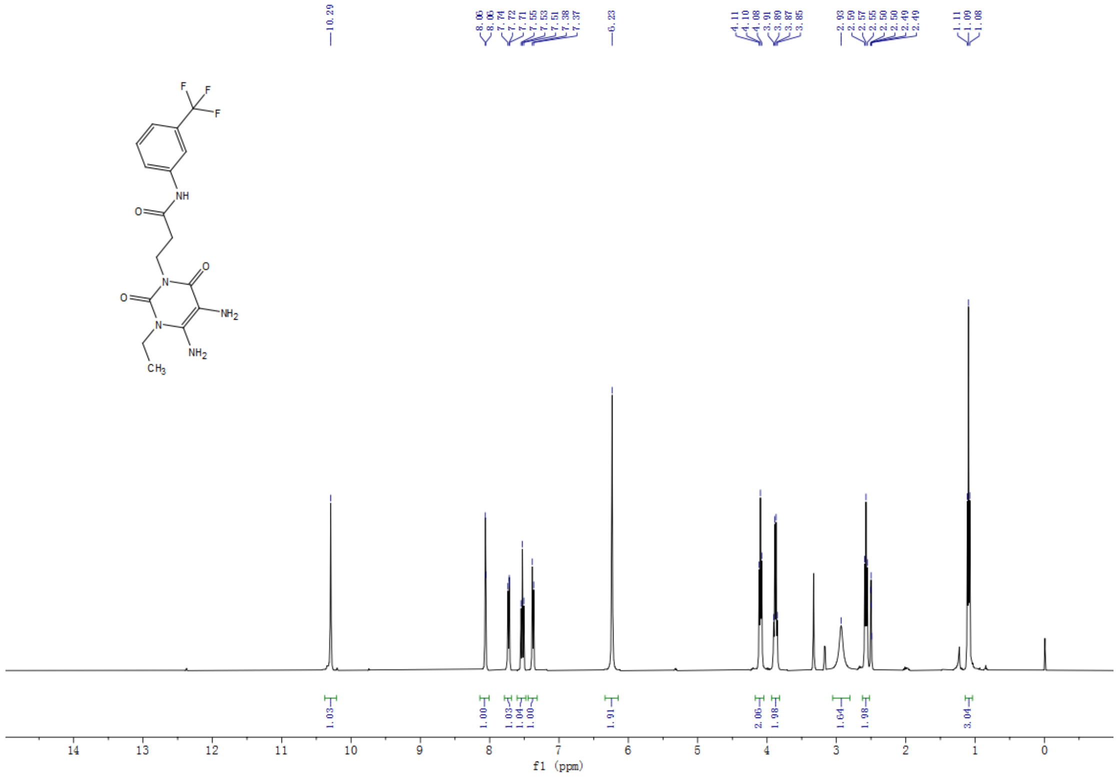

^{1}H NMR spectrum of compound **10e**

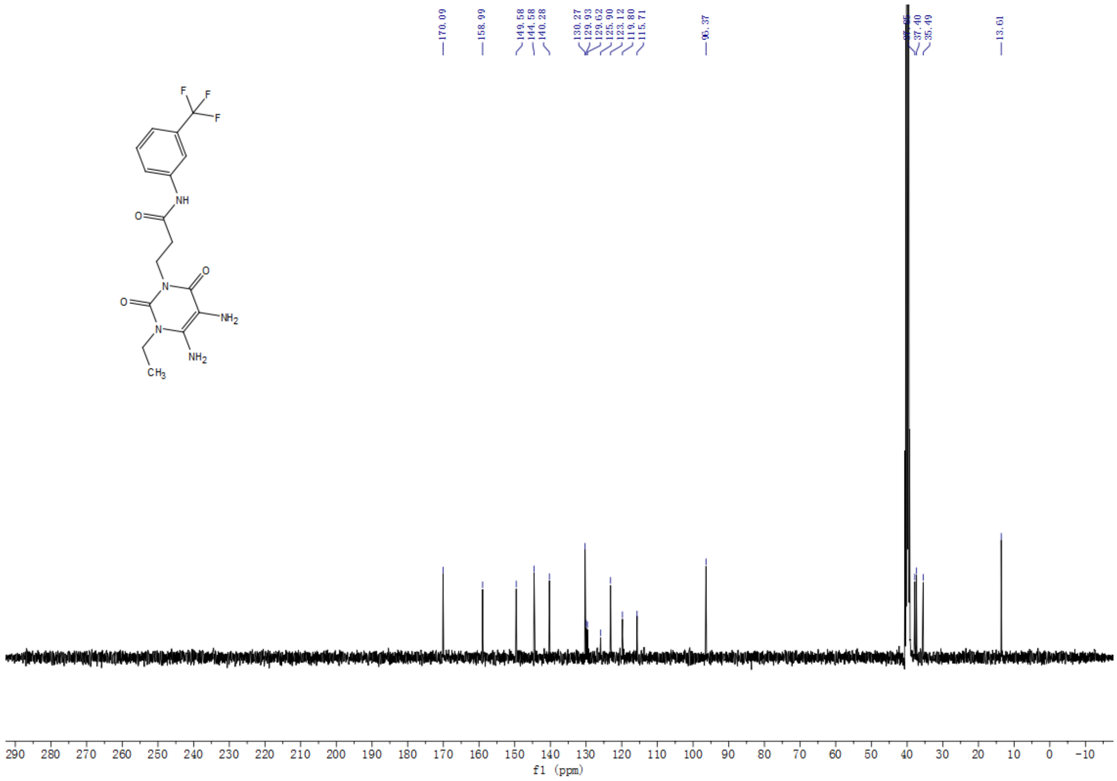

^{13}C NMR spectrum of compound **10e**

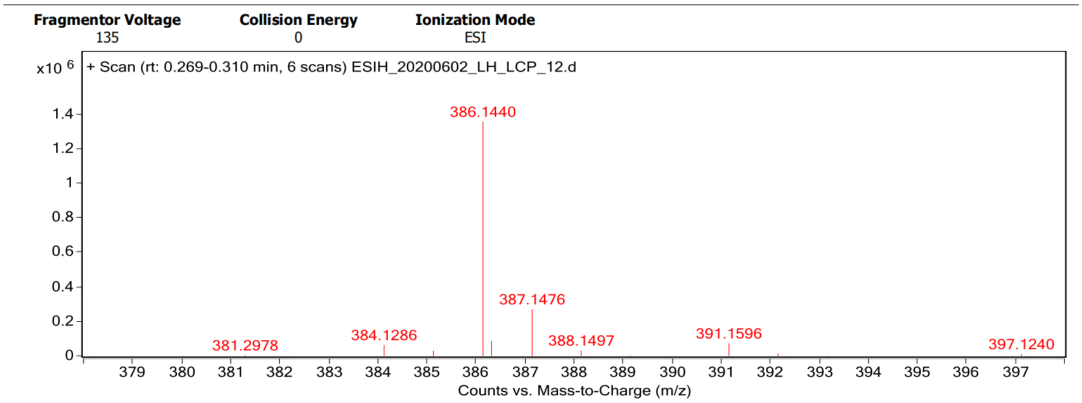

HRMS spectrum of compound **10e**

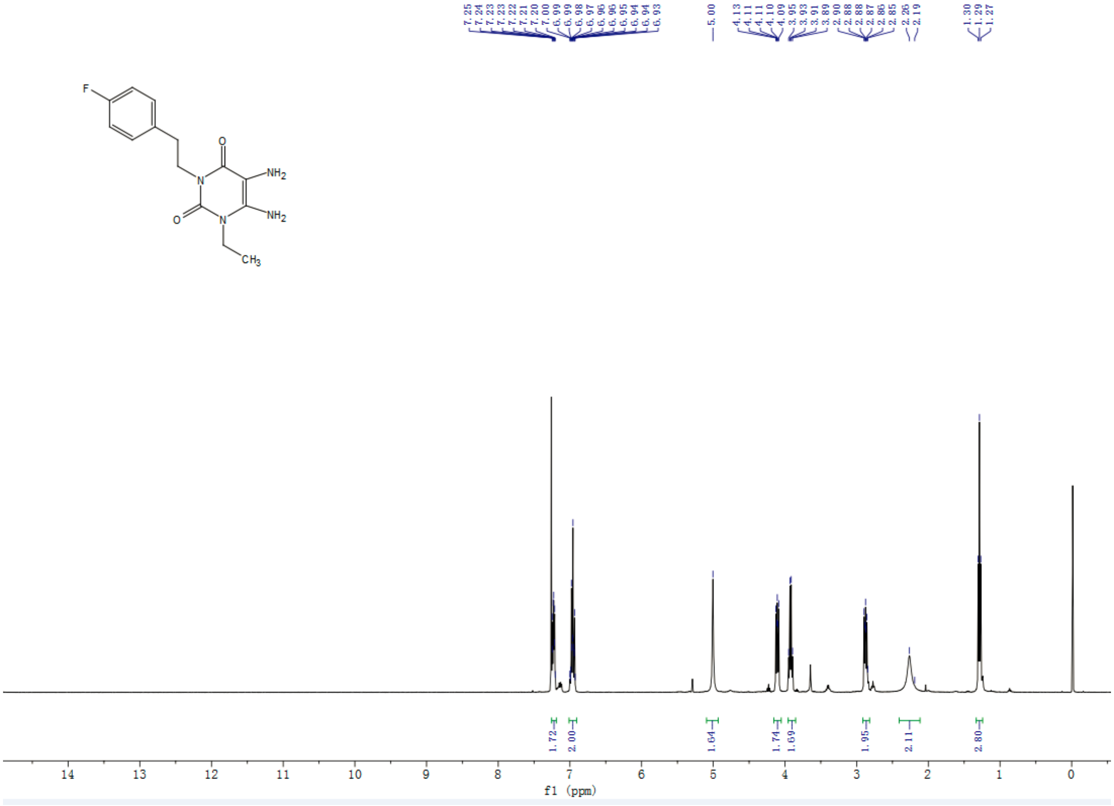

^{1}H NMR spectrum of compound **10f**

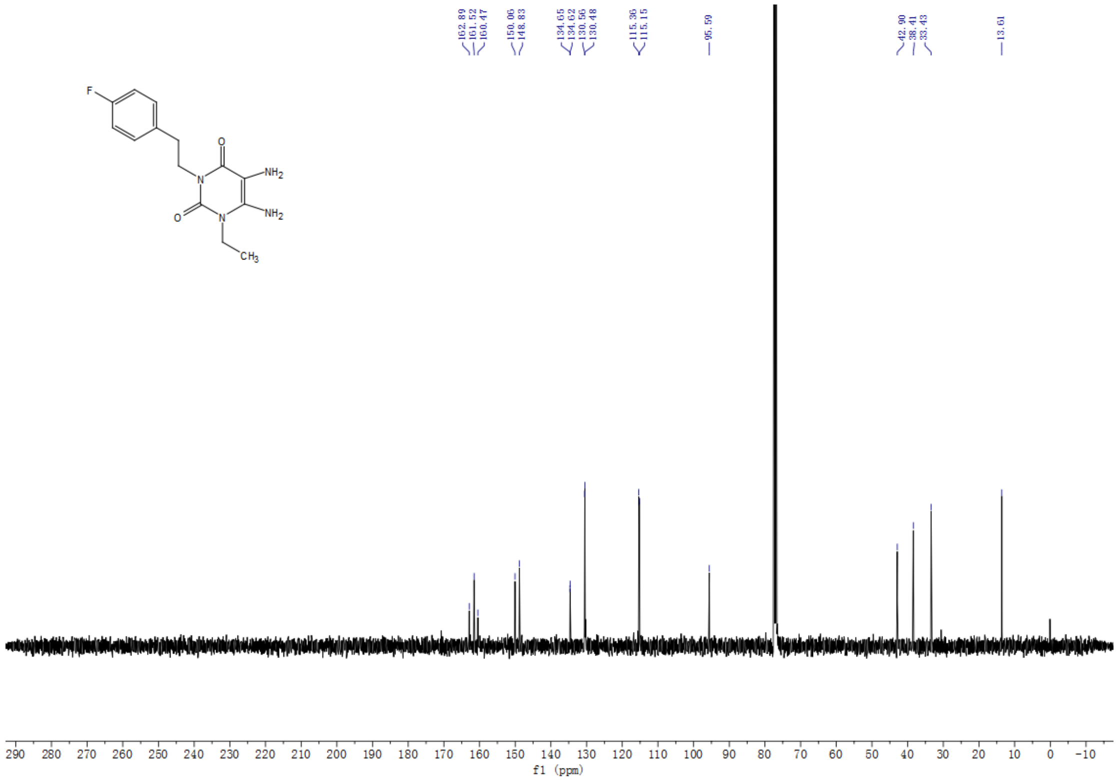

^{13}C NMR spectrum of compound **10f**

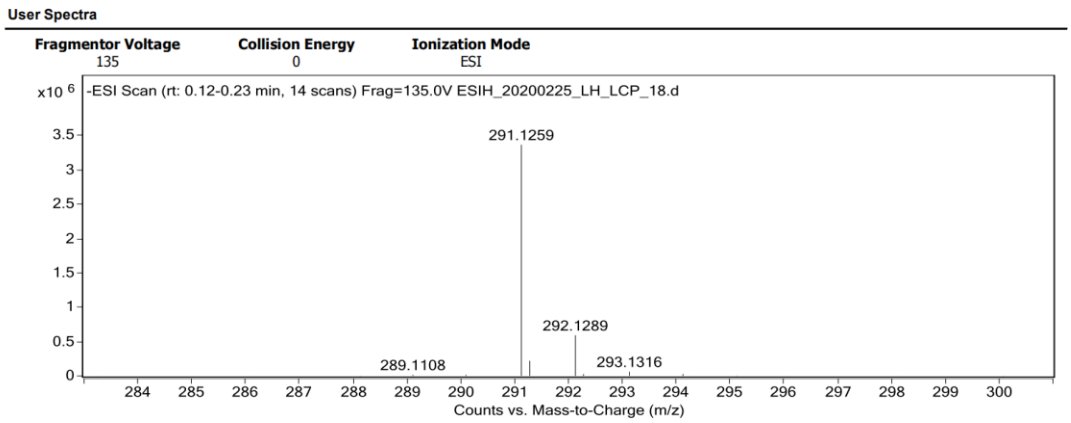

HRMS spectrum of compound **10f**

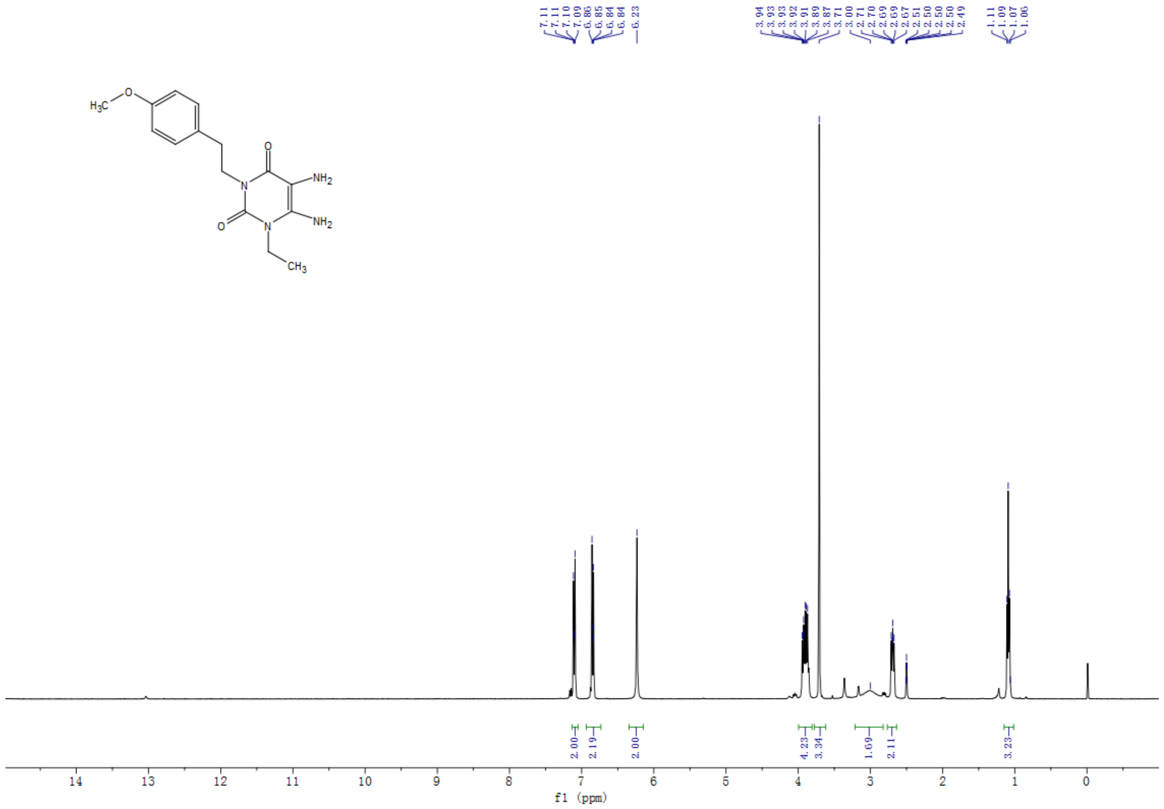

^{1}H NMR spectrum of compound **10g**

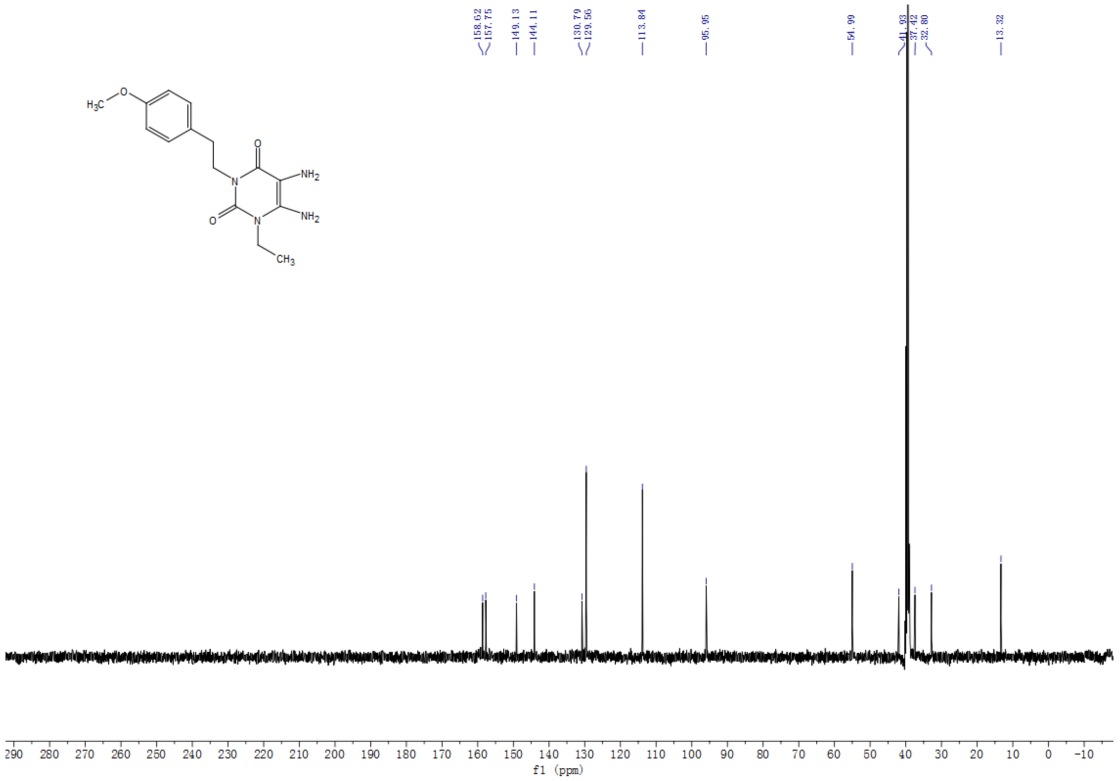

^{13}C NMR spectrum of compound **10g**

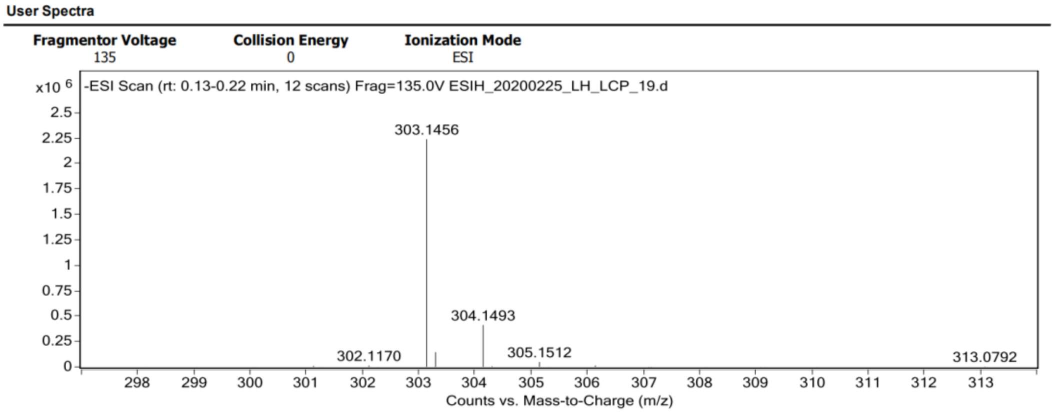

HRMS spectrum of compound **10g**

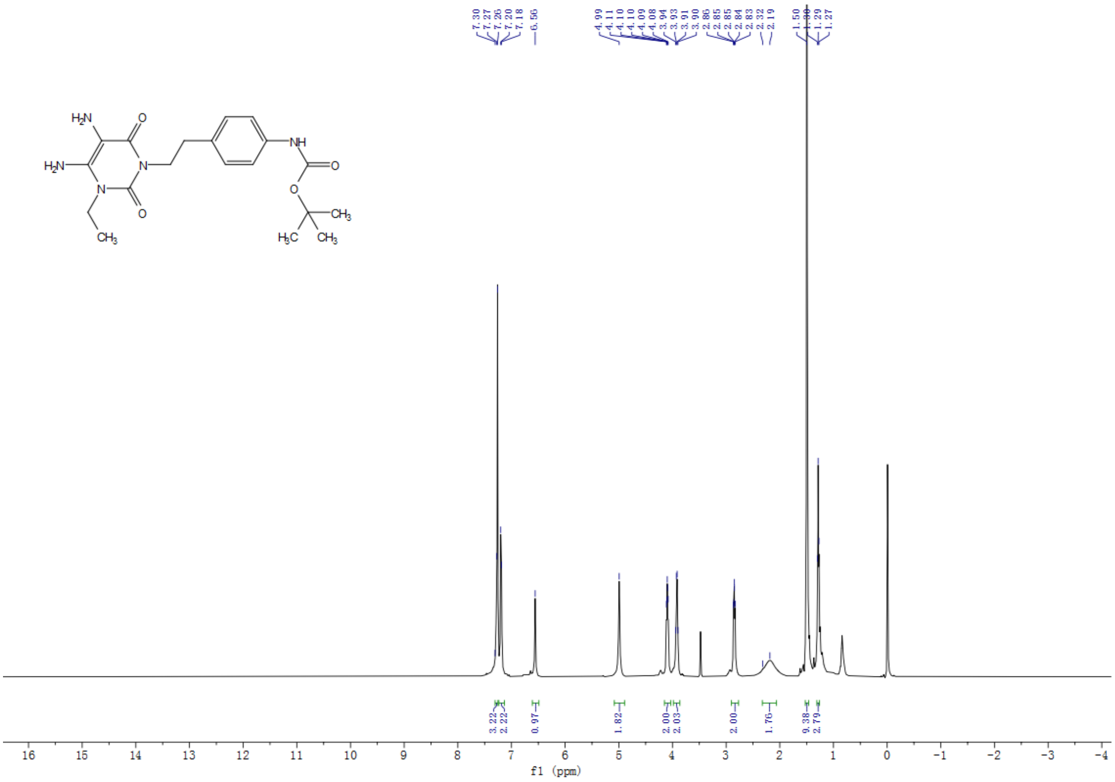

^{1}H NMR spectrum of compound **10h**

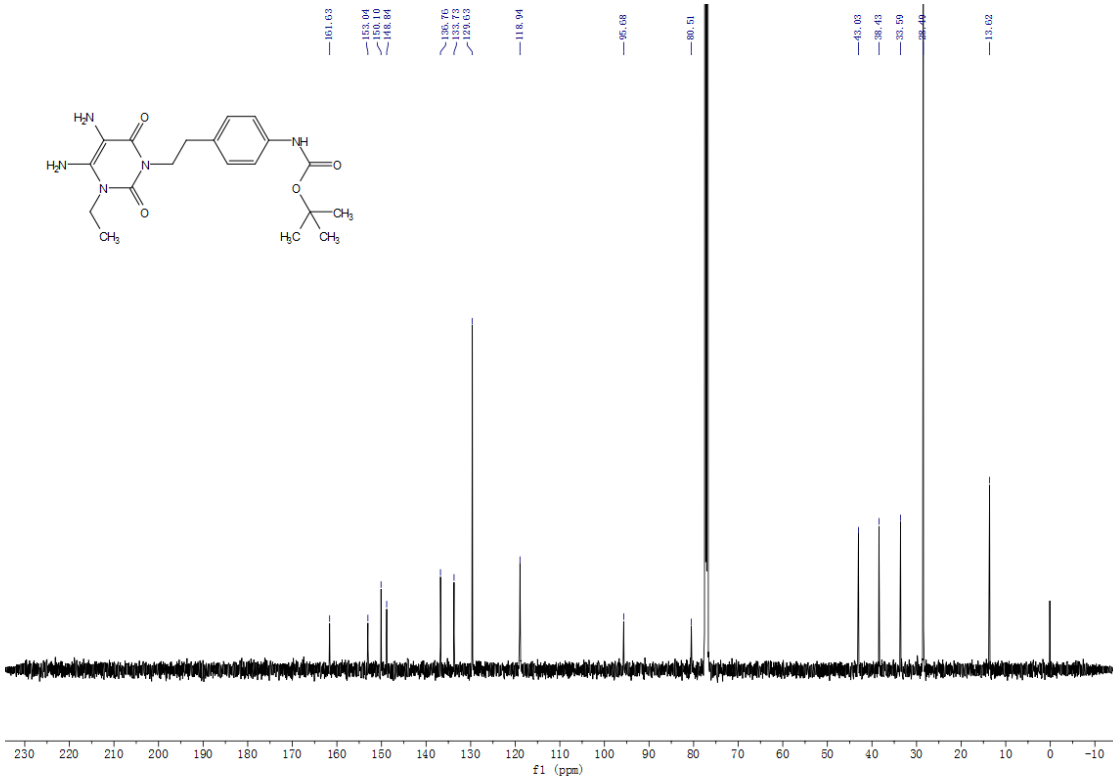

^{13}C NMR spectrum of compound **10h**

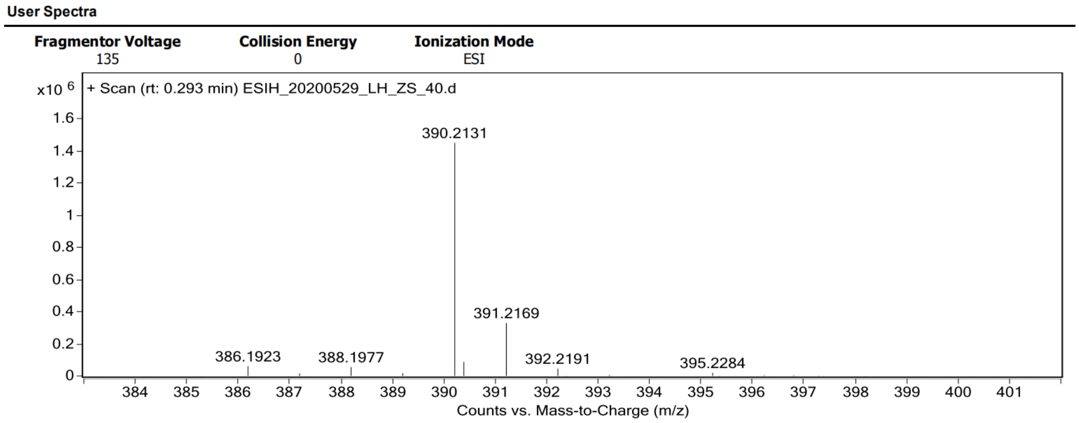

HRMS spectrum of compound **10h**

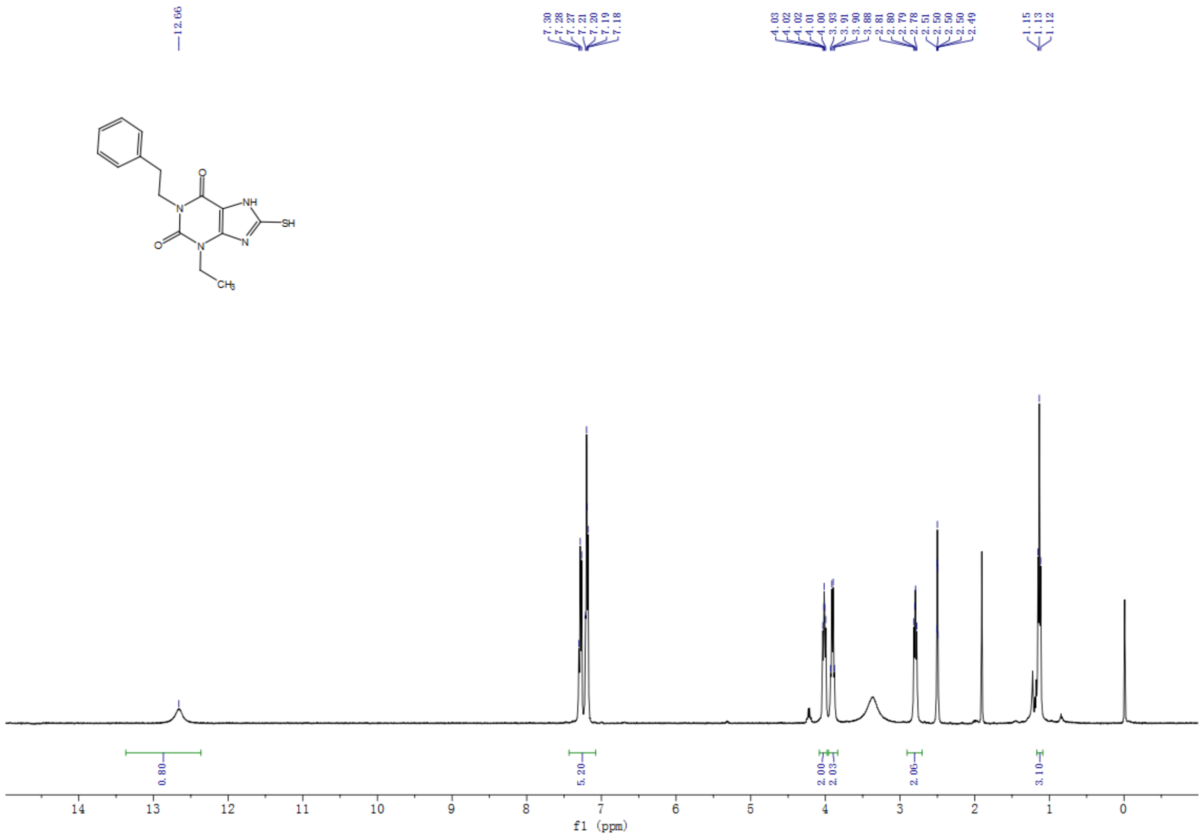

^{1}H NMR spectrum of compound **11a**

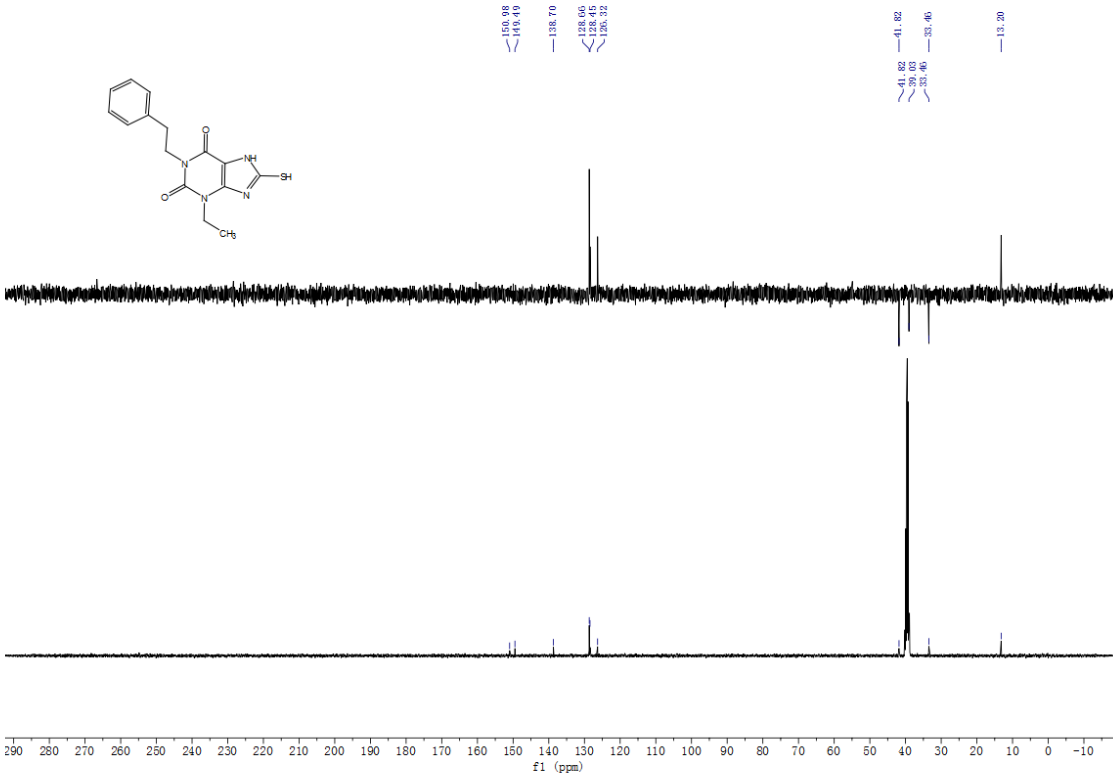

^{13}C NMR spectrum (DEPT+BB) of compound **11a**

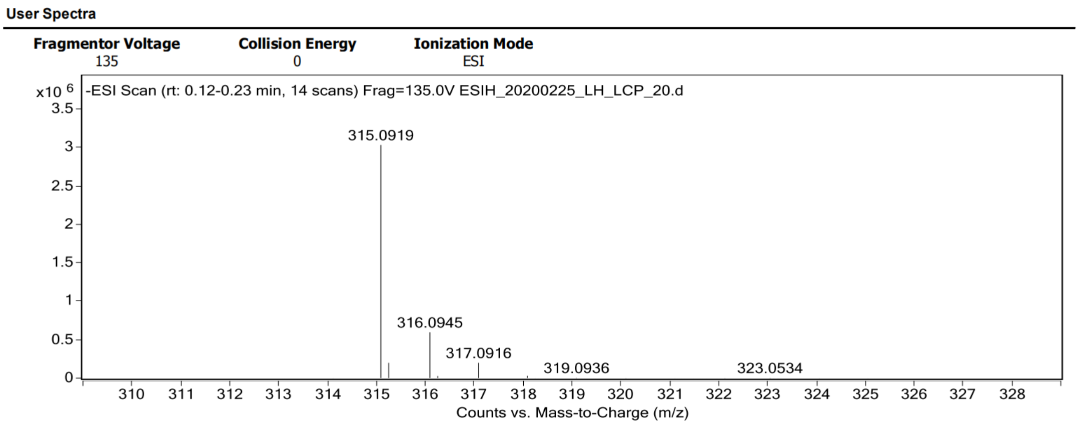

HRMS spectrum of compound **11a**

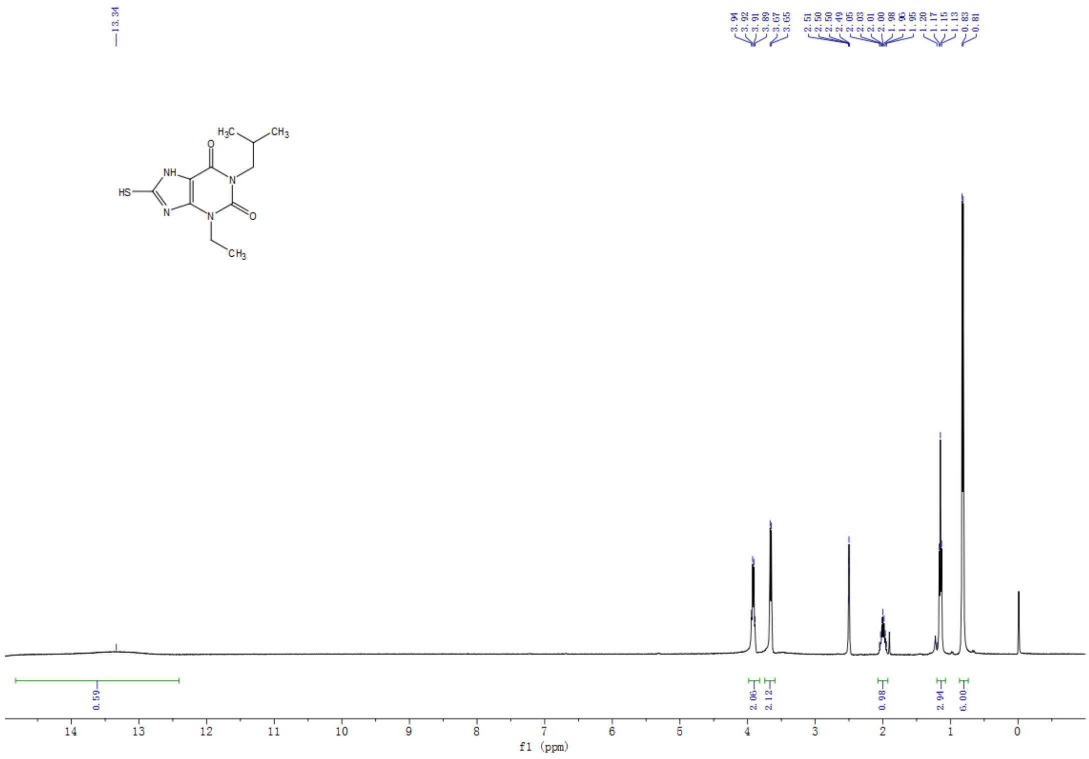

^{1}H NMR spectrum of compound **11b**

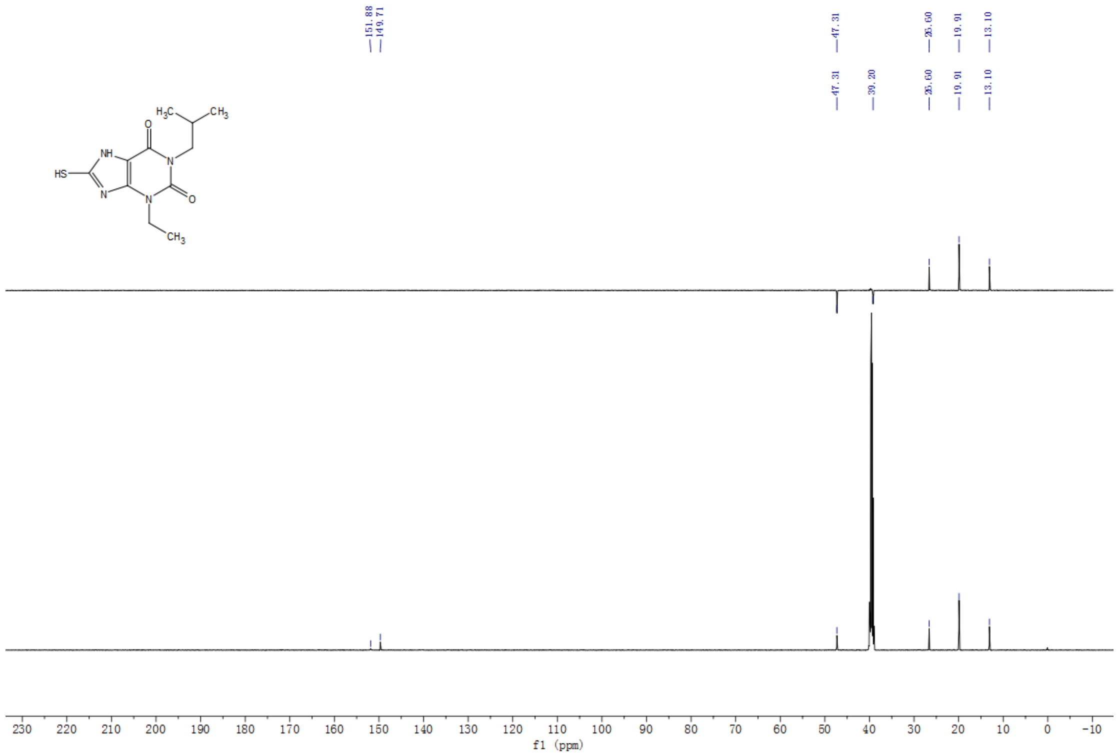

^{13}C NMR spectrum (DEPT+BB) of compound **11b**

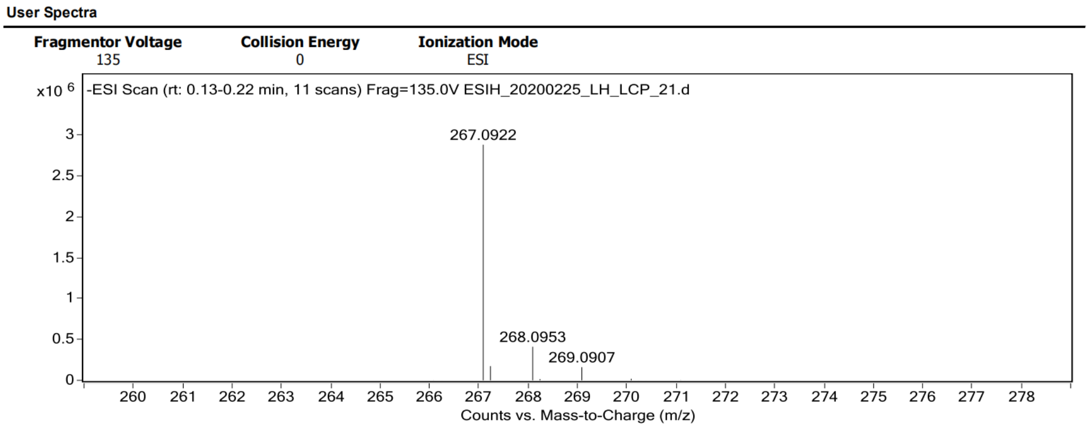

HRMS spectrum of compound **11b**

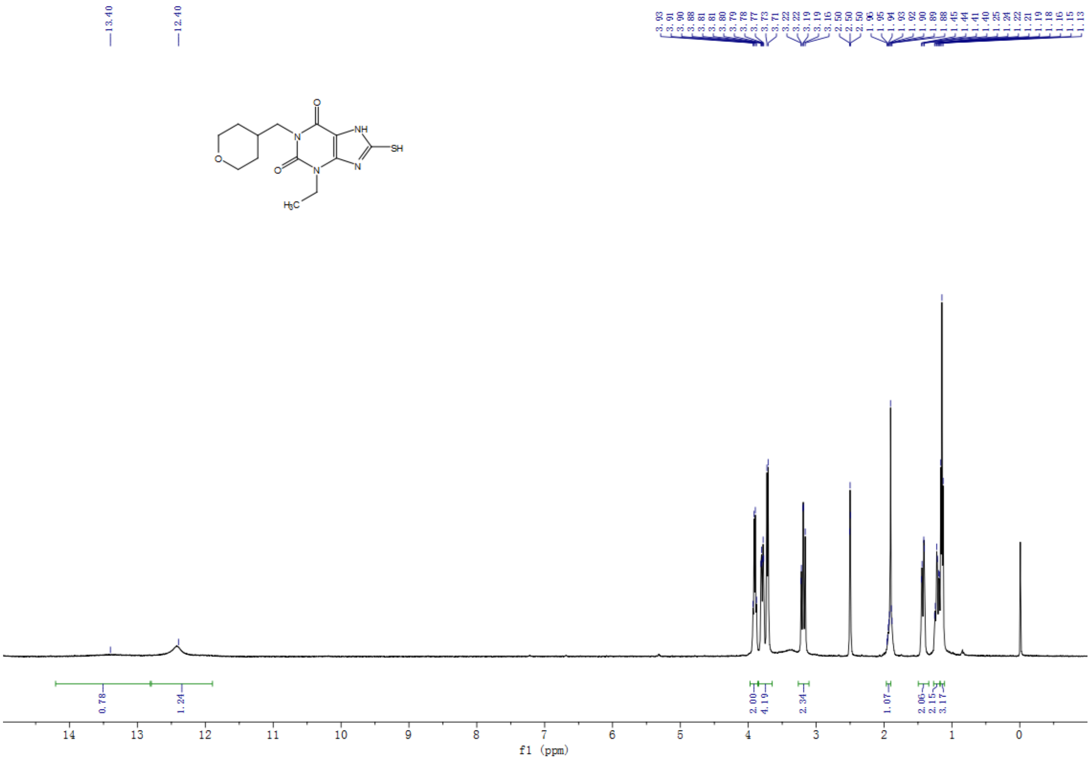

^{1}H NMR spectrum of compound **11c**

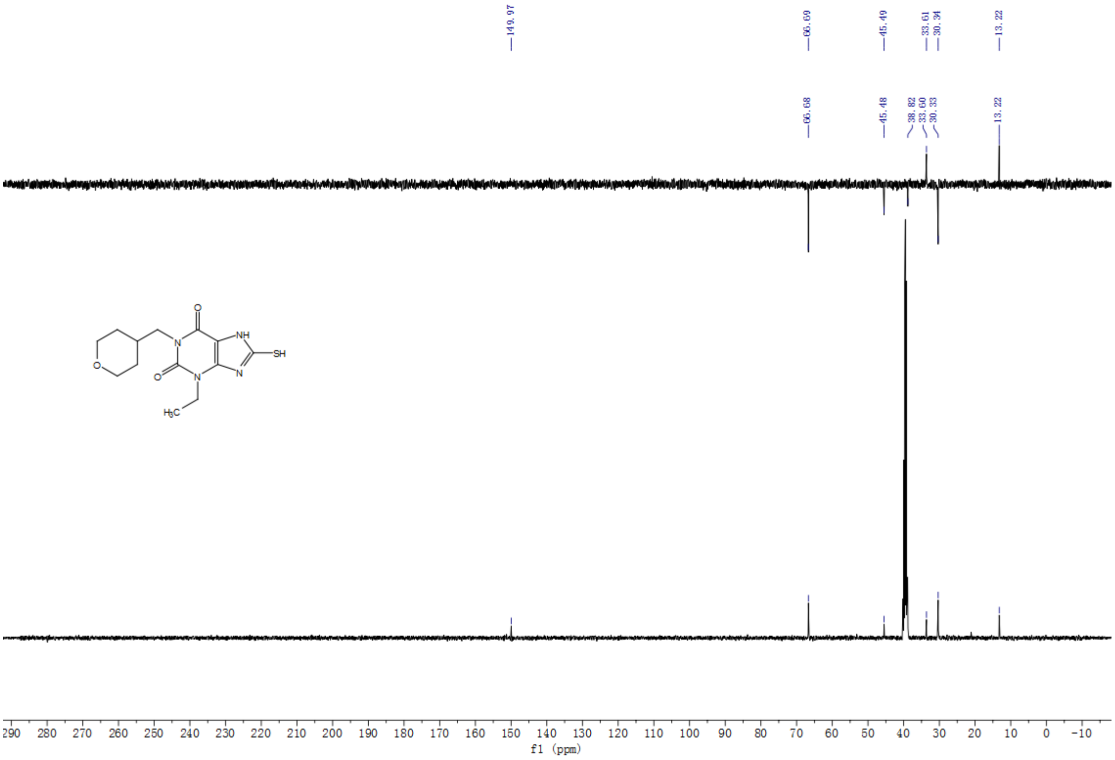

^{13}C NMR spectrum (DEPT+BB) of compound **11c**

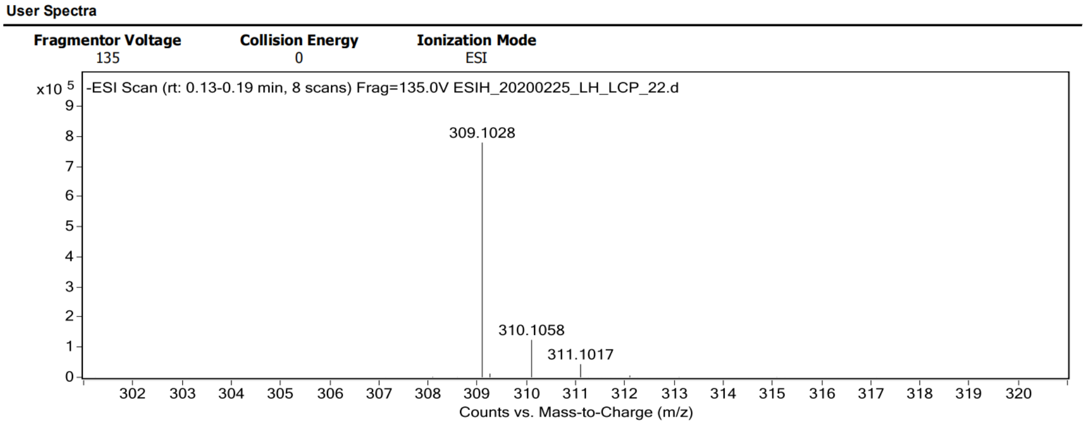

HRMS spectrum of compound **11c**

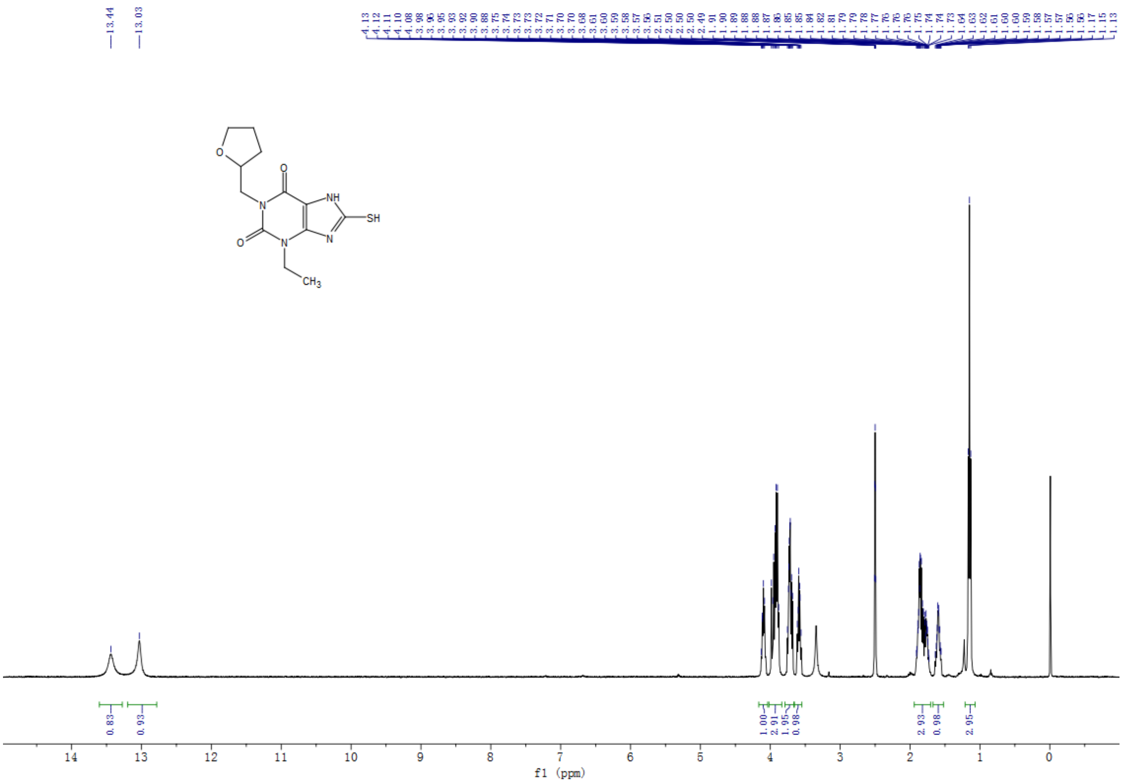

^{1}H NMR spectrum of compound **11d**

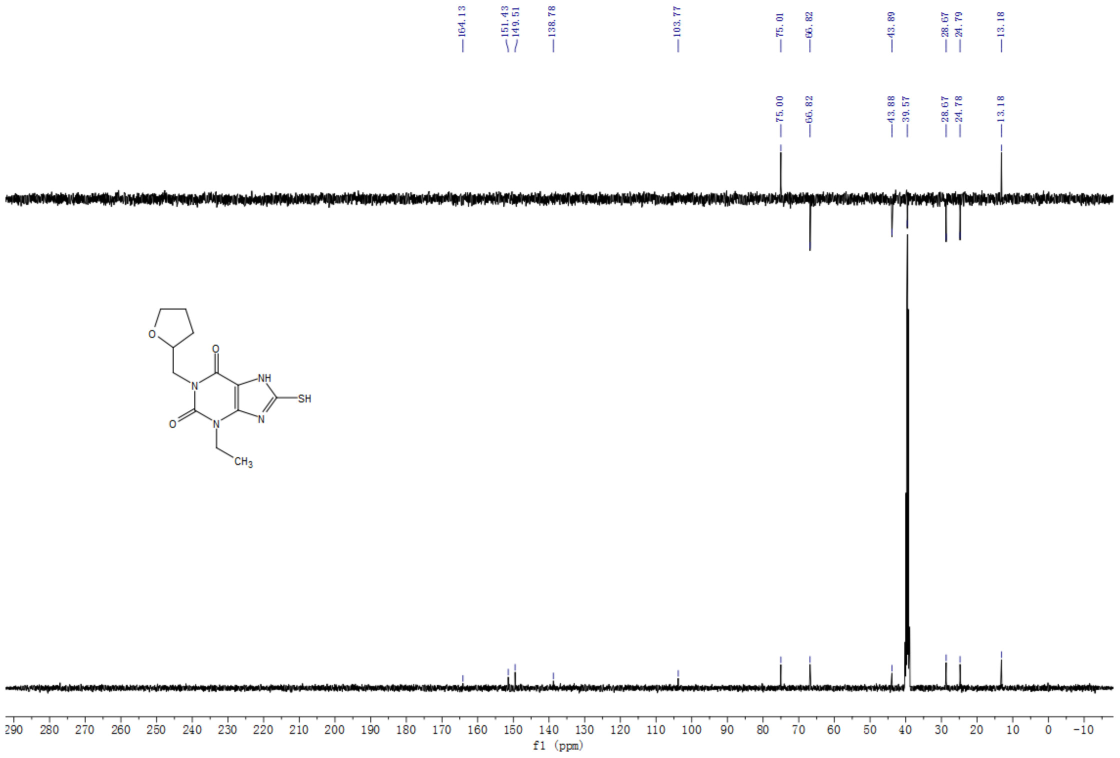

^{13}C NMR spectrum (DEPT+BB) of compound **11d**

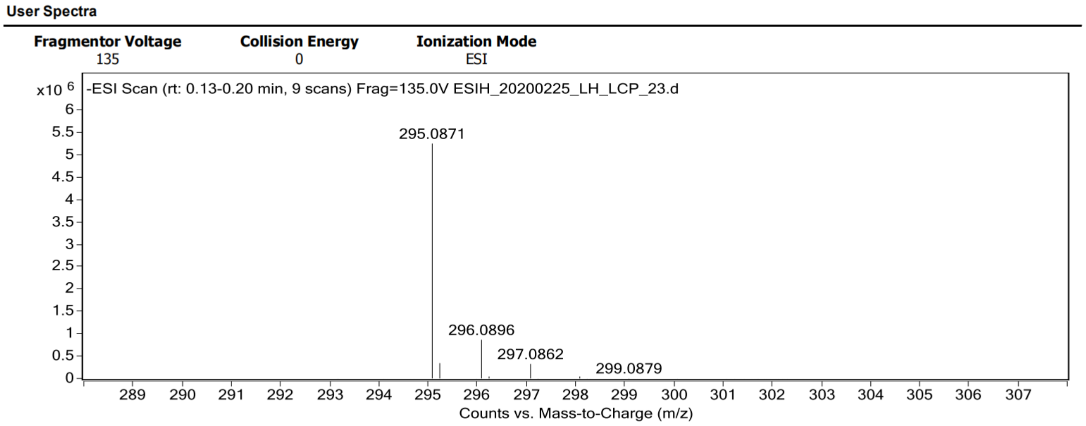

HRMS spectrum of compound **11d**

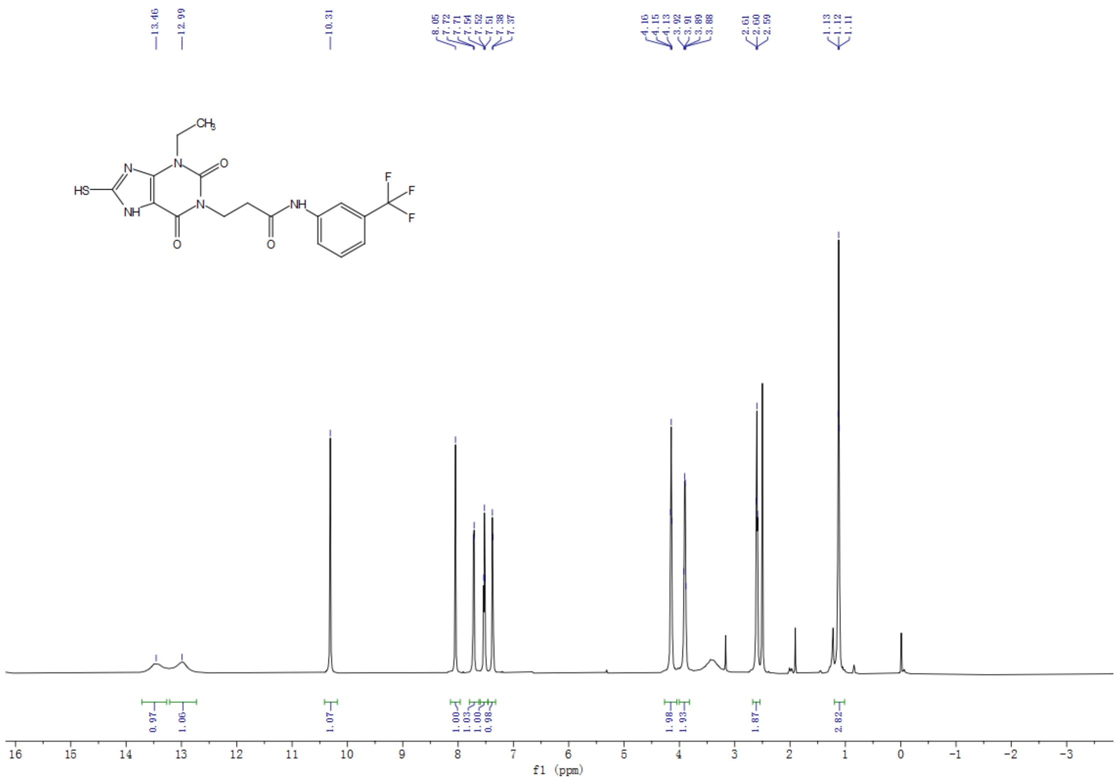

^{1}H NMR spectrum of compound **11e**

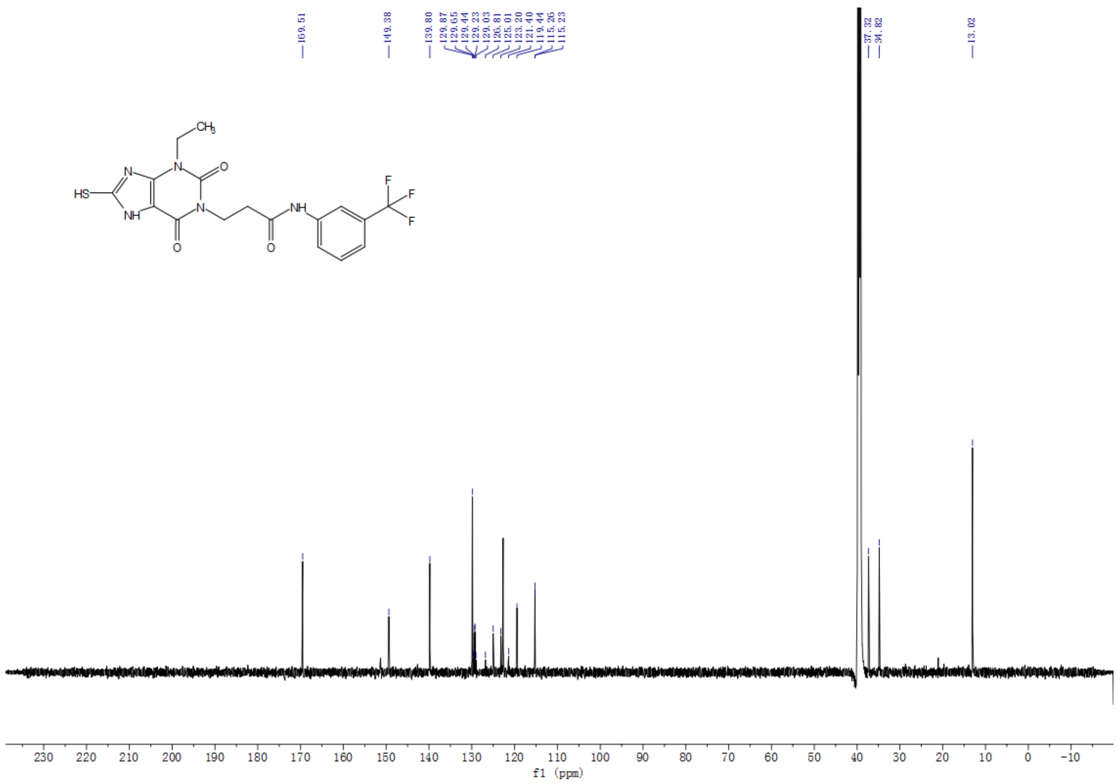

^{13}C NMR spectrum of compound **11e**

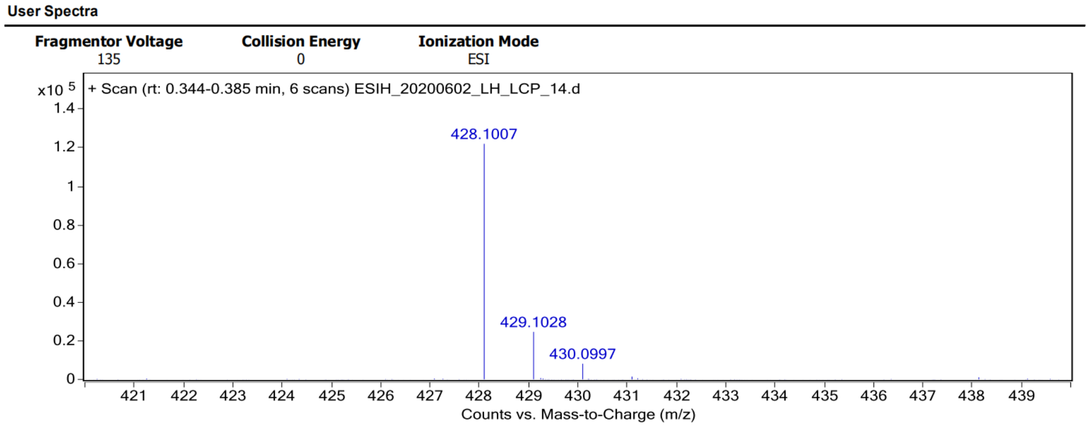

HRMS spectrum of compound **11e**

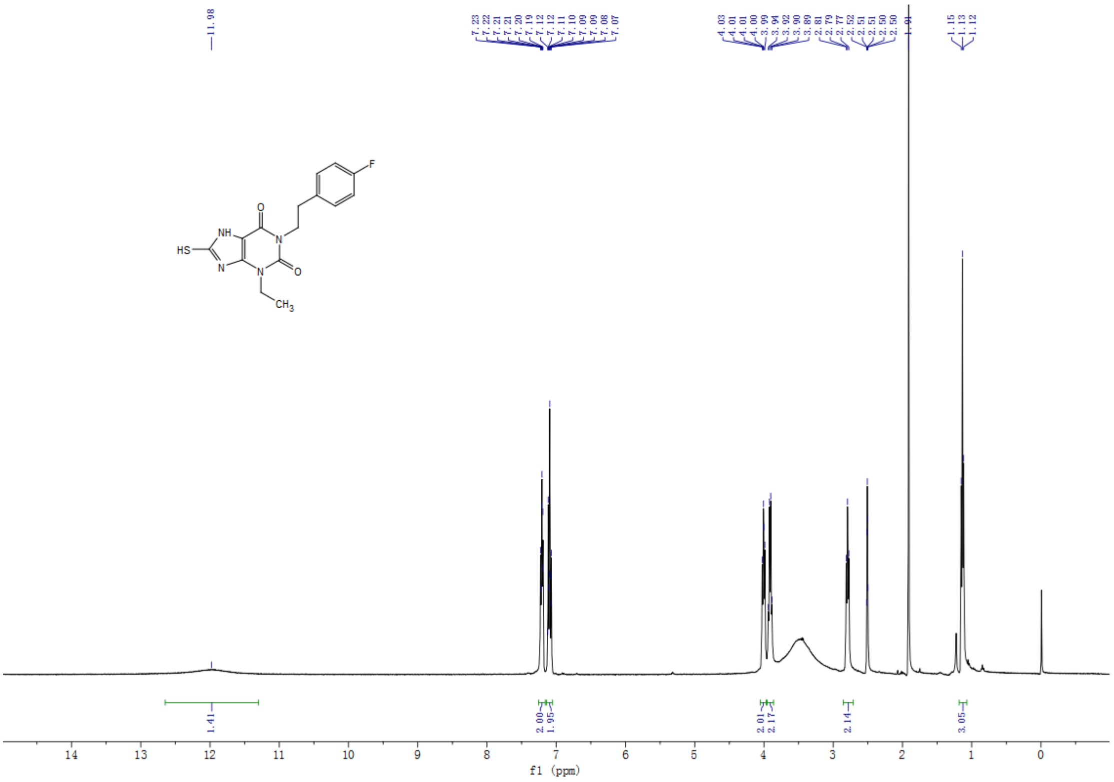

^{1}H NMR spectrum of compound **11f**

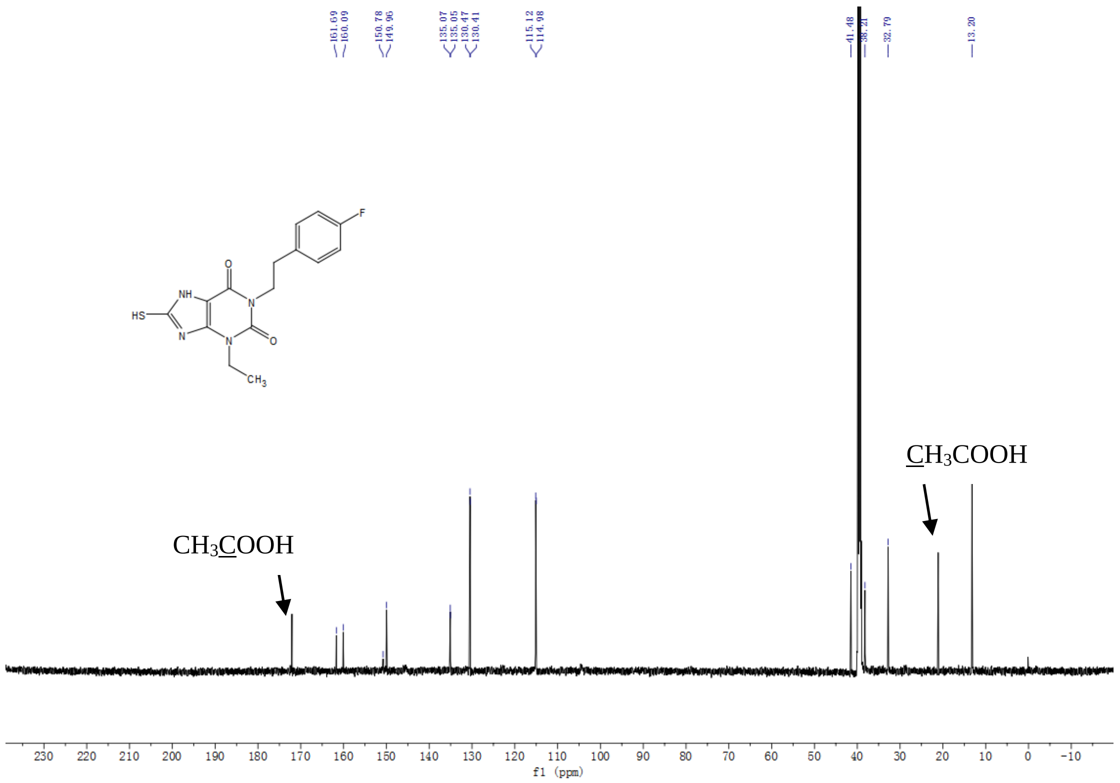

^{13}C NMR spectrum of compound **11f**

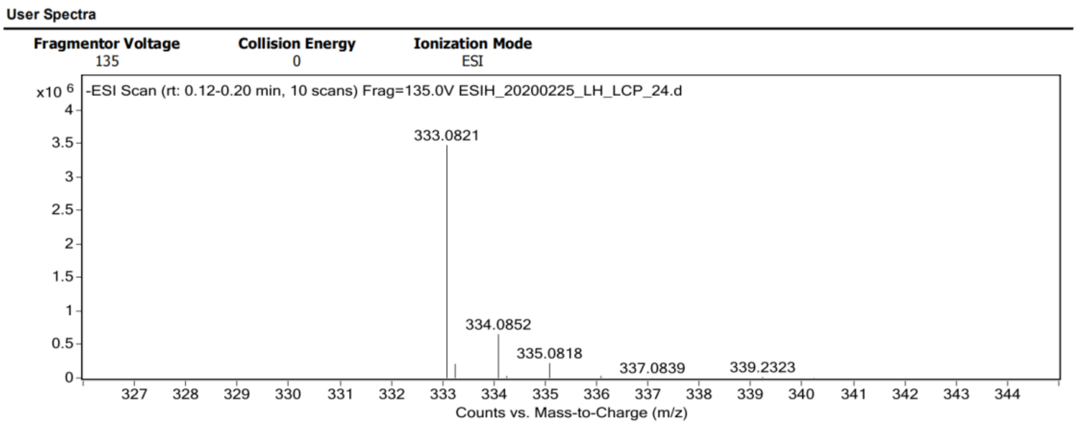

HRMS spectrum of compound **11f**

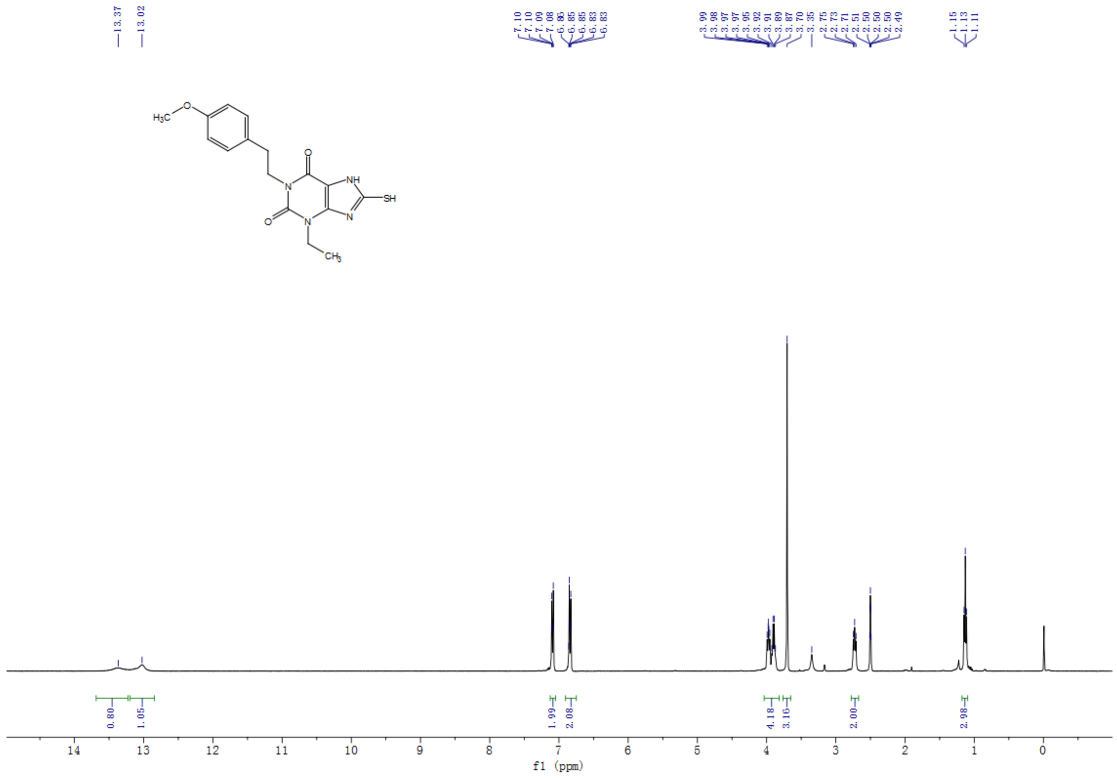

^{1}H NMR spectrum of compound **11g**

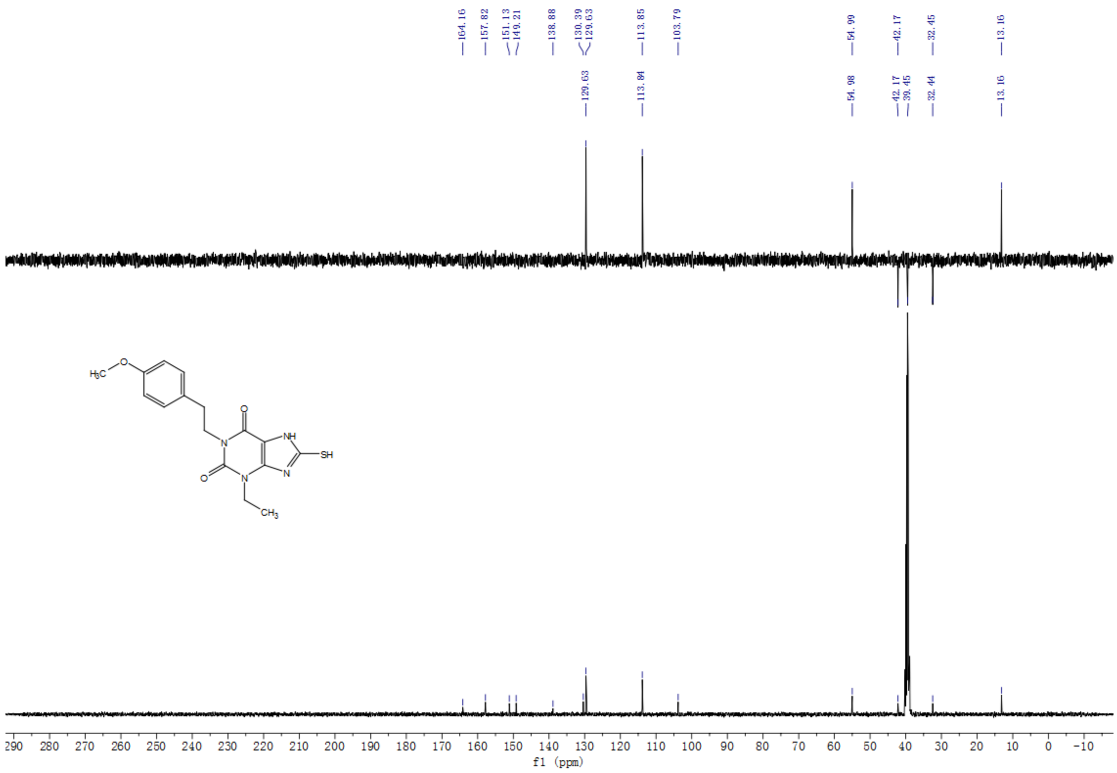

^{13}C NMR spectrum of (DEPT+BB) compound **11g**

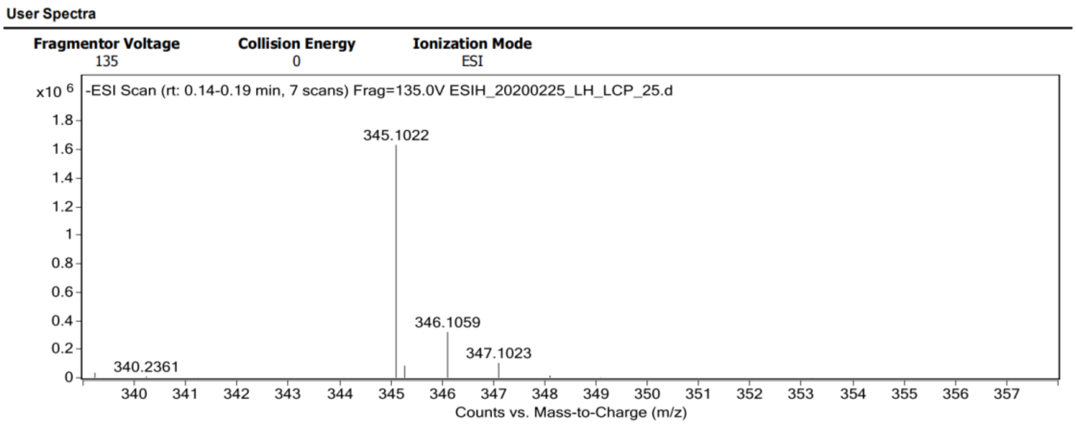

HRMS spectrum of compound **11g**

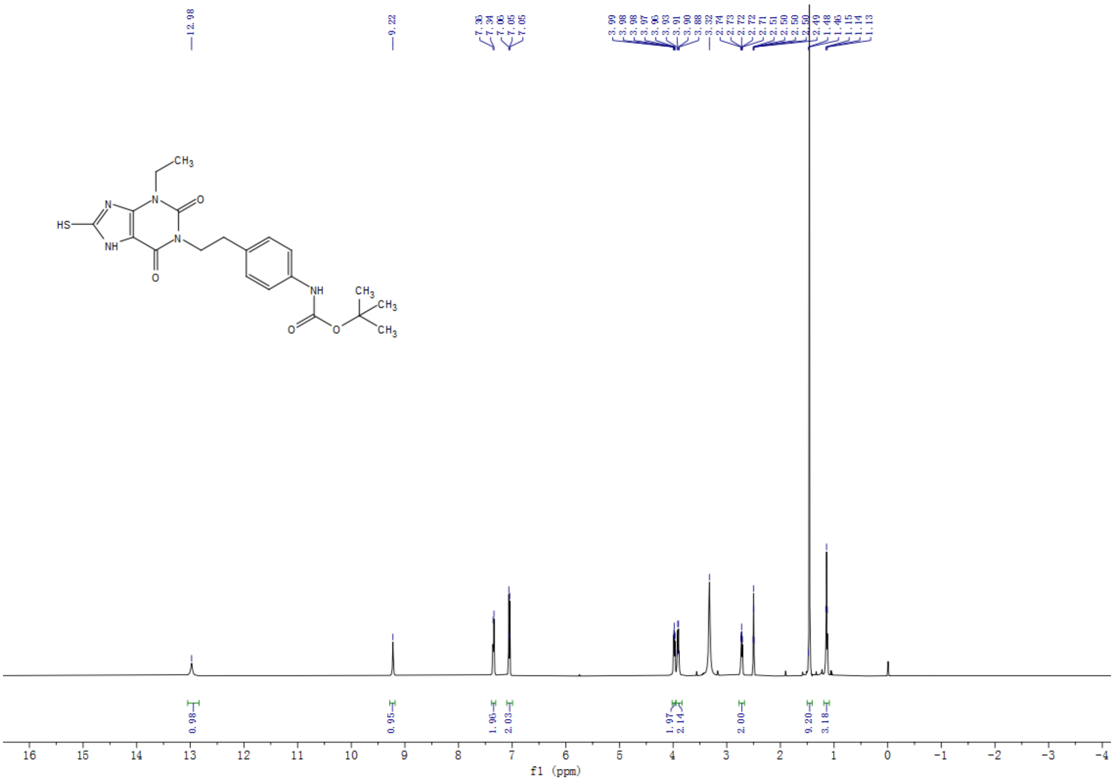

^{1}H NMR spectrum of compound **11h**

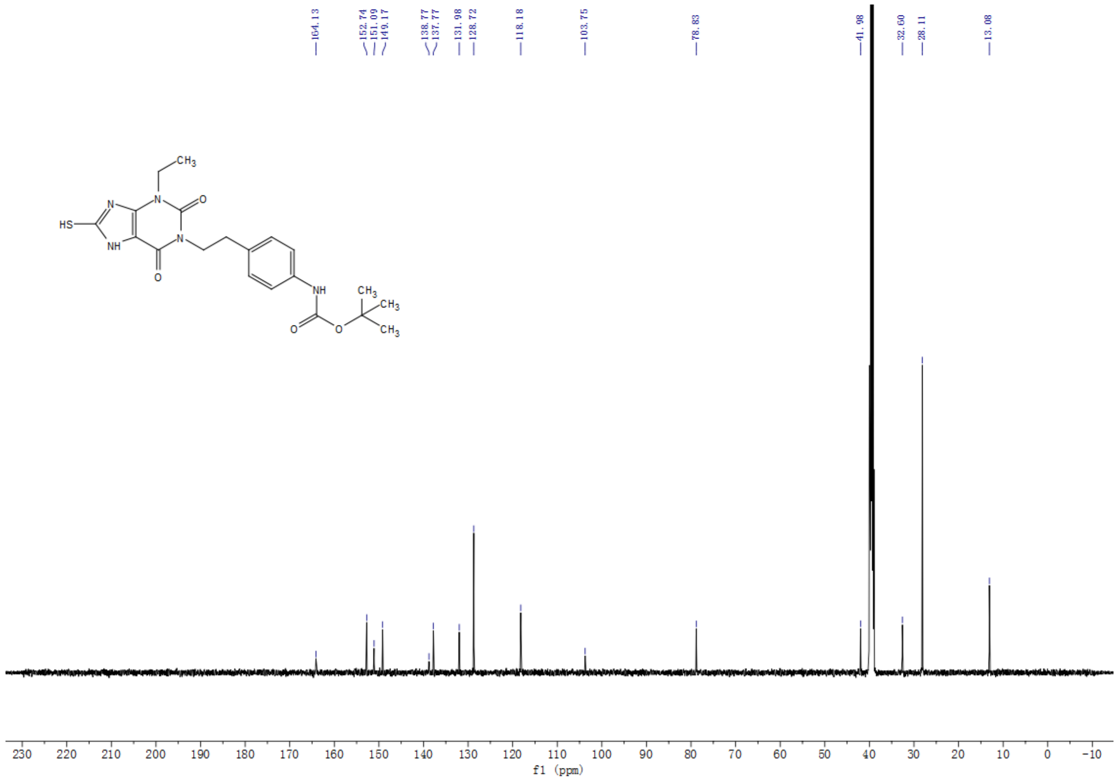

^{13}C NMR spectrum of compound **11h**

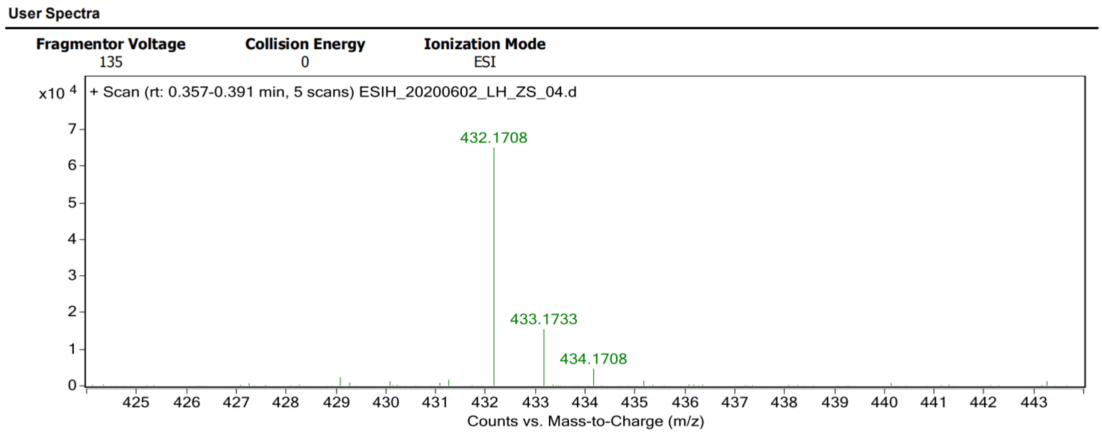

HRMS spectrum of compound **11h**

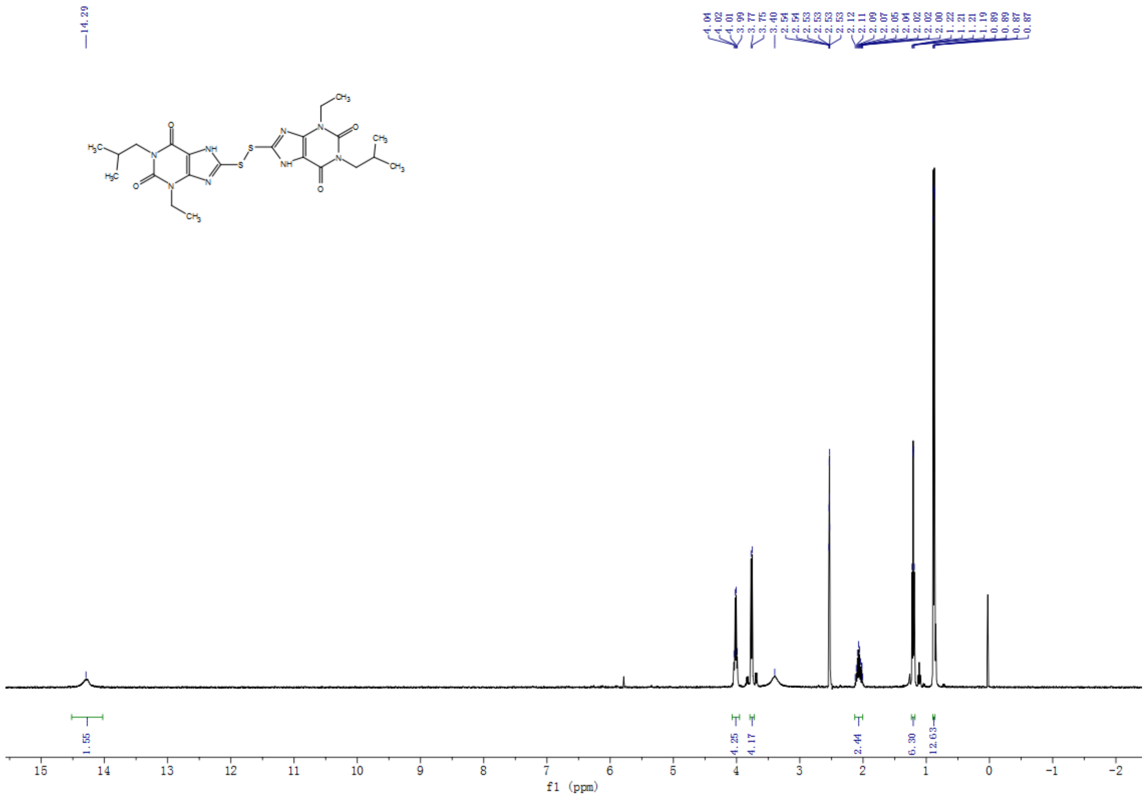

^{1}H NMR spectrum of compound **12**

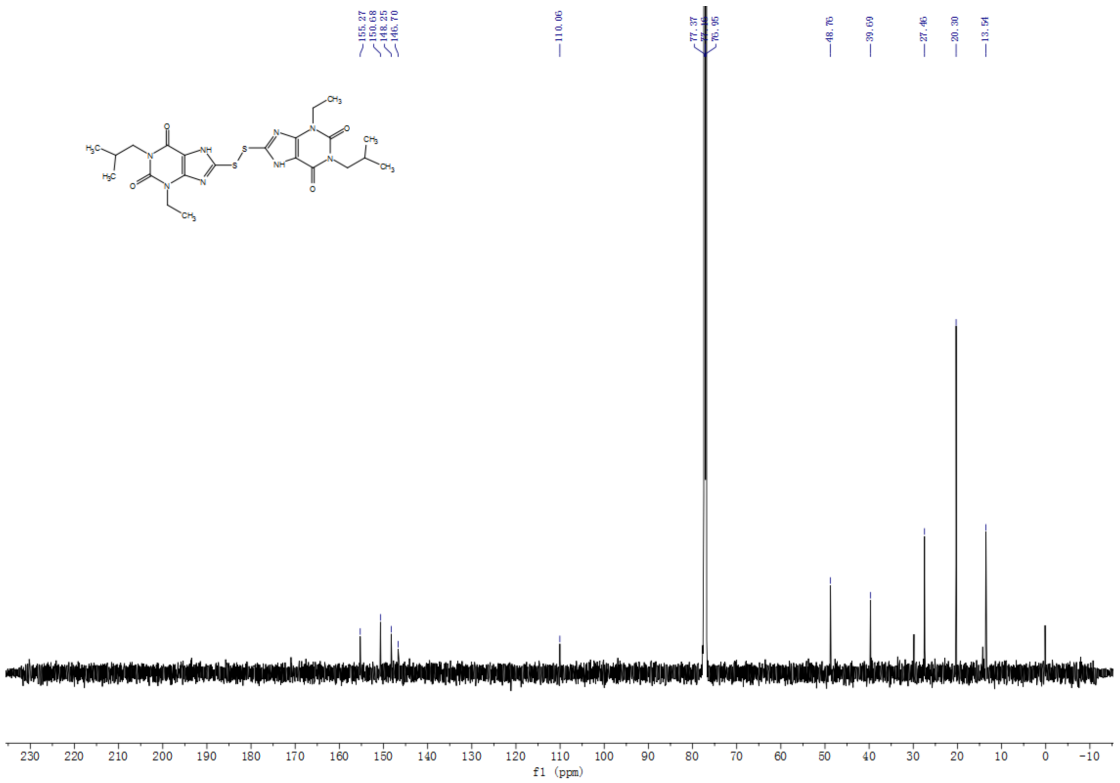

^{13}C NMR spectrum of compound **12**

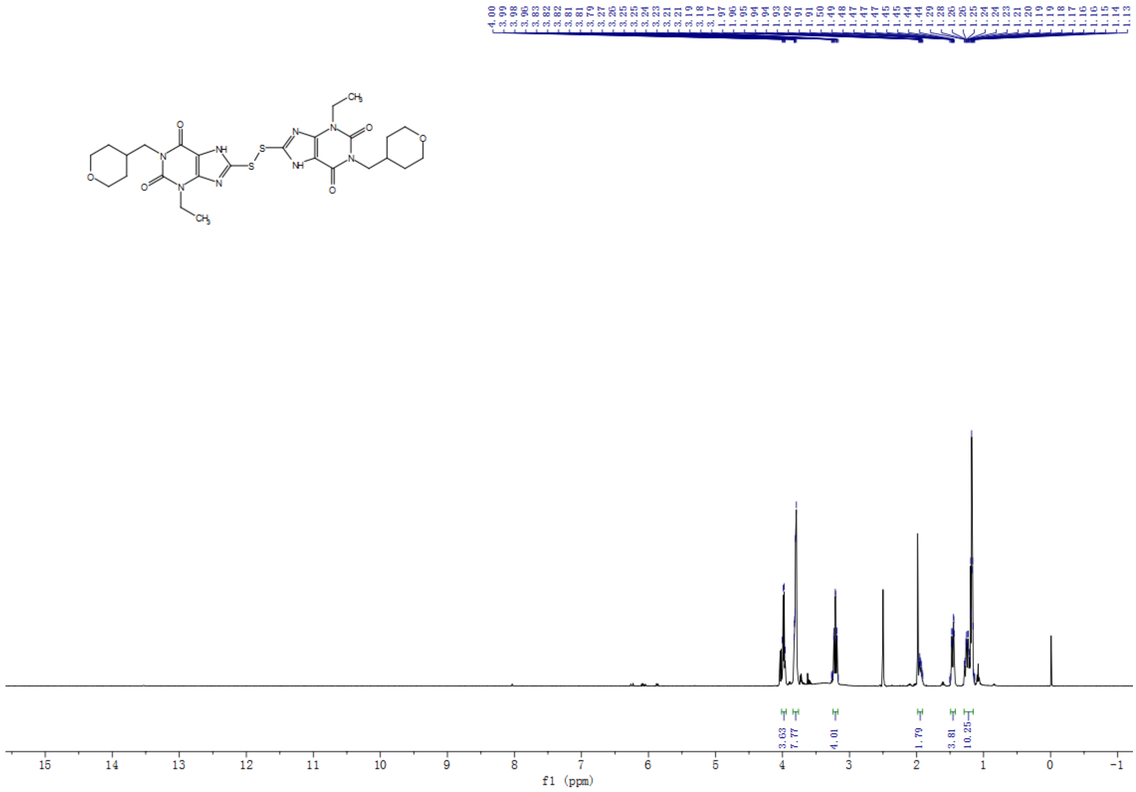

^{1}H NMR spectrum of compound **13**

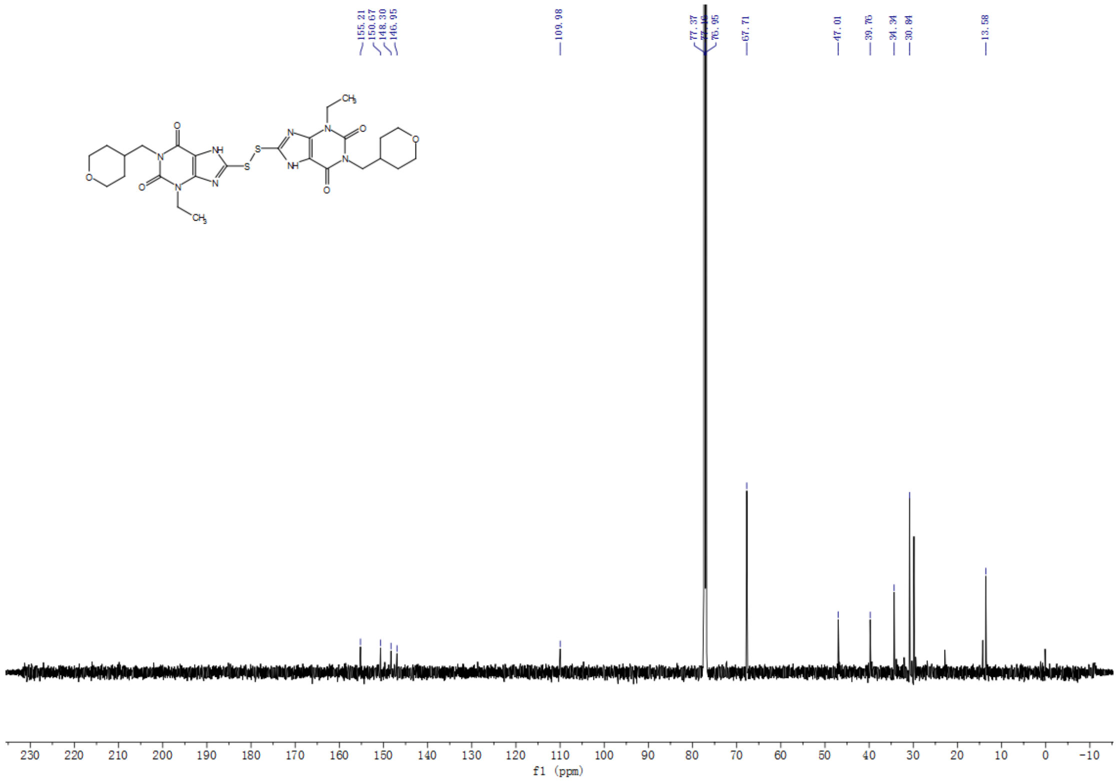

^{13}C NMR spectrum of compound **13**

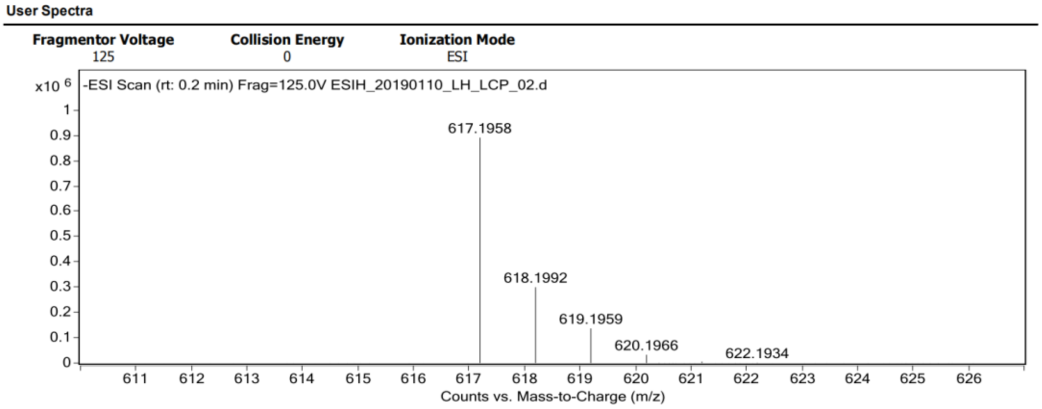

HRMS spectrum of compound **13**

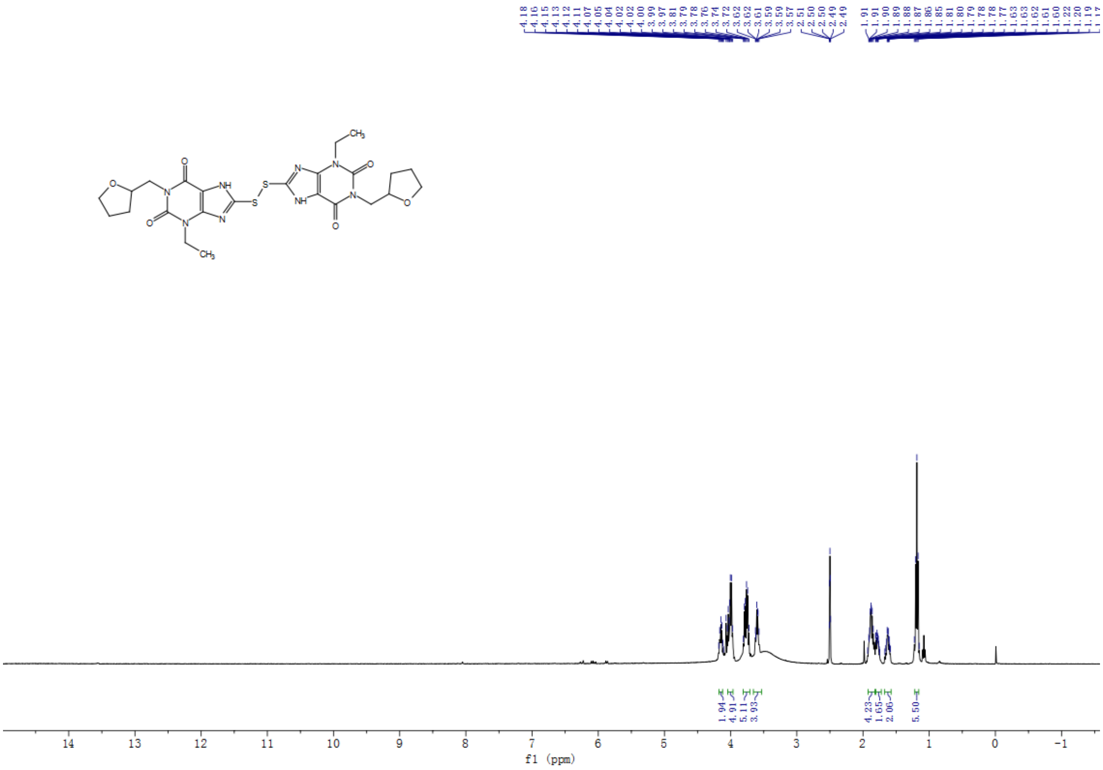

^{1}H NMR spectrum of compound **14**

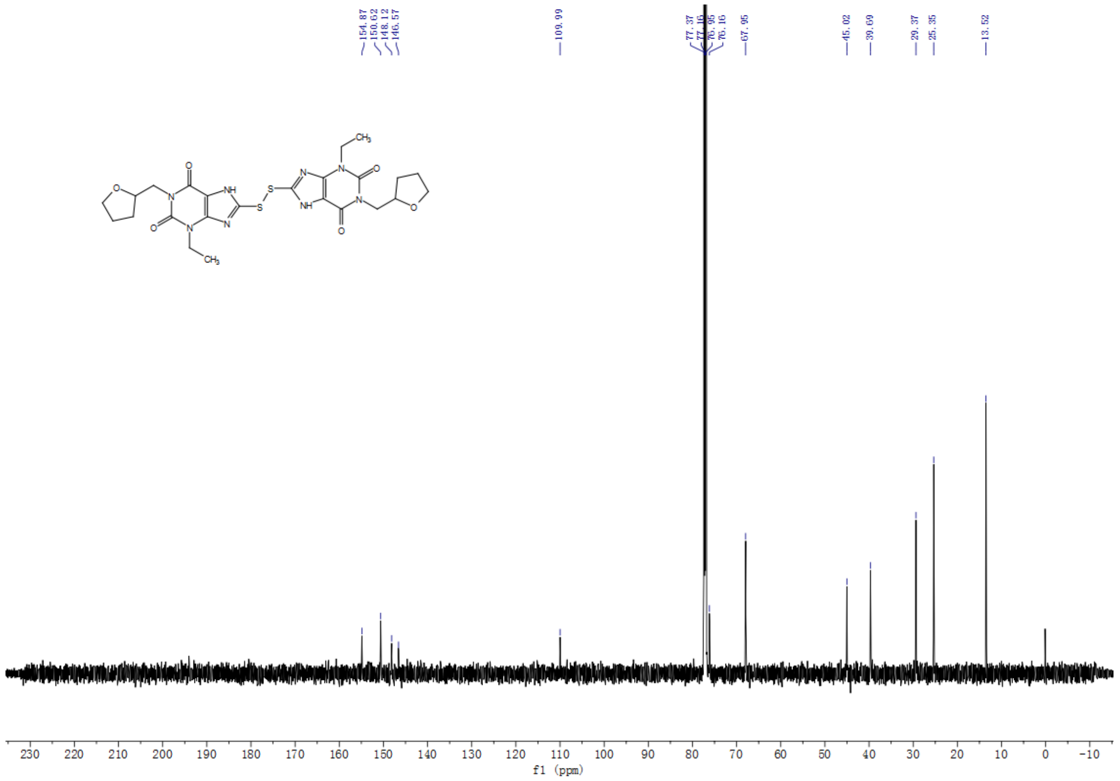

^{13}C NMR spectrum of compound **14**

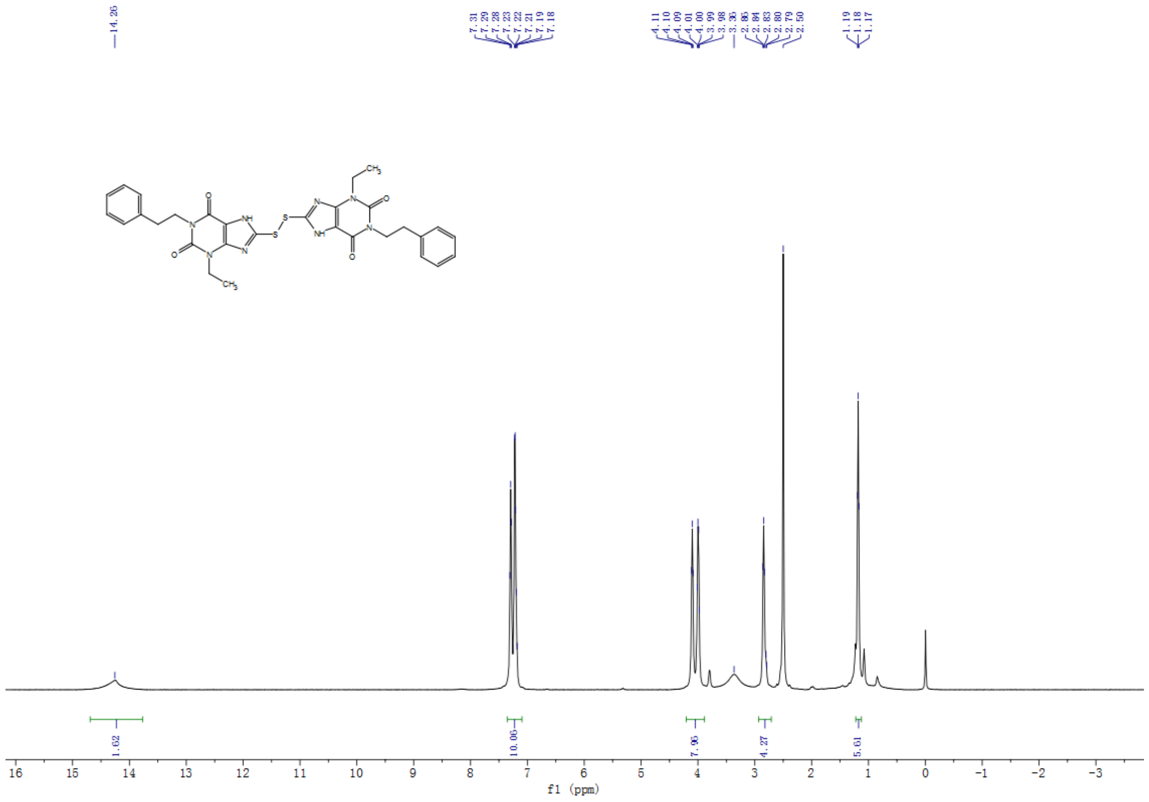

^{1}H NMR spectrum of compound **15**

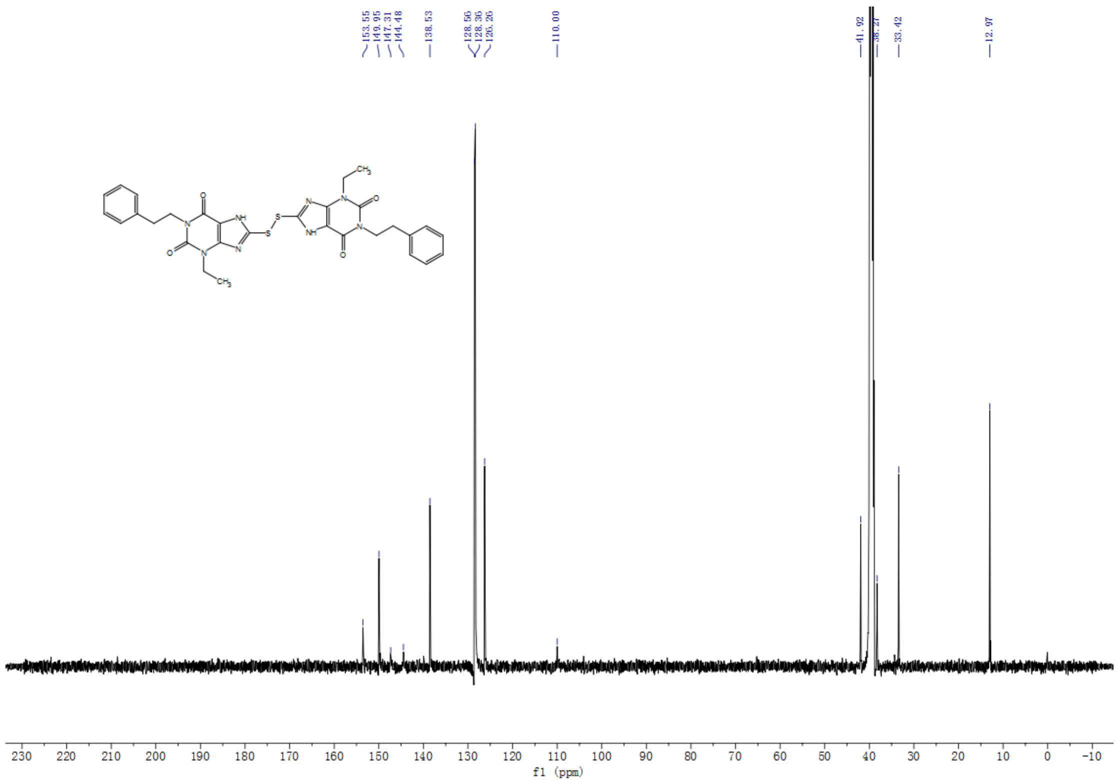

^{13}C NMR spectrum of compound **15**

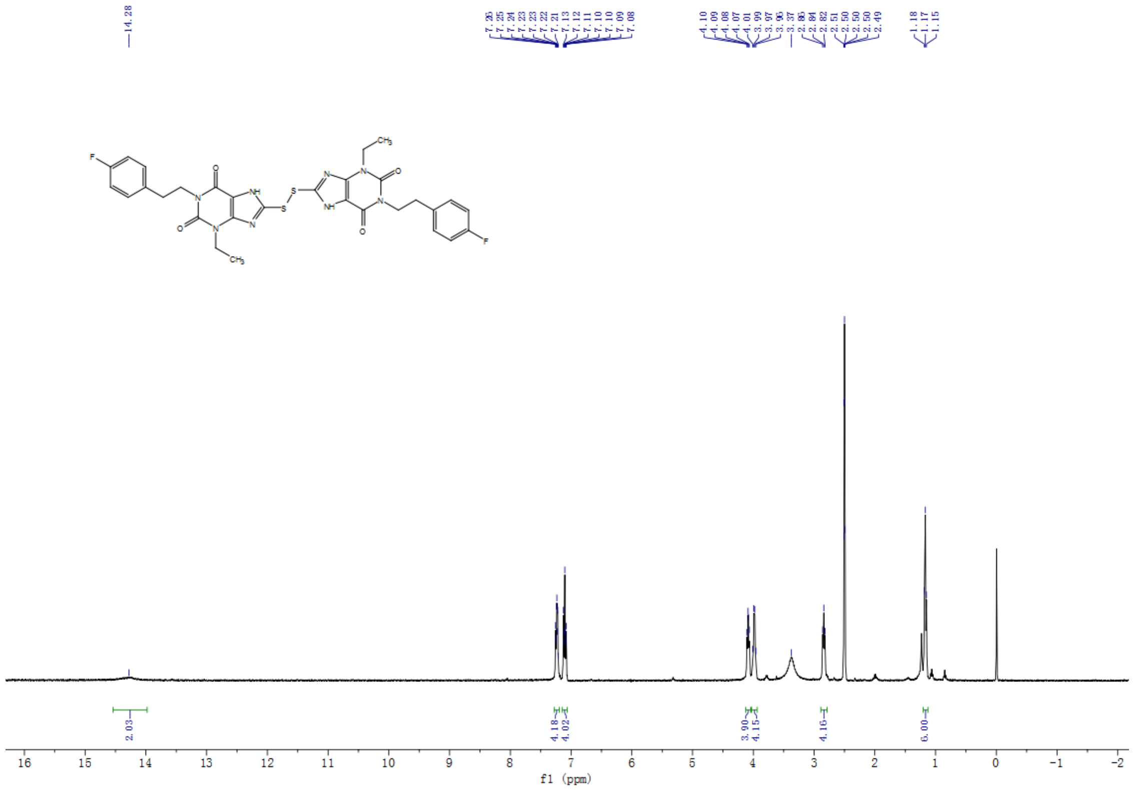

^{1}H NMR spectrum of compound **16**

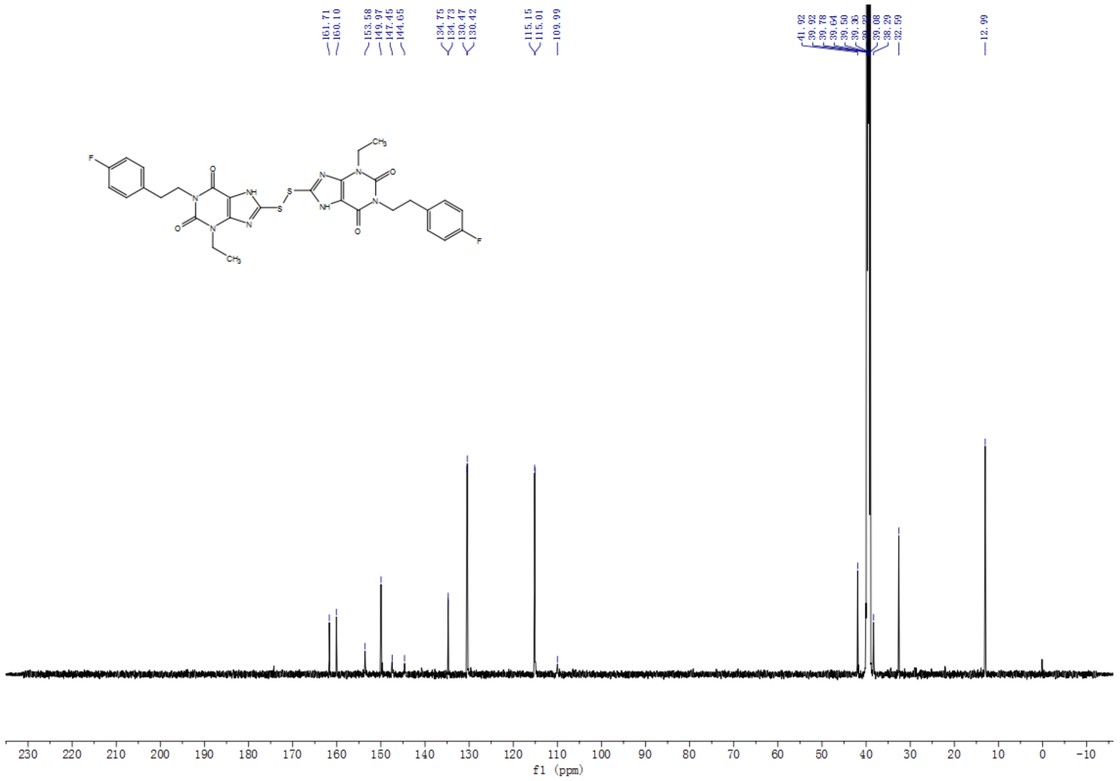

^{13}C NMR spectrum of compound **16**

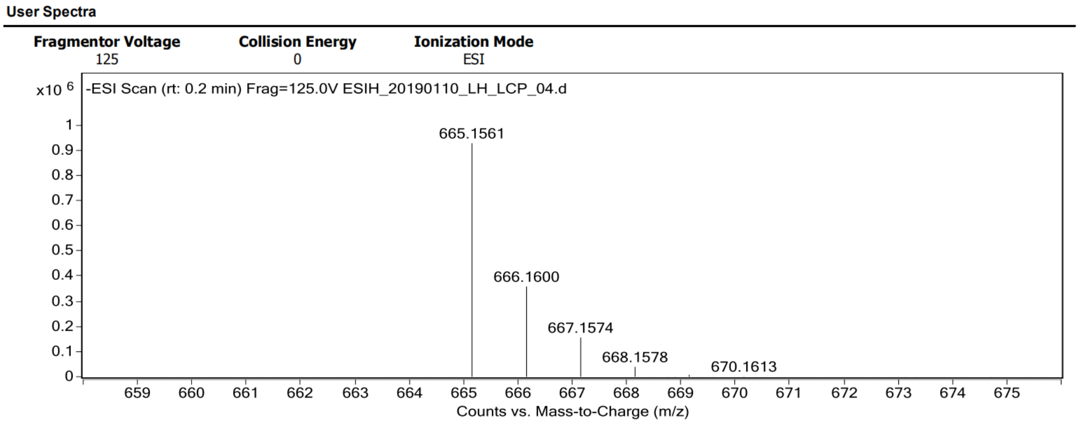

HRMS spectrum of compound **16**

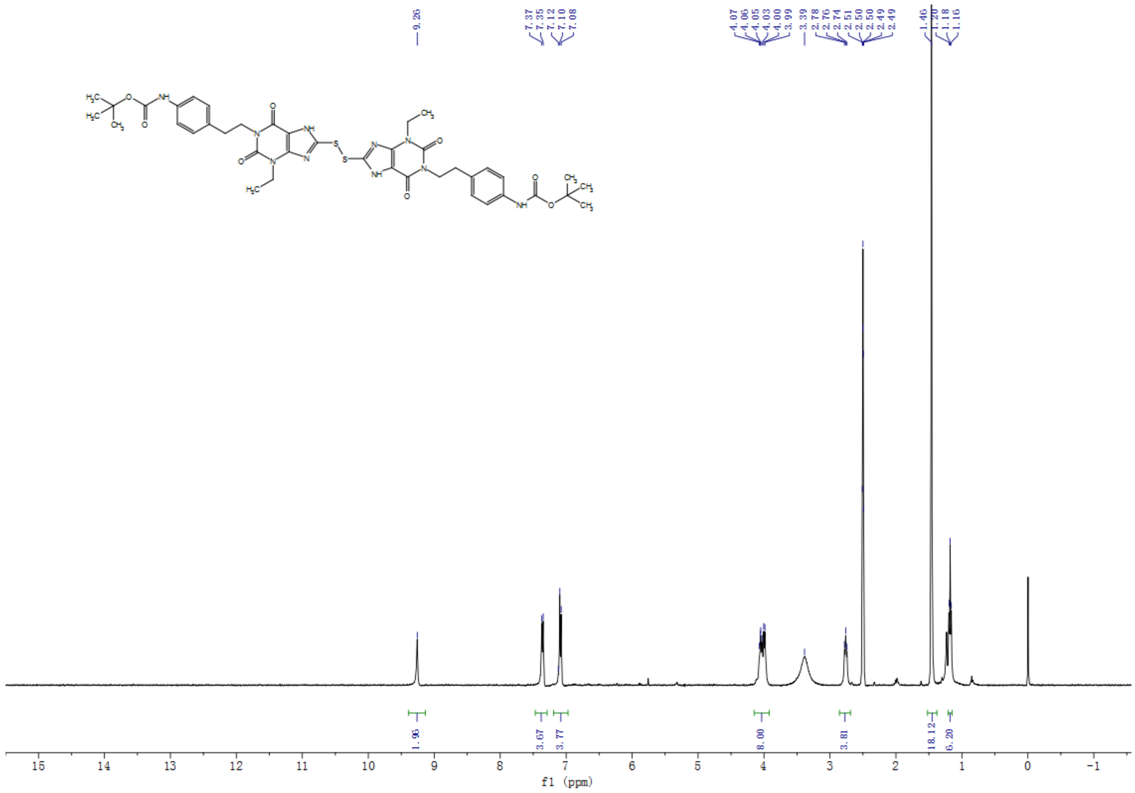

^{1}H NMR spectrum of compound **17**

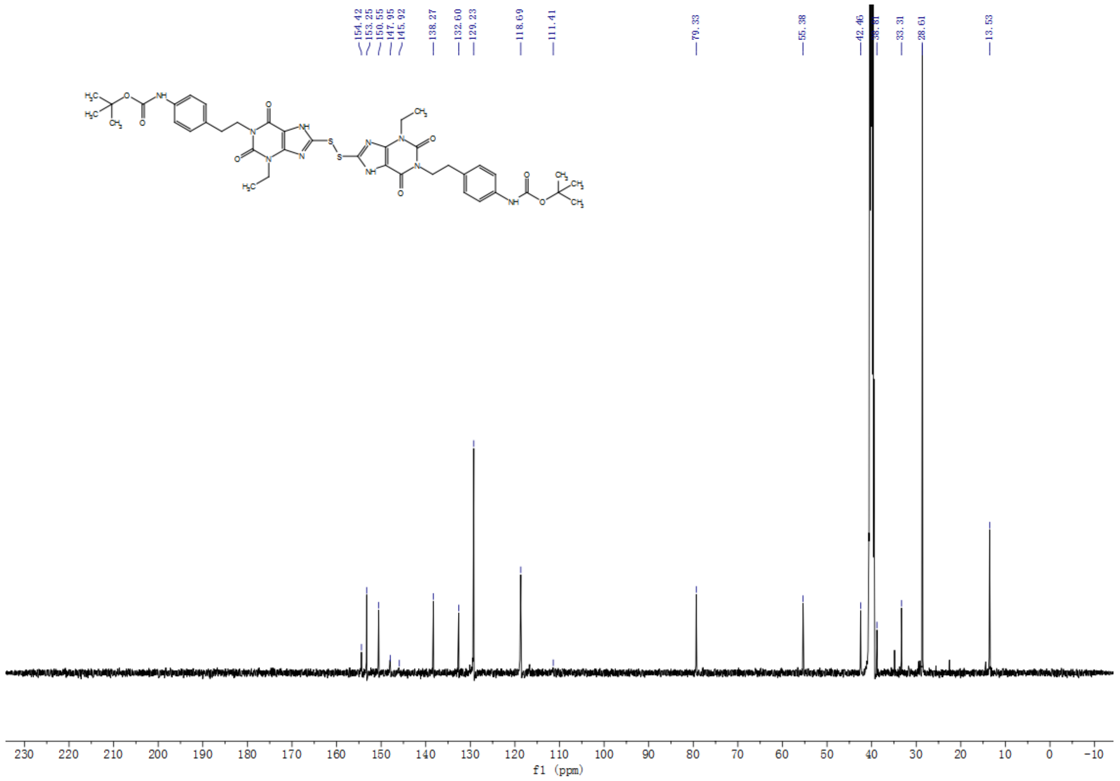

^{13}C NMR spectrum of compound **17**

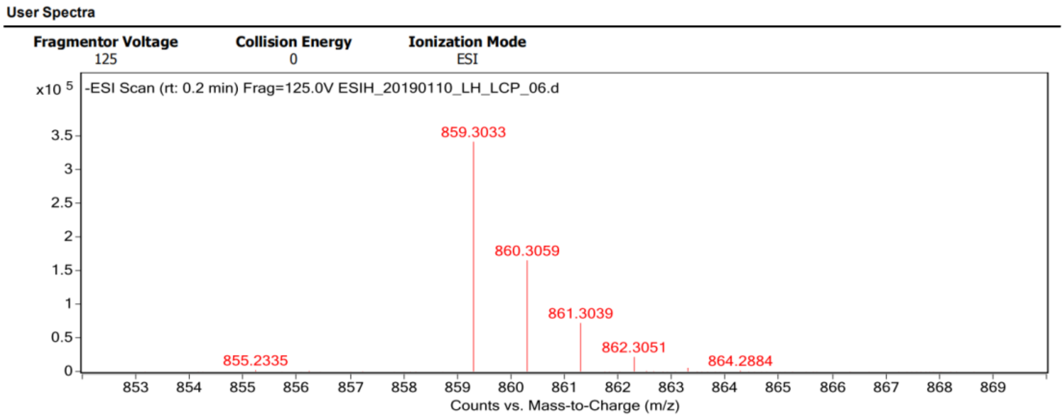

HRMS spectrum of compound **17**

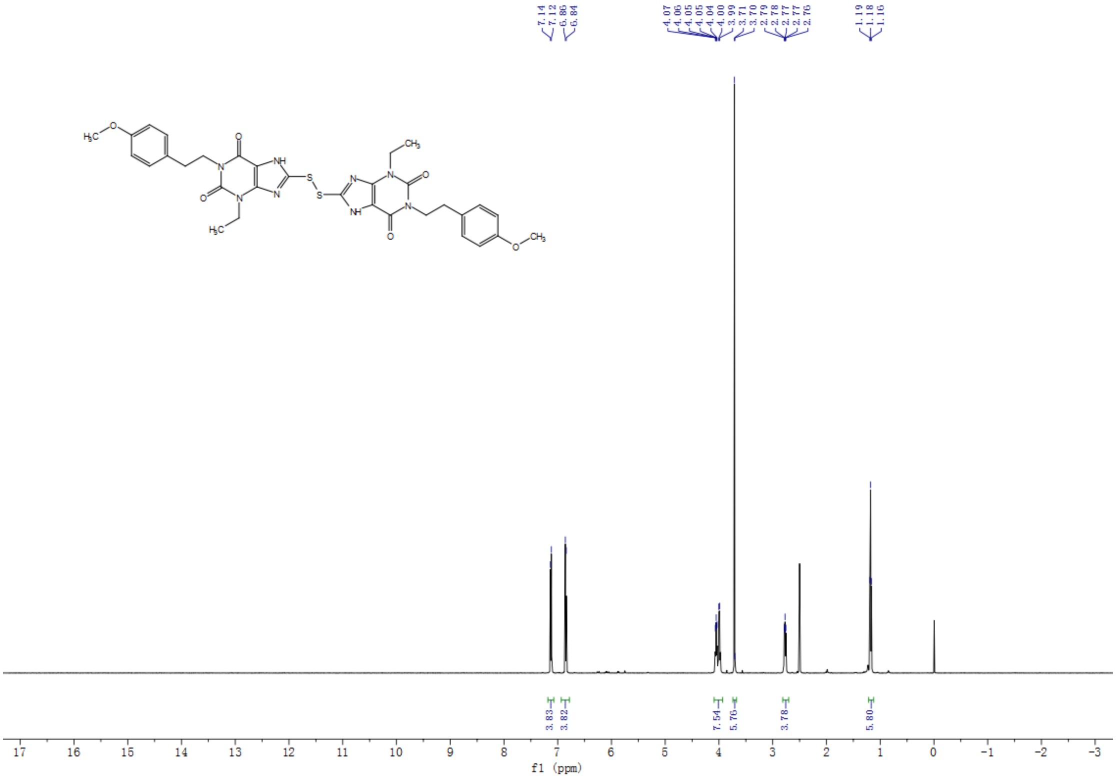

^{1}H NMR spectrum of compound **18**

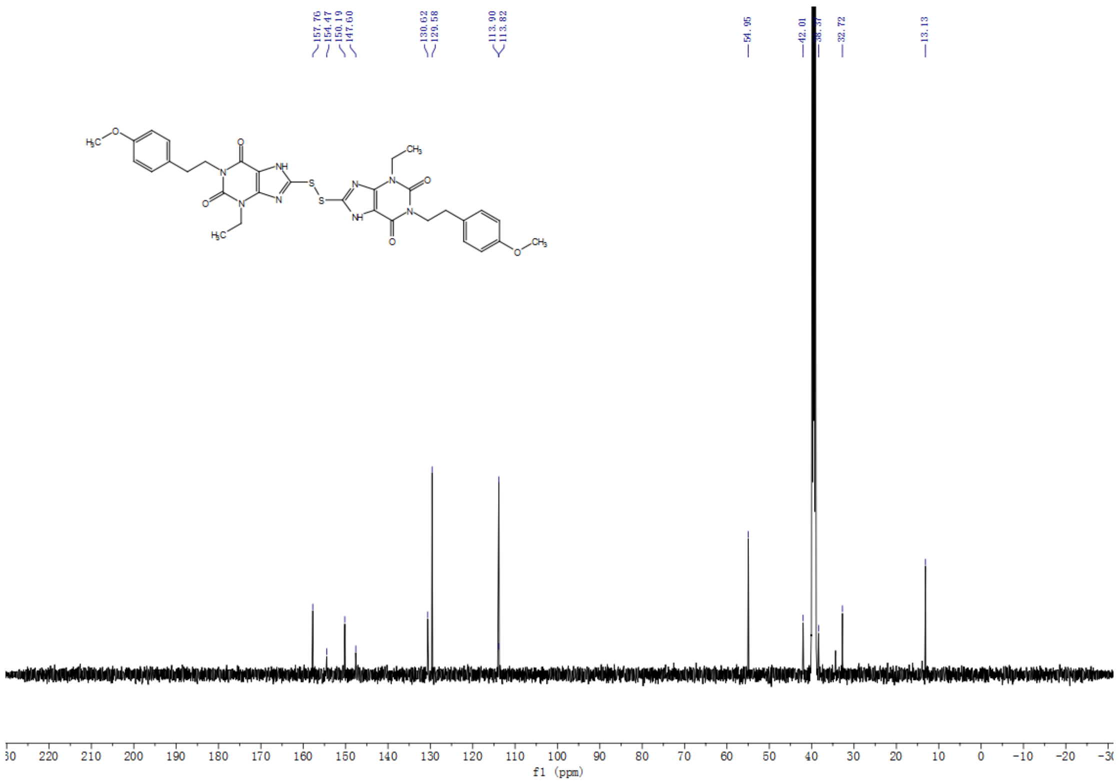

^{13}C NMR spectrum of compound **18**

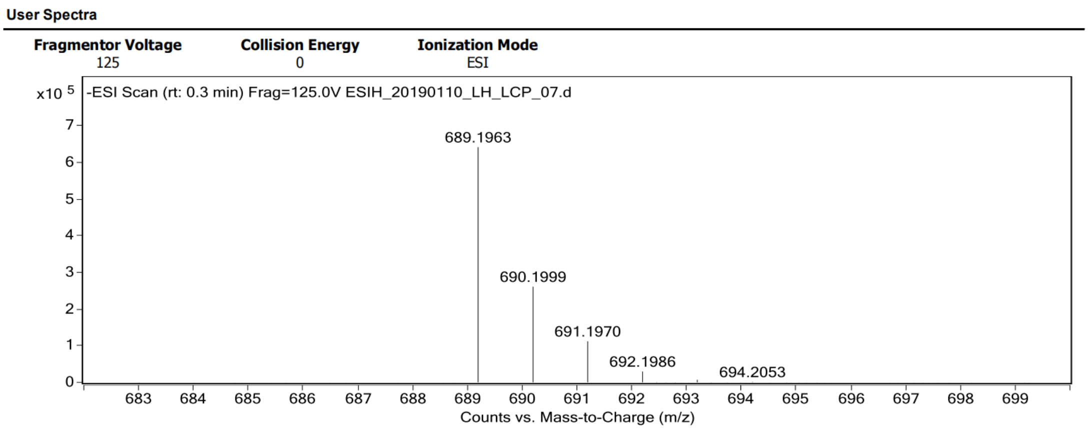

HRMS spectrum of compound **18**

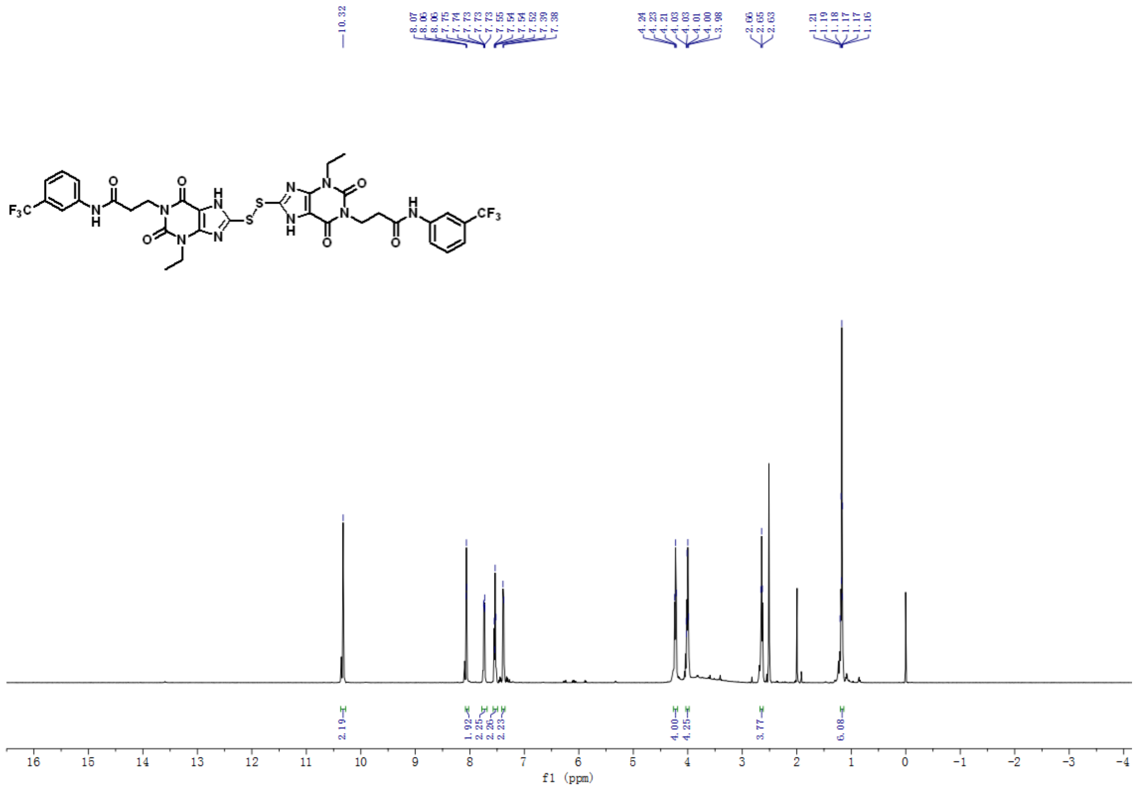

^{1}H NMR spectrum of compound **19**

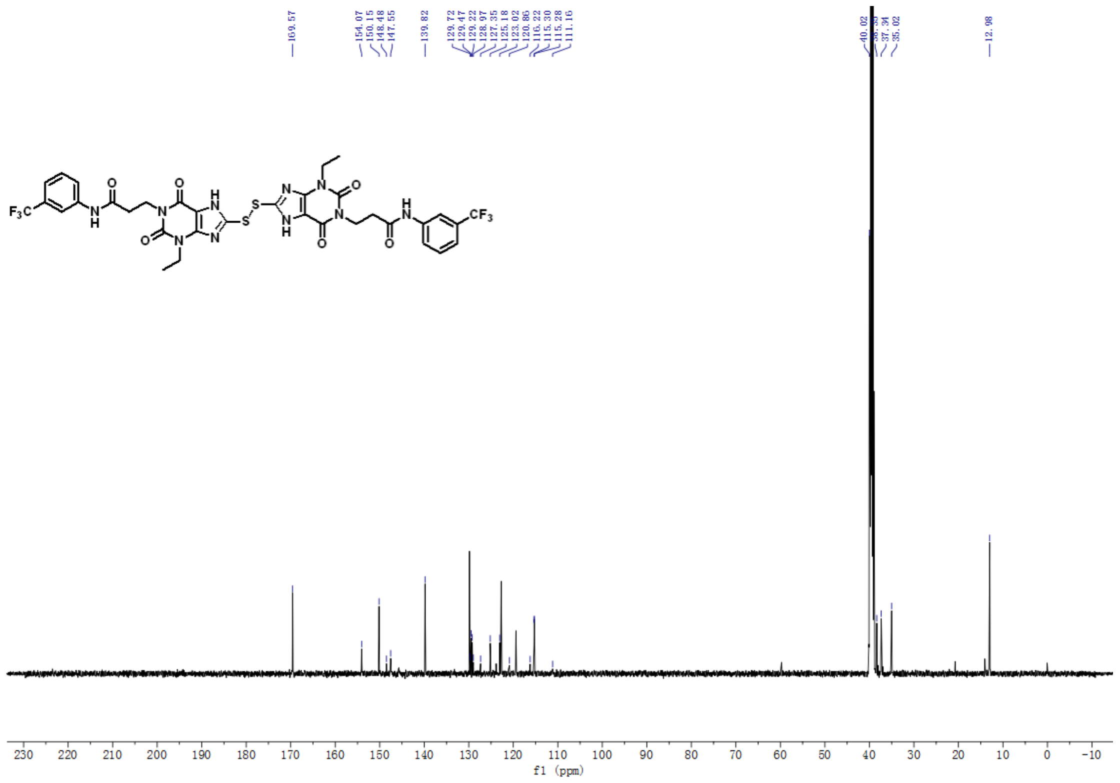

^{13}C NMR spectrum of compound **19**

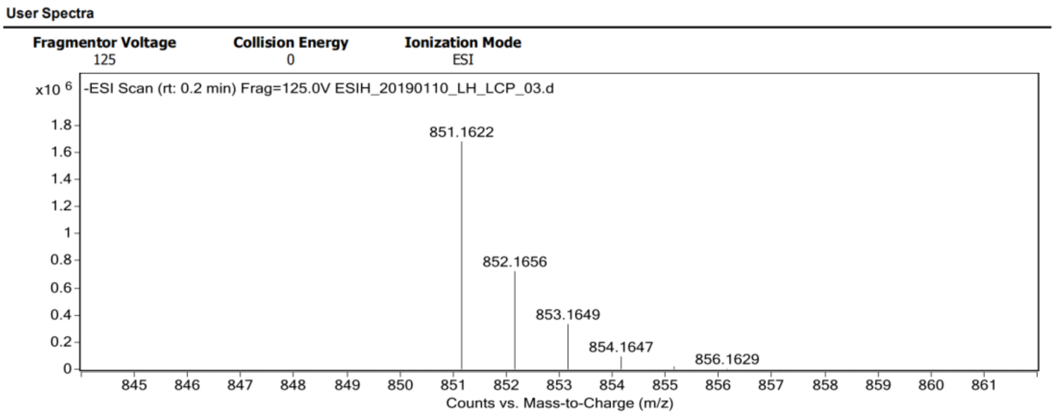

HRMS spectrum of compound **19**

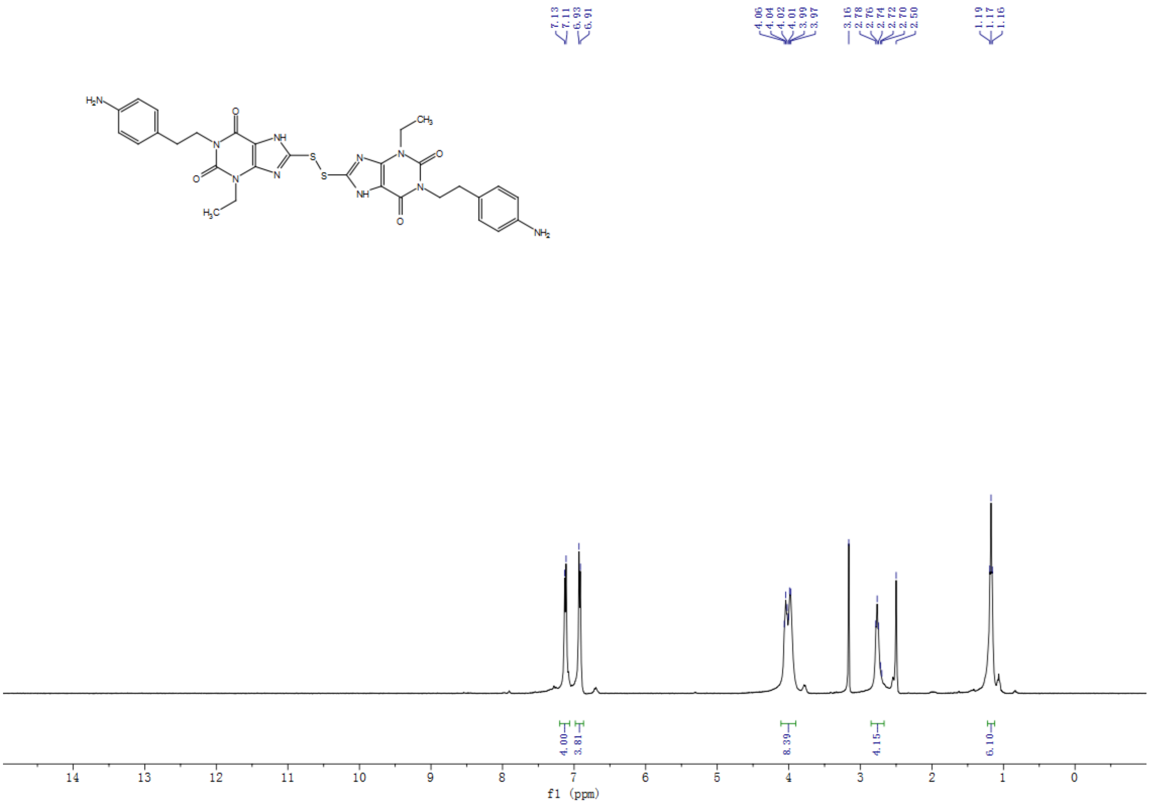

^{1}H NMR spectrum of compound **20**

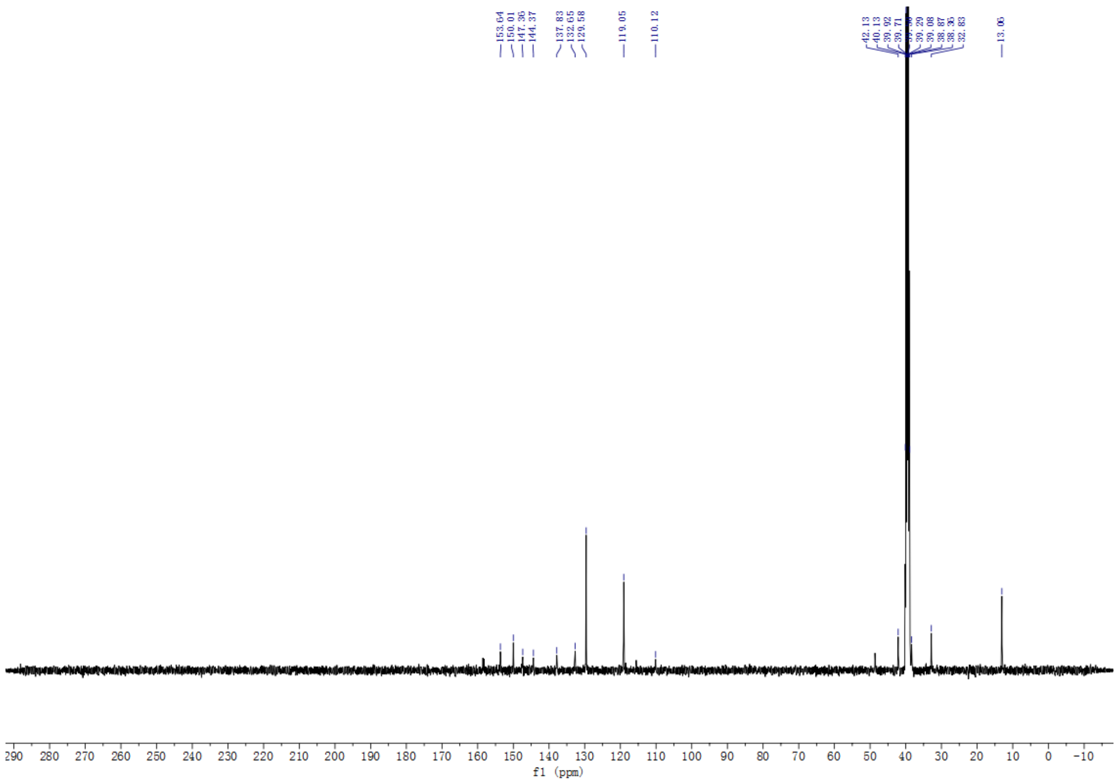

^{13}C NMR spectrum of compound **20**

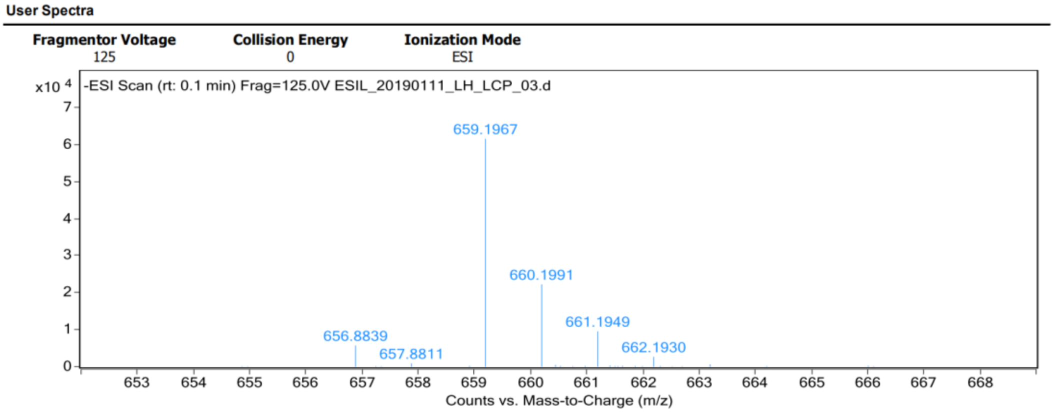

HRMS spectrum of compound **20**

Table S1 The HPLC analyses of all the target compounds.

HPLC Conditions A:

Column: Extend C-18 (5 µm, 4.6 x 150 mm)

Conditions:

Wavelength: 254 nm

Temperature: 25 °C

Flow rate: 1.00 mL/min

Eluent: MeOH/H_2O (70/30)

HPLC Conditions B:

Column: Extend C-18 (5 µm, 4.6 x 250 mm)

Conditions:

Wavelength: 254 nm

Temperature: 25 °C

Flow rate: 1.00 mL/min

Eluent: MeOH/H_2O (70/30)

HPLC Conditions C:

Column: Extend C-18 (5 µm, 4.6 x 250 mm)

Conditions:

Wavelength: 254 nm

Temperature: 25 °C

Flow rate: 1.00 mL/min

Eluent: MeOH/ 5 mmol NH_4OAc (60/40)

HPLC Conditions D:

Column: Extend C-18 (5 µm, 4.6 x 150 mm)

Conditions:

Wavelength: 254 nm

Temperature: 25 °C

Flow rate: 1.00 mL/min

Eluent: MeOH/ 5 mmol NH_4OAc (60/40)

Compound	Retention time (min)	Relative purity (%)
1	10.791[C]	98.7871
4	2.743[A]	98.1972
12	26.201[D]	96.2007
13	3.042[B]	95.6679
14	1.677[A]	95.8527
15	11.755[B]	98.1286
16	13.064[B]	98.3703
17	8.364[A]	97.9333
18	9.166[B]	96.2227
19	2.714[A]	97.2012
20	5.443[D]	95.7640

Figure S1

A) 2D schematic representation of the interaction of **1** with SIRT3

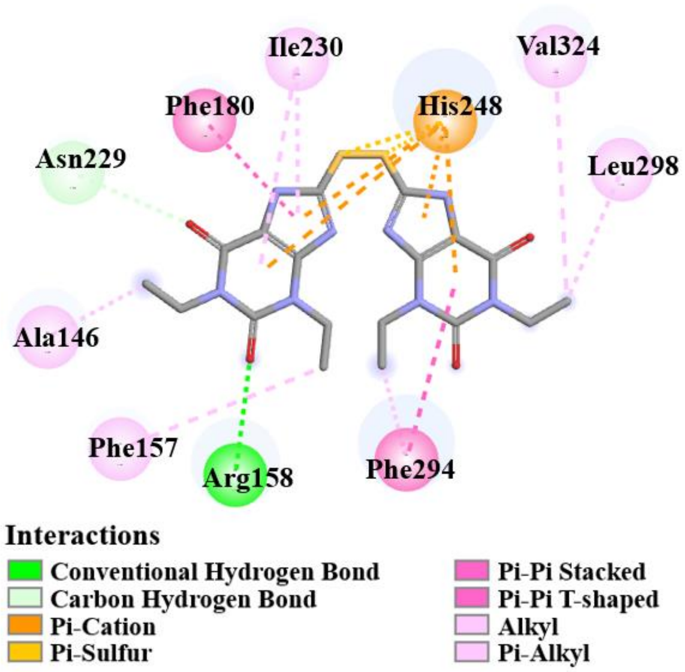

B) 2D schematic representation of the interaction of **4** with SIRT3

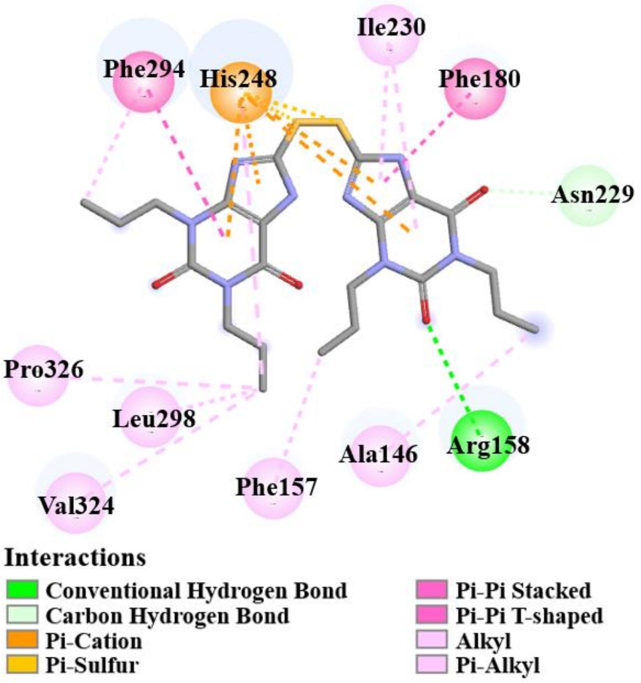

C) 2D schematic representation of the interaction of **12** with SIRT3

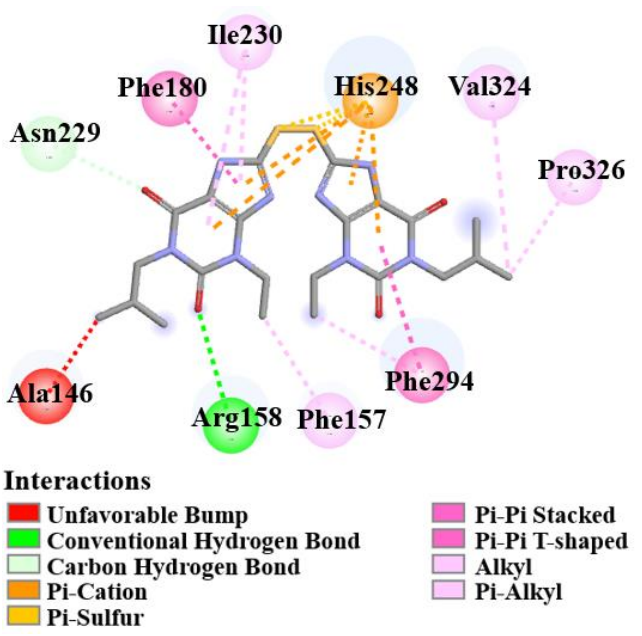

D) 2D schematic representation of the interaction of **13** with SIRT3

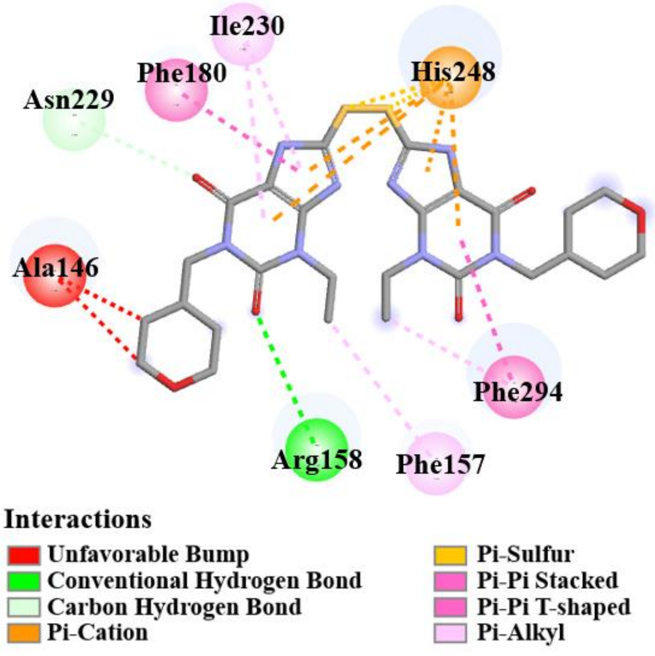

E) 2D schematic representation of the interaction of **14** with SIRT3

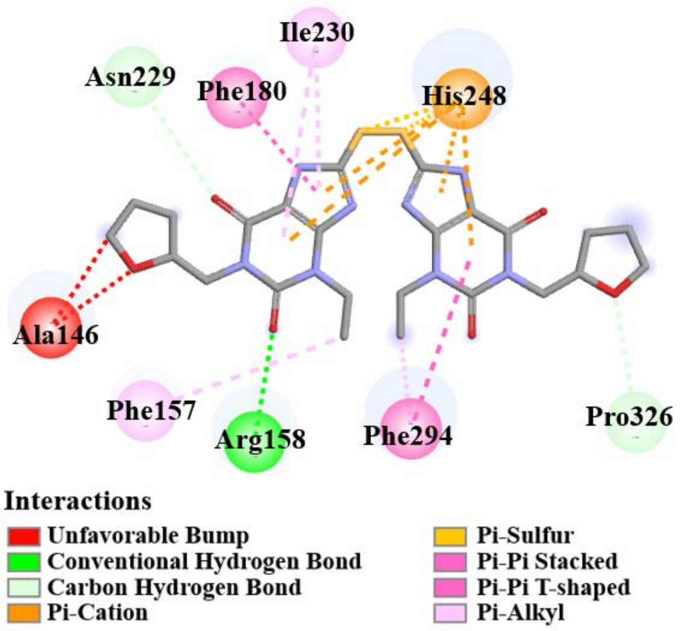

F) 2D schematic representation of the interaction of **16** with SIRT3

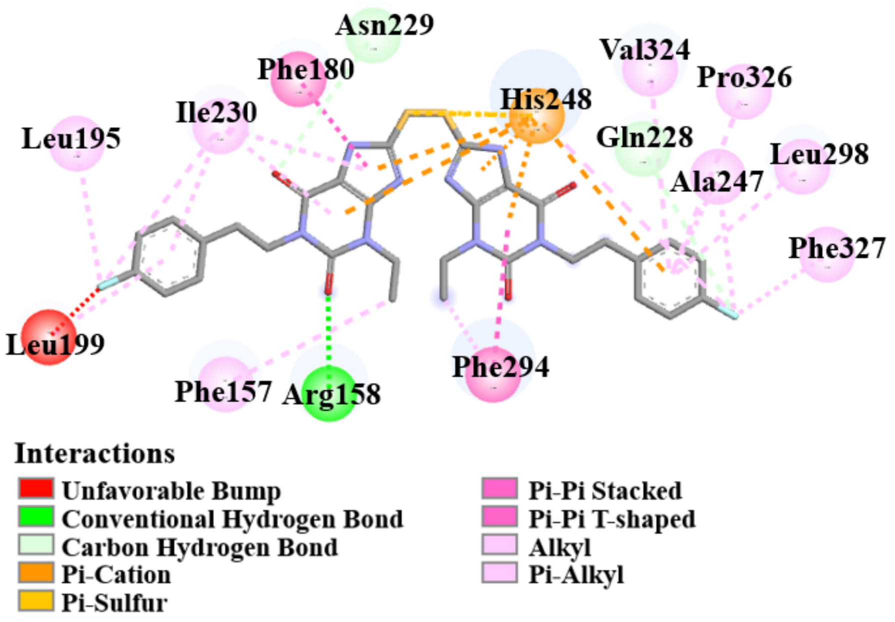

G) 2D schematic representation of the interaction of **17** with SIRT3

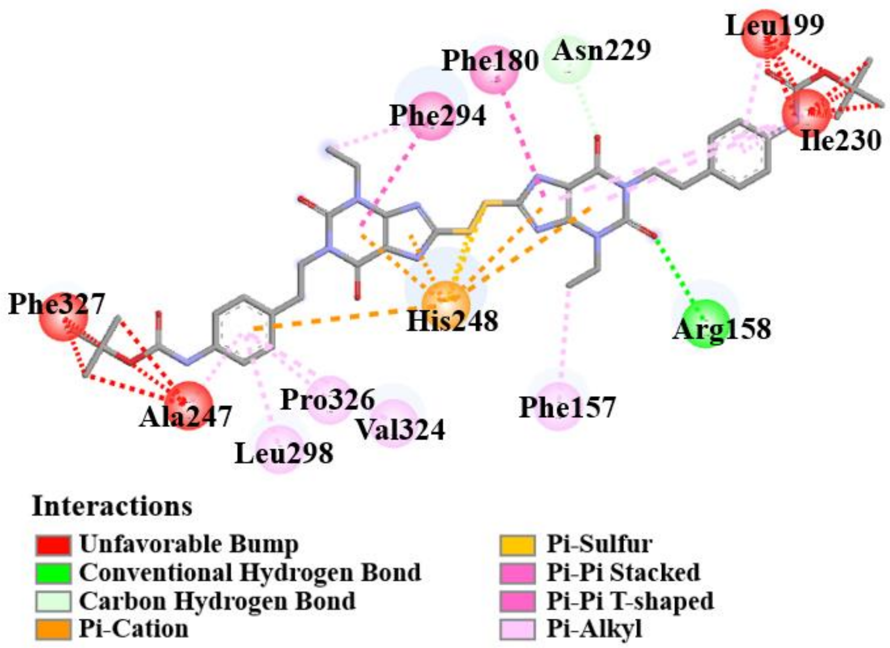

H) 2D schematic representation of the interaction of **18** with SIRT3

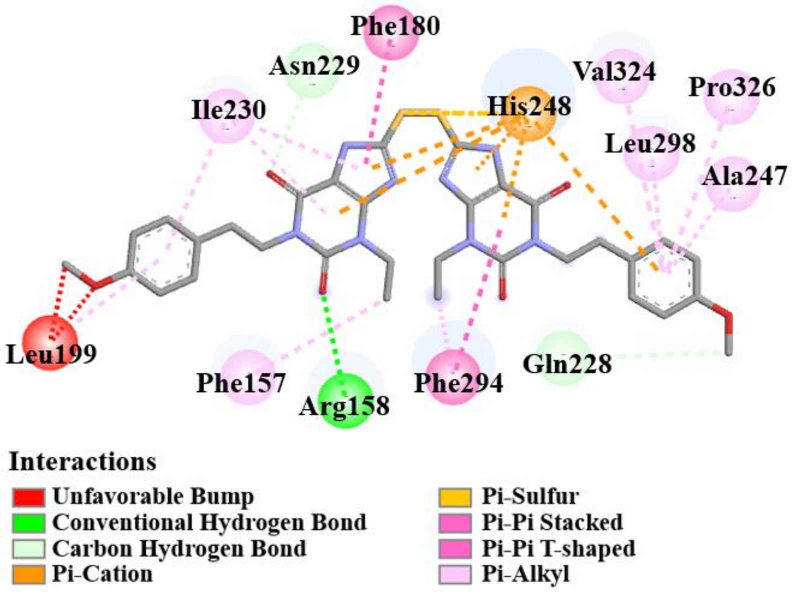

I) 2D schematic representation of the interaction of **19** with SIRT3

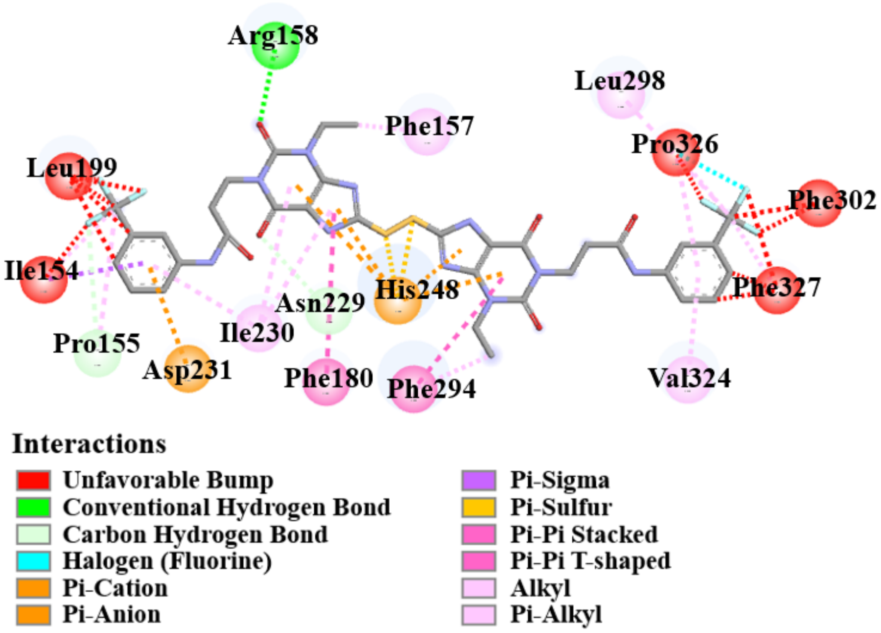

J) 2D schematic representation of the interaction of **20** with SIRT3

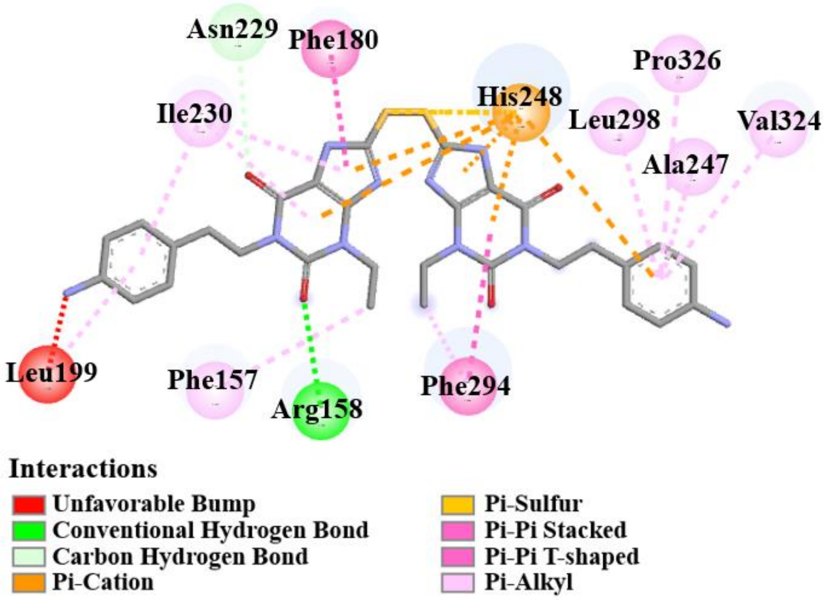

Figure S2

To evaluate the toxicity of the compounds, we carried out MTT assays on SH-SY5Y cells incubated with various concentrations of compounds **4** and **15** for 24 hours. We found that **4** and **15** were nontoxic at concentrations up to 20 μM and displayed toxicity at 50 μM (as shown in the figure below).

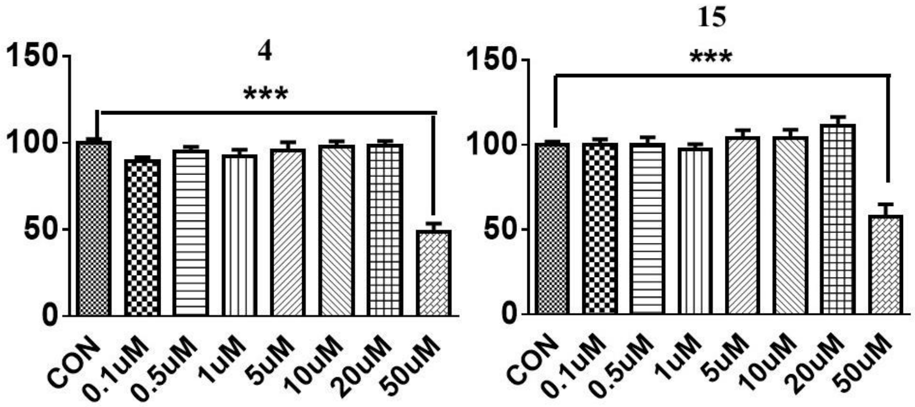

Figure S2 MTT assays to evaluate the toxicity of compound **4** and **15** on SH-SY5Y cells

Figure S3

To examine the stability of the compound, we analyzed the solution sample of compound **1** prepared in DMSO nine months ago (stored in refrigerator, 4 °C) by mass spectrometry. As shown in Figure **S3A** and **S3B**, we can see that the compound remains stable in DMSO even after nine months. Further, we determined the compound in the assay buffer without SIRT3, and the compound after the deacetylation catalyzed by SIRT3 by using HPLC. As shown in Figure **S3C** and **S3D**, the compound is stable in the assay buffer. To address whether SIRT3 might cause any chemical reaction of the compound, we analyzed compound **1** in the assay buffer with SIRT3 and found that the compound did not have any change after the deacetylation catalyzed by SIRT3 (Figure **S3C** and **S3E**). These results demonstrated that the compound is stable in the activity assays.

A)

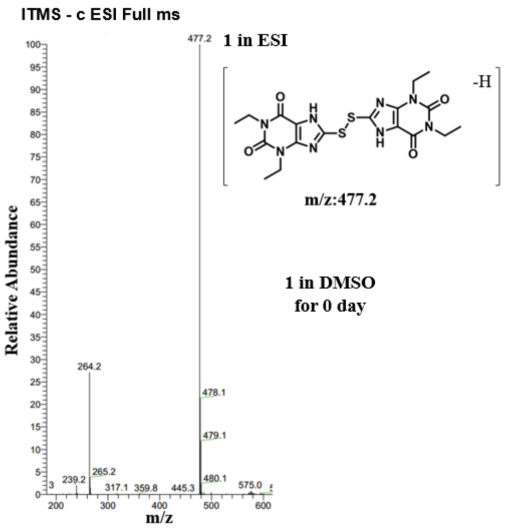

B)

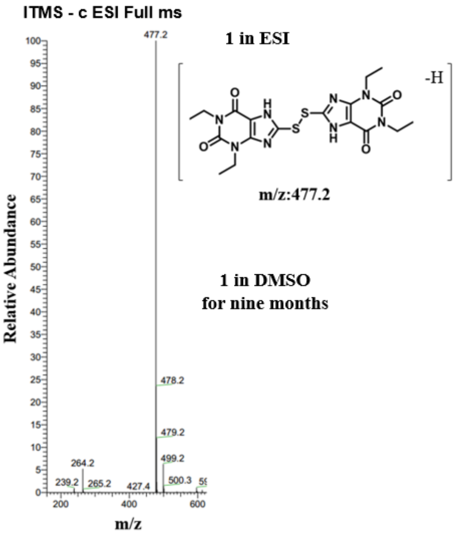

C)

D)

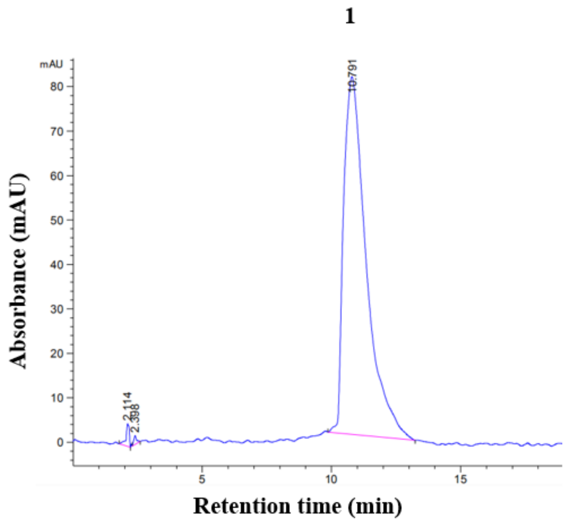

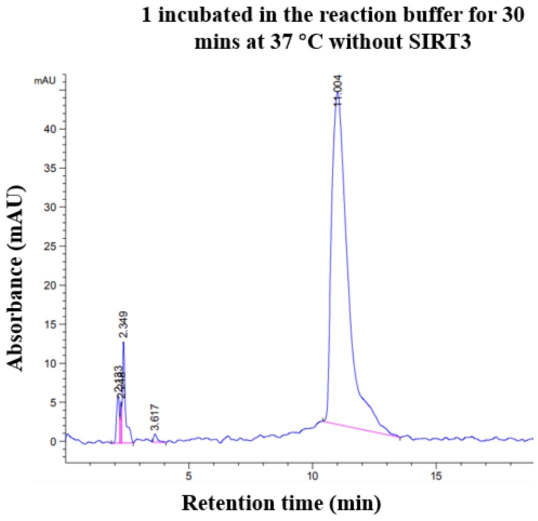

E)

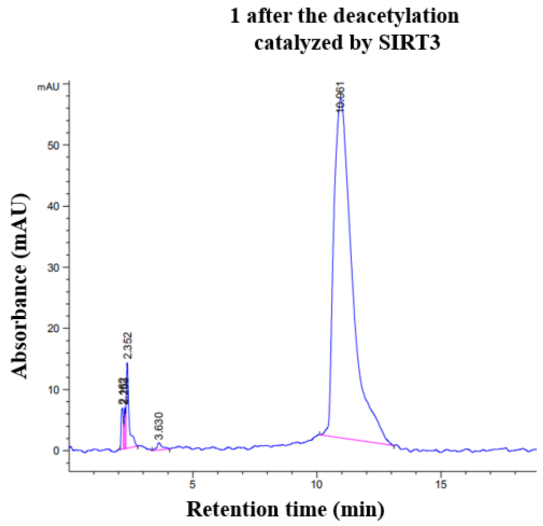

Figure S3 Compound **1** is stable in DMSO and in the activity assays. (**A**) Mass spectra of compound **1** in DMSO for 0 day (**B**) or for nine months. (**C**) HPLC trace (monitored at 254 nm) for **1** as a control, showing the relative purity of **1**: 98.7871 (%). (**D**) Compound **1** incubated in the assay buffer (25 mM Tris.HCl, pH 8.0, 137 mM NaCl, 2.7 mM KCl, 1 mM $MgCl_2$) for 30 min at 37 °C without SIRT3. The HPLC trace showed the relative purity of **1**: 91.2655 (%). (**E**) The solution sample of **1** after deacetylation catalyzed by SIRT3. The HPLC trace showed the relative purity of **1**: 93.7192 (%).
